# Placebo response and effect in randomized clinical trials: meta-research with focus on contextual effects

**DOI:** 10.1186/s13063-021-05454-8

**Published:** 2021-07-26

**Authors:** Sigurlaug H. Hafliðadóttir, Carsten B. Juhl, Sabrina M. Nielsen, Marius Henriksen, Ian A. Harris, Henning Bliddal, Robin Christensen

**Affiliations:** 1 Section for Biostatistics and Evidence-Based Research, the Parker Institute, Bispebjerg and Frederiksberg Hospital, Nordre Fasanvej 57, DK-2000 Copenhagen F, Denmark; 2 SEARCH Research Group, Research Unit of Musculoskeletal Function and Physiotherapy, Institute of Sports Science and Clinical Biomechanics, Faculty of Health Sciences, University of Southern Denmark, Odense, Denmark; 3 Department of Physiotherapy and Occupational Therapy, University Hospital of Copenhagen, Herlev, Gentofte Denmark; 4 Research Unit of Rheumatology, Department of Clinical Research, University of Southern Denmark, Odense University Hospital, Odense, Denmark; 5 The Parker Institute, Copenhagen University Hospital Bispebjerg and Frederiksberg, Copenhagen, Denmark; 6 Whitlam Orthopaedic Research Centre, Ingham Institute for Applied Medical Research, Sydney, New South Wales Australia; 7 Faculty of Medicine, South Western Sydney Clinical School, The University of New South Wales, Sydney, New South Wales Australia; 8 Institute of Musculoskeletal Health, School of Public Health, The University of Sydney, Sydney, New South Wales Australia

**Keywords:** Placebo response, Placebo effect, Contextual effects, Proportional contextual effect

## Abstract

**Background:**

Contextual effects (i.e., placebo *response*) refer to all health changes resulting from administering an apparently inactive treatment. In a randomized clinical trial (RCT), the overall treatment effect (i.e., the post-treatment effect in the intervention group) can be regarded as the true effect of the intervention plus the impact of contextual effects. This meta-research was conducted to examine the average proportion of the overall treatment effect attributable to contextual effects in RCTs across clinical conditions and treatments and explore whether it varies with trial contextual factors.

**Methods:**

Data was extracted from trials included in the main meta-analysis from the latest update of the Cochrane review on “*Placebo interventions for all clinical conditions”* (searched from 1966 to March 2008). Only RCTs reported in English having an experimental intervention group, a placebo comparator group, and a no-treatment control group were eligible.

**Results:**

In total, 186 trials (16,655 patients) were included. On average, 54% (0.54, 95%CI 0.46 to 0.64) of the overall treatment effect was attributable to contextual effects. The contextual effects were higher for trials with blinded outcome assessor and concealed allocation. The contextual effects appeared to increase proportional to the placebo effect, lower mean age, and proportion of females.

**Conclusion:**

Approximately half of the overall treatment effect in RCTs seems attributable to contextual effects rather than to the specific effect of treatments. As the study did not include all important contextual factors (e.g., patient-provider interaction), the true proportion of contextual effects could differ from the study’s results. However, contextual effects should be considered when assessing treatment effects in clinical practice.

**Trial registration:**

PROSPERO CRD42019130257. Registered on April 19, 2019.

**Supplementary Information:**

The online version contains supplementary material available at 10.1186/s13063-021-05454-8.

## Background

The importance of patients’ expectations, emotions, and clinical context in medical practice should not be ignored, but their impact on health care outcomes has only recently been evaluated [1–6]. Patients’ expectations and memories, the place in which the treatment is delivered, and the interaction between the patient and provider are just some of many factors in a “*therapeutic environment*” that can affect the treatment outcome [7, 8] and are linked to the placebo response [1, 7].

**Fig. 1:**
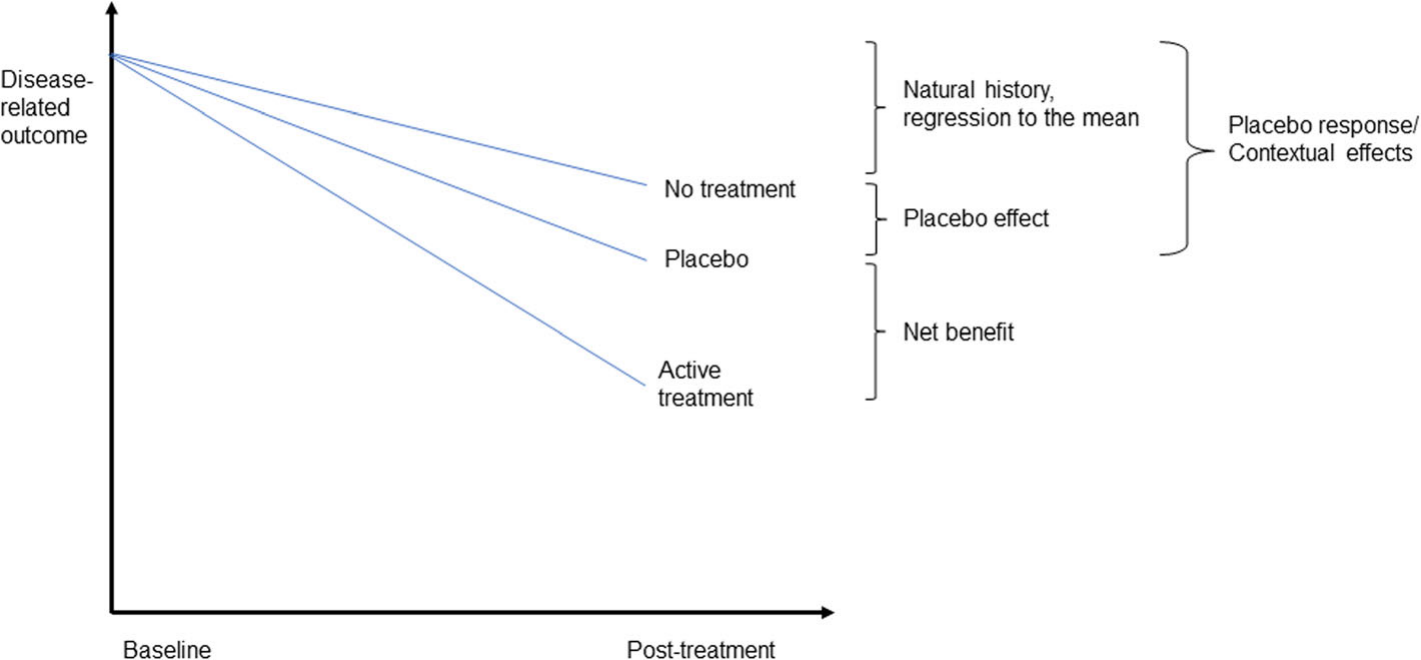
Explanatory diagram illustrating the contribution of the placebo effect and placebo response relative to the estimated effect of active treatment

The Society for Interdisciplinary Placebo Studies defines placebo *effects* as changes specifically attributable to placebo mechanisms (e.g., the neurobiological and psychological mechanisms of expectations), whereas placebo *response* refers to all health changes resulting from administering an inactive treatment, including regression towards the mean and natural course of the disease [9]. Hence, the placebo *response* includes the placebo *effect* and is also referred to as *contextual effects*; see Fig. 1.

Because any positive health change is of importance to patients, clinicians should acknowledge both the placebo *effect* (i.e. in research) and the placebo *response* (i.e. in practice). In clinical trials, the difference between the group receiving the experimental intervention and the placebo (comparator) group indicates the strength of the active treatment (i.e., net benefit). By looking only at the difference between these two groups, the clinical impact of the placebo response (i.e., the contextual effects) gets overlooked [10]. This omission can result in an ‘efficacy paradox’—a discrepancy between treatment effects reported in randomized clinical trials (RCTs), and the overall treatment effect experienced by patients and observed in clinical practice [11].

Contextual effects have been quantified by using the *proportional contextual effect* (PCE)—the proportion of the overall treatment effect attributable to contextual effects [12]. While the net benefit remains an important goal for any randomized trial, a shift in focus to the overall benefit and the PCE would mitigate the efficacy paradox and highlight the contribution of contextual effects, both in research and clinical practice.

Our primary objective of this meta-epidemiological study was to examine the average proportion of the overall treatment effect that may be explained as contextual effects, in an attempt to address the aforementioned “efficacy paradox” [10, 11]. Our secondary objective was to examine whether the contextual effects differ for different contextual factors; i.e., factors related to study design, type of intervention, and patients included. Our third objective was to explore the association between the contextual effects and placebo effect.

## Methods

Study selection, assessment of eligibility criteria, data extraction, and statistical analysis were performed based on a predefined protocol (PROSPERO registration no. CRD42019130257, Additional file 1), in accordance with the methodology guidelines from Cochrane. The findings are reported according to the Preferred Reporting Items for Systematic reviews and Meta-Analysis (PRISMA) statement [13] (Additional file 2).

### Data sources and searches

Only trials included in the latest update of the Cochrane review “*Placebo interventions for all clinical conditions*” by Hróbjartsson and Gøtzsche [14] were considered eligible, and therefore, no new literature search was performed [15, 16].

### Study selection

Only randomized trials having an experimental intervention group, a placebo comparator group, and a no-treatment (control) group were considered eligible. As in the original meta-analysis [14, 17], participants were patients with any somatic or psychiatric disease or symptoms. Besides the exclusion criteria described in detail in the Cochrane review [14], trials without an intervention group and trials written in languages other than English were excluded.

### Data collection process and data items

A data-extraction form was developed for data collection. One reviewer (S.H.H.) extracted data and selected the outcome of interest based on the description in the Cochrane review [14]. A second reviewer (R.C) was consulted when necessary, and doubts were discussed to consensus. The extracted data included the year of publication, study design, number of participants randomized and analyzed, baseline characteristics (average age, proportion of women, diagnosis, chronic or non-chronic condition), time of outcome measurement, type of placebo (pharmacological, physical, or psychological), experimental and no-treatment intervention and primary outcome.

Further, the type of outcome was categorized in (*i*) patient-reported outcomes (e.g., pain); (*ii*) observable patient-reported outcomes (e.g., vomiting); (*iii*) observer-reported outcomes dependent on cooperation of the patient (e.g., forced expiratory volume); (*iv*) observer-reported outcomes not dependent on patient cooperation (e.g., blood pressure); and (*v*) laboratory data (e.g., hemoglobin concentration). Other extracted information included dropout rate; blinding of participants, providers, and observers; allocation concealment; settings (i.e., single-center or multicenter); and information given to participants (i.e., whether participants were informed that the trial involved a placebo intervention). End-of-treatment data were preferred over follow-up data to reduce bias due to patients leaving the primary trial and effects consequentially diminishing. Change from baseline was preferred, but if only final values were available, these were used. For crossover trials, data were extracted from the first treatment period only, to avoid any carry-over effect. If that was not possible, summary data were used (naively) as if they had been derived from a parallel-group trial.

### Risk of bias in individual studies

Risk of bias of the included studies was assessed and compared to those from the original Cochrane review [14]. This approach enabled us to rate the risk of bias in domains from the Cochrane Risk of Bias tool [18] as *High*, *Low*, or *Unclear risk*. In addition, a simple risk-of-bias assessment proposed in the Cochrane review [14] was used, where trials with low risk of bias were defined as fulfilling the three following criteria: (*i*) adequate concealment of allocation, (*ii*) dropout rate no more than 15%, and (*iii*) inclusion of at least 50 patients.

### Summary measures

#### Proportional contextual effect (PCE)

For each trial, the PCE was calculated by dividing the improvement in the placebo control group (∆*m*_C_) by the improvement in the experimental intervention (∆*m*_I_) group (i.e., PCE=∆*m*_C_/∆*m*_I_) [19]. For trials with continuous outcomes, the improvement was defined as the mean change from baseline in the group, in the unit of standard deviation (SD). For trials with binary outcomes, improvement was defined as the number of participants improved in each group (placebo: *n*_C_, and intervention: *n*_I_), divided by the number of participants randomized to that particular group (*N*_C_ and *N*_I_). For trials with more than one relevant experimental intervention group (or placebo group), where all arms were relevant, treatment groups were combined into one group by calculating a weighted mean prior to perform the meta-analysis techniques. The PCE ratio was log_e_-transformed to normalize the distribution for the analysis and back-transformed for reporting. Theoretically, the PCE should range from 0 to 1, where 0 indicates no contribution from contextual effects while 1 indicates 100% contribution from contextual effects [10].

In trials with continuous outcomes, where either the intervention or placebo group showed no improvement (change score = 0) or worsening (negative score) from baseline, a miniscule effect (i.e., 1% benefit [multiplying by 1.01]) was imputed to enable estimation of the ratio (rather than excluding the trial from the primary analysis). In trials with binary outcomes, where no participants improved in the placebo group, it was not possible to log-transform the PCE. In these cases, we applied a “modified Woolf approach” where 0.5 was added to all cells as if 0.5 participant had improved and corresponding 0.5 participant had worsened, making it possible to include the trial in the primary quantitative synthesis.

#### Placebo effect in Cochrane review

For our third objective, exploring the association between the PCE and placebo effect, we used the estimates directly from the Cochrane review [14], odds ratio (OR) for binary outcomes, and standardized mean difference (SMD) for continuous outcomes—estimated by comparing the placebo comparator group and the no treatment control group. As a second step, in order to enable a meta-analytic combination of results, these placebo effect results were converted from OR to SMD. This conversion was done by converting the ln(OR) to the corresponding SMD, dividing by 1.81 (i.e.,$\pi /\sqrt{3}$), as suggested by Chinn [20].

### Data synthesis and analysis

A Restricted Maximum Likelihood (REML) mixed-effects model was applied to combine the log_e_(PCEs) across trials. To evaluate the degree of heterogeneity, the *I*^2^ index was estimated describing the percentage of total variation attributable to heterogeneity [21].

As outlined in the protocol, a number of pre-specified stratified analyses were performed: (*i*) time of outcome measurement, (*ii*) type of intervention, (*iii*) type of outcome, (*iv*) blinding of participants and treatment providers, (*v*) blinding of outcome assessor, (*vi*) allocation concealment, (*vii*) risk of bias, (*viii*) information to participants, and (*ix*) trial settings. Furthermore, the patient’s condition (i.e., chronic or non-chronic) was assessed at trial-level (based on the trial’s eligibility criteria).

Meta-regression analysis (REML models) was conducted involving covariates at trial-level to investigate whether individual covariates could explain heterogeneity (i.e., reduce variability) of the PCE among studies. These variables were (*i*) patient age, (*ii*) proportion of females, (*iii*) sample size, and (*iv*) year of publication. A meta-regression analysis was also performed to investigate the association between PCE and the corresponding placebo effect (i.e., SMD), as reported in the Cochrane review [14]. Small-study bias was examined using a funnel plot and Egger’s test [22]. Furthermore, based on the various risk-of-bias assessments for each trial, pre-specified exploratory sensitivity analyses were conducted in order to assess whether possible biases could affect the estimates. Trial characteristics, such as overall risk of bias, sample size [23], and trial settings (single-center or multicenter trial) [24], were further used in the pre-specified analyses to evaluate possible bias across studies. *P* values were obtained as part of the meta-regression analysis; i.e., based on *F* tests if more than two groups were compared and *t* tests if only two groups were compared. All statistical tests were performed using STATA/IC 15.1 (Stata Corp LLC, TX, USA).

## Results

### Search results

**Fig. 2:**
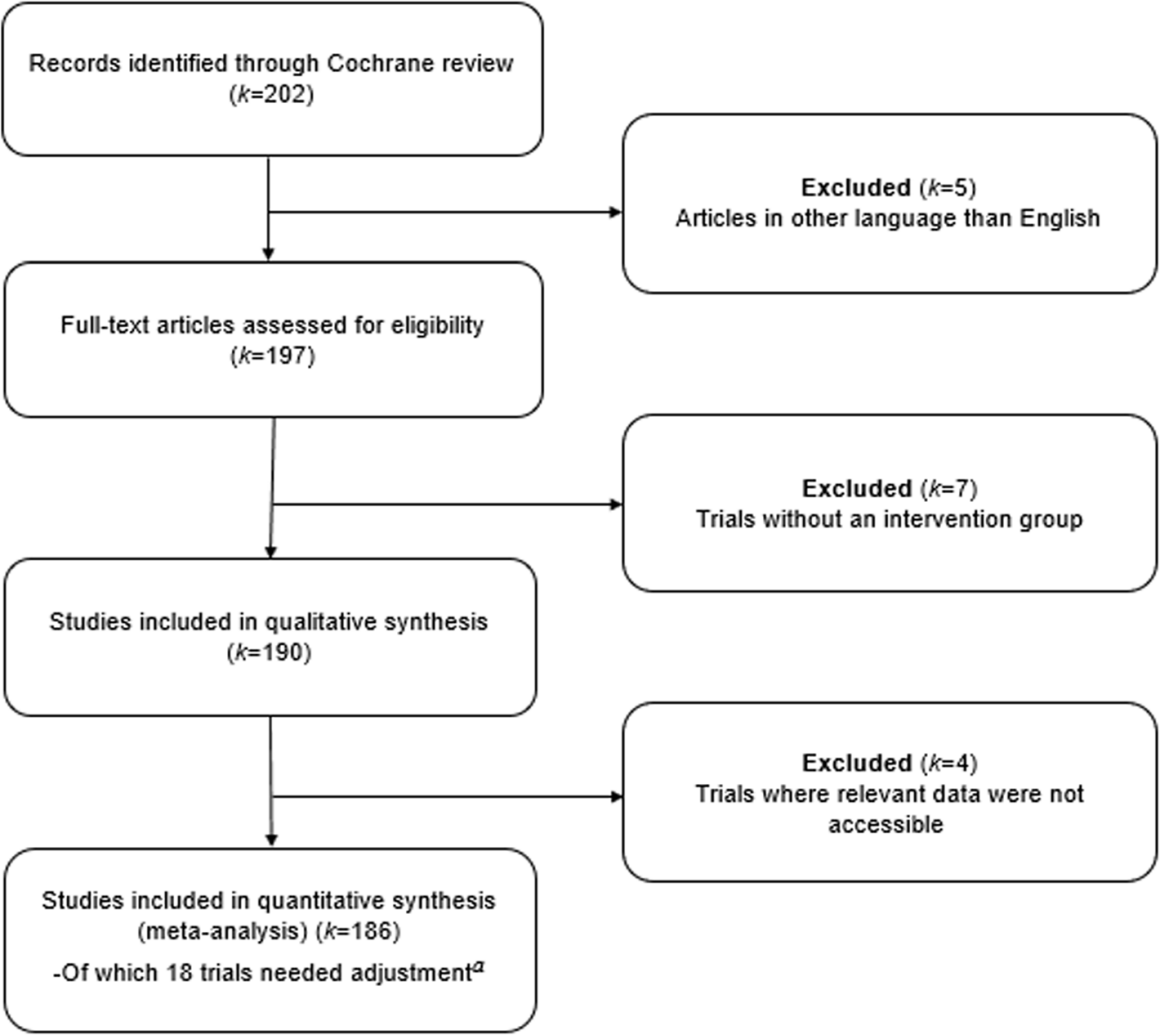
Flow diagram of study selection criteria. ^a^Trials where either intervention or placebo group showed no improvement or got worse, requiring adjustment in the form of imputation of a small treatment effect

All 202 trials included in the main analysis of the Cochrane review by Hróbjartsson and Gøtzsche [14] were screened for inclusion (Fig. 2). Five trials [25–29] were not in English. The remaining 197 trials were read in full text, of which 7 trials [30–36] did not meet the inclusion criteria. Relevant outcome data were not accessible in 4 trials [37–40], leaving 186 trials for inclusion in the meta-analysis.

### Included studies

The included trials were conducted in 23 countries and published between 1961 and 2008, with the majority published after 1994. There were 11 crossover trials [41–51], of which 10 (total of 250 patients) had no data available from the first period only, and thus were handled as parallel trials by using summary data (ignoring the design). There were 54 trials (total of 5,160 patients) having more than three arms. In 18 of those [52–69], one or more additional arms were disregarded (a total of 796 patients) in order to calculate the PCE for groups that had comparable interventions. In the remaining 36 trials with more than three arms, all arms were relevant and therefore included which left a total number of 16,655 patients for the meta-analysis.

Trial characteristics are summarized in Table 1. The mean age of participants ranged from 0 (infants) to 83.8 years, with an average mean age of 39.9 years (age was not reported in 11 trials). The percentage of females ranged from 0 to 100, with mean percentage of 58.6 (female/male proportions was not reported in 23 trials). Patients included in the trials had a broad spectrum of conditions, such as depression, hypertension, obesity, or headaches. Pain was the most common outcome measure (reported in 53 trials). Other frequent measures were for anxiety, medication use, smoking cessation, and nausea. Interventions were categorized into three types: pharmacological (e.g., medication or supplements given orally or via injection/inhalation), physical (e.g., acupuncture or transcutaneous electrical nerve stimulation), and psychological (e.g., cognitive therapy or hypnosis). In 22 trials, patients were not informed that the study involved placebo. There were 97 single-center trials and 33 multicenter trials (unclear in 56 studies). Explicit trial characteristics are presented in Additional file 3.

**Table 1:** Summary of trial characteristics

Characteristic	Trials (*k* = 186)
Number of patients	16,655
Proportion of women (SD)	58.6 (28.6)
Mean age, years (SD)	39.9 (17.1)
Condition	
Abortion, *n* (%)	3 (1.6)
Anxiety, *n* (%)	3 (1.6)
Asthma, *n* (%)	3 (1.6)
Cancer, *n* (%)	5 (2.9)
Dementia, *n* (%)	3 (1.6)
Depression, *n* (%)	9 (4.8)
Fibromyalgia, *n* (%)	3 (1.6)
Headache, *n* (%)	8 (4.3)
Hypertension, *n* (%)	7 (3.8)
Insomnia, *n* (%)	5 (2.9)
Low back pain, *n* (%)	3 (1.6)
Obesity, *n* (%)	8 (4.3)
Osteoarthritis, *n* (%)	5 (2.9)
Phobia, *n* (%)	3 (1.6)
Schizophrenia, *n* (%)	3 (1.6)
Smoking, *n* (%)	9 (4.8)
Surgery/procedure, *n* (%)	45 (24.2)
Other^a^, *n* (%)	61 (32.8)
No. center	
Single, *n* (%)	97 (52.1)
Unclear, *n* (%)	56 (30.1)
Multi, *n* (%)	33 (17.7)
Type of intervention	
Psychological, *n* (%)	62 (33.3)
Pharmacological, *n* (%)	56 (30.1)
Physical, *n* (%)	68 (36.6)
Study duration	
< 4 weeks, *n* (%)	83 (44.6)
4 to 8 weeks, *n* (%)	44 (23.7)
8 to 12 weeks, *n* (%)	27 (14.5)
> 12 weeks, *n* (%)	32 (17.2)
Outcome domain	
Anxiety, *n* (%)	9 (4.8)
Depression, *n* (%)	10 (5.4)
Diastolic blood pressure, *n* (%)	7 (3.8)
Medication use, *n* (%)	9 (4.8)
Nausea, *n* (%)	9 (4.8)
Pain, *n* (%)	53 (28.5)
Sleep disturbance, *n* (%)	6 (3.2)
Smoking cessation, *n* (%)	9 (4.8)
Weight, *n* (%)	8 (4.3)
Other^b^, *n* (%)	66 (35.5)
High risk, *n* (%)	170 (91.4)

^a^Conditions investigated in less than three trials, ^b^Outcomes reported in less than three trials

### Risk of bias within studies

Methodological characteristics were assessed by Hróbjartsson and Gøtzsche [14] and are listed in detail in the original Cochrane review. In summary, adequate sequence generation was reported in 39 trials (21%); 28 trials (15%) reported adequate concealment of allocation; 59 trials (32%) were judged to have adequate blinding of patients and providers; and 81 trials (44%) to have adequate blinding of outcome assessor. Overall, 81 trials (44%) had a sample size of at least 50 patients, and 80 trials (43%) had a dropout rate of 15% or lower. Following the simple risk-of-bias assessment proposed in the Cochrane review, 16 trials [70–85] (9%) fulfilled all three criteria and were therefore judged as having low risk of bias among the included trials.

### Proportional contextual effect

As illustrated in Additional file 4, there was a considerable degree of heterogeneity across PCE’s from various trials (*I*^2^=93.6). The pooled PCE across all 186 trials corresponded to 54% of the observed effect (0.54, 95%CI 0.46 to 0.64). There was statistically significantly higher PCE in trials with pharmacological and physical interventions, compared to psychological, and PCE was non-significantly higher for patient-involved outcomes (patient-reported outcomes and observer-reported outcomes dependent on patient cooperation). Trials where patients were not informed that the study involved placebo had non-significantly higher PCE. Trials with binary outcomes had statistically non-significant, but potentially higher PCE’s compared to trials with continuous outcomes. None of these factors, however, explained much of the observed heterogeneity. Publication year and sample size did not have any effect on heterogeneity either, and no association was found with PCE (see Additional file 5). There was a significant association between PCE and mean age and percentage of females, where PCE decreased with higher mean age of participants (slope = 0.986; 95%CI 0.976 to 0.995) and increased with higher percentage of females (slope = 1.006; 95%CI 1.000 to 1.012). However, only the proportion of females in the trial populations seemed to explain some of the between-study variance (reduction in *τ*^2^ = 14.1%, *I*^2^ = 92.5); see Table 2.

**Table 2:** Results from random-effect meta-analysis and meta-regression analysis

Study characteristic	Trials (***k***)	Patients (***n***)	PCE (95% CI)	***τ***^**2**^	***I***^**2**^	***P***
Overall (REML)	186	16,655	0.54 (0.46 to 0.64)	1.027	93.6	
Overall (REML, sensitivity analysis)	168	15,765	0.72 (0.67 to 0.79)	0.208	85.7	
Overall (D-L random)	186	16,655	0.59 (0.54 to 0.65)	0.253		
Overall (Fixed)	186	16,655	0.82 (0.80 to 0.83)	0.253		
Allocation concealment				1.005	93.6	0.024
Clearly concealed	28	4322	0.82 (0.55 to 1.21)			
Not clearly concealed	158	12,333	0.50 (0.41 to 0.59)			
Blinding of patients and providers				1.034	93.6	0.451
Clearly a double-blind design	59	6477	0.62 (0.46 to 0.84)			
Clearly not a double-blind design	97	8275	0.49 (0.39 to 0.62)			
Unclear	30	1903	0.55 (0.37 to 0.82)			
Blinding of outcome assessor				0.967	93.5	0.002
Clearly stated that outcome assessor was blinded	81	7614	0.72 (0.56 to 0.91)			
Not stated that outcome assessor was blinded	105	9041	0.43 (0.34 to 0.54)			
Low risk of bias				1.016	93.6	0.084
Clearly concealed allocation, dropout rate ≤15%, sample size > 49	16	3360	0.83 (0.50 to 1.40)			
Criteria not fulfilled	170	13,295	0.51 (0.43 to 0.61)			
Information to participants				1.029	93.6	0.477
Not informed that trial involved placebo	22	2150	0.64 (0.39 to 1.03)			
Informed that trial involved placebo or not stated	164	14,505	0.53 (0.44 to 0.63)			
Time of outcome measurement				1.029	93.6	0.578
< 4 weeks	83	6422	0.60 (0.47 to 0.76)			
4–8 weeks	44	2614	0.55 (0.38 to 0.78)			
> 8–12 weeks	27	2597	0.42 (0.27 to 0.65)			
> 12 weeks	32	5022	0.50 (0.34 to 0.75)			
Type of intervention				1.005	93.4	0.026
Pharmacological	56	6523	0.61 (0.45 to 0.82)			
Physical	68	6649	0.64 (0.50 to 0.83)			
Psychological	62	3483	0.38 (0.28 to 0.52)			
Type of outcome				1.029	93.7	0.523
Patient-reported outcomes that are observable	42	3605	0.59 (0.41 to 0.84)			
Patient-reported outcomes that are non-observable	88	7987	0.57 (0.45 to 0.72)			
Observer-reported outcomes dependent on patient cooperation	25	1143	0.53 (0.34 to 0.83)			
Observer-reported outcomes that were not dependent on patient cooperation	22	1314	0.42 (0.24 to 0.74)			
Laboratory outcomes	9	2606	0.31 (0.14 to 0.68)			
Settings				1.041	93.6	0.890
Single center	97	5268	0.54 (0.42 to 0.68)			
Multicenter	33	7394	0.51 (0.35 to 0.73)			
Unclear	56	3993	0.57 (0.42 to 0.78)			
Patient´s condition				0.996	93.4	0.028
Chronic condition	119	9771	0.47 (0.38 to 0.58)			
Non-chronic condition	67	6884	0.68 (0.52 to 0.88)			
Type of outcome				1.015	93.6	0.083
Binary outcome	39	5654	0.71 (0.50 to 1.01)			
Continuous outcome	147	11,001	0.50 (0.42 to 0.60)			
Sample size^a^				1.028	93.6	0.199
≤ 70 participants	93	2893	0.48 (0.37 to 0.61)			
≥ 71 participants	93	13,762	0.60 (0.48 to 0.74)			
Publication year				1.02	93.5	0.116
Published before 2000	120	8961	0.49 (0.39 to 0.60)			
Published in 2000 or later	66	7694	0.64 (0.49 to 0.83)			
Meta-regression of continuous variables	**Trials (*k*)**	**Patients (*n*)**	**Slope (95% CI)**	***τ***^**2**^	***I***^**2**^	***P***
Publication year	186	16,655	1.00 (0.98 to 1.02)	1.035	93.58	0.989
Sample size	186	16,655	1.00 (1.00 to 1.00)	1.026	93.54	0.162
Mean age	175	15,538	0.99 (0.98 to 0.99)	1.048	93.86	0.004
Percentage of females	163	15,259	1.01 (1.00 to 1.01)	0.8824	92.54	0.044
Placebo effect (SMD)	186	16,655	1.55 (1.07 to 2.24)	1.005	93.51	0.027

*k*, number of trials; *n*, number of patients analyzed; *τ*^2^, estimate of between-study variance; *I*^2^, variation in PCE attributable to heterogeneity, estimated by random-effect subgroup analysis

^a^Sample size analyzed by dividing the trials in two groups, 70.5 (the median) being the cut-point

Neither patient’s condition/diagnosis (e.g., fibromyalgia, cancer, smoking; increase in *τ*^2^ = 4.5%, *I*^2^ = 93.8) nor outcome domain (e.g., pain, nausea, smoking cessation; increase in *τ*^2^ = 0.7%, *I*^2^ = 93.5) was an important factor in reducing the between-study variance. However, whether the condition was chronic or not reduced between-study variance slightly (slope = 0.69; 95%CI 0.49 to 0.96; reduction in *τ*^2^ = 3.0%); chronic conditions had a significantly lower PCE.

### Proportional contextual effects and placebo effect

**Fig. 3:**
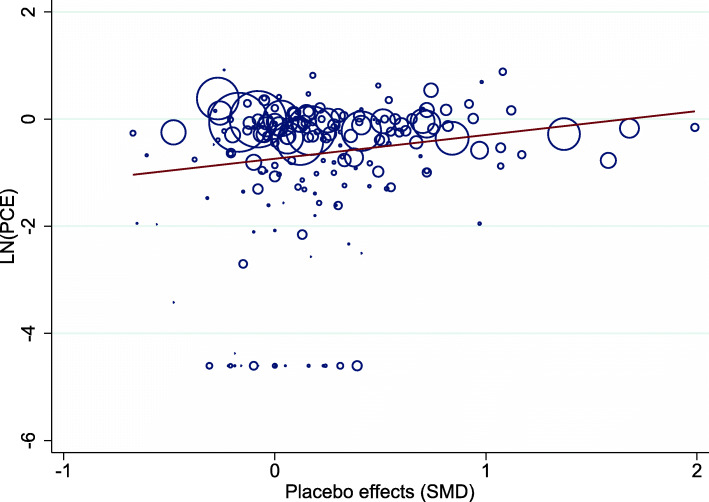
Meta-regression plot illustrating the association between the PCE and the placebo effect (SMD). Larger SMD indicates a larger placebo effect. (*k* = 186). PCE, proportional contextual effect; SMD, standardized mean difference

As illustrated in Fig. 3, there was an association between PCE and the corresponding placebo effect, estimated as SMD and reported in the Cochrane review [14], where PCE increased with an increasing placebo effect (slope = 1.55; 95%CI 1.07 to 2.24).

### Risk of bias across studies

**Fig. 4:**
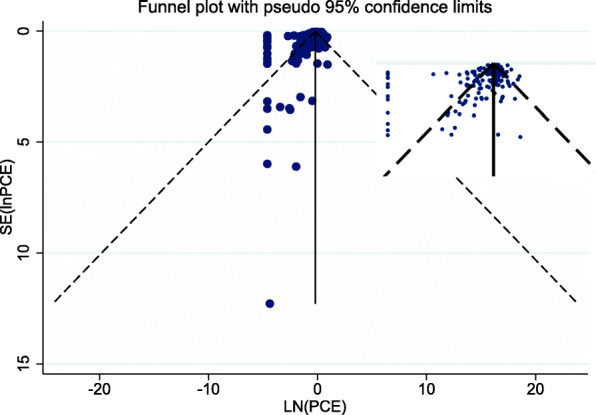
Funnel plot of all trials included in the main meta-analysis (*k* = 186). The vertical line shows the average effect size. The median SE(ln[PCE]) is 0.27; i.e., SE(ln[PCE]) larger than this are “smaller studies.” PCE, proportional contextual effect

The risk of small-study bias across trials was assessed using funnel plot, followed by Egger’s test [22]. The funnel plot was asymmetrical (Fig. 4), which Egger’s test confirmed (*p* < 0.001). Small studies tended to show smaller PCE. Furthermore, the funnel plot showed a vertical line of points (ln[PCE] = − 4.6) that indicated the trials where either the intervention group or the placebo group showed no improvement or got worse, requiring adjustment in the form of the described imputation of a small treatment effect. For sensitivity, when comparing the fixed-effect estimate for the PCE to the random-effects estimate, the PCE increased from 0.54 (95%CI 0.46 to 0.64) to 0.82 (95%CI 0.80 to 0.83). This indicates a discrepancy between random and fixed-effect models. However, a subsequent visual inspection of the funnel plot did not indicate an important small study bias.

When conducting the pre-specified sensitivity analyses according to the risk-of-bias assessment, the blinding of the outcome assessor was the most important factor, being associated with a significantly higher PCE. Furthermore, the PCE was significantly higher in trials with concealed allocation, and there was a non-significant increased PCE in trials with an overall low risk of bias. Neither trial setting (single-center or multicenter) nor sample size had an important effect on PCE or explained much of the reported heterogeneity, with blinding of outcome assessor apparently being the most important factor (reduction in *τ*^2^ = 5.8%).

What influenced the heterogeneity the most were the 18 trials [41, 46, 48, 86–100] (including 890 patients) where either the intervention or placebo group showed no improvement or got worse; these trials required imputing a very small treatment effect in order to calculate PCE so they could be included in the meta-analysis (reduction in *τ*^2^ = 80.6%, *I*^2^ = 84.4). Due to this imputation technique, a sensitivity analysis was performed where the aforementioned 18 trials were excluded.

### Sensitivity analysis

A meta-analysis of the remaining 168 studies (total of 15,765 patients) resulted in PCE of 0.72 (95%CI 0.67 to 0.79), *τ*^2^ = 0.2080, and a slightly lower heterogeneity (*I*^2^ = 85.7). Many of the trends apparent in the main analysis (e.g., higher PCE in trials with concealed allocation) diminished or disappeared (see Additional file 6). Ten of the 18 excluded trials had psychological interventions [87–90, 92, 93, 95, 97, 99, 100], which led to a radical shift in PCE for the remaining trials with psychological interventions. This shift in PCE, in turn, led to a non-significantly higher PCE in trials with physical interventions compared to pharmacological and psychological interventions. The PCE was still higher in trials with concealed allocation, blinded outcome assessor, and overall low risk of bias, but only the blinding of outcome assessor resulted in significantly higher PCE (slope = 1.22; 95%CI 1.03 to 1.44). The significantly higher PCE in trials of non-chronic conditions diminished and was no longer significant. None of these factors were important in explaining the between-study variance. Neither sample size, publication year, whether trials had binary or continuous outcomes, mean age of participants, nor percentage of females seemed to have any effect on heterogeneity, and no association was found with PCE in the sensitivity analyses.

**Fig. 5:**
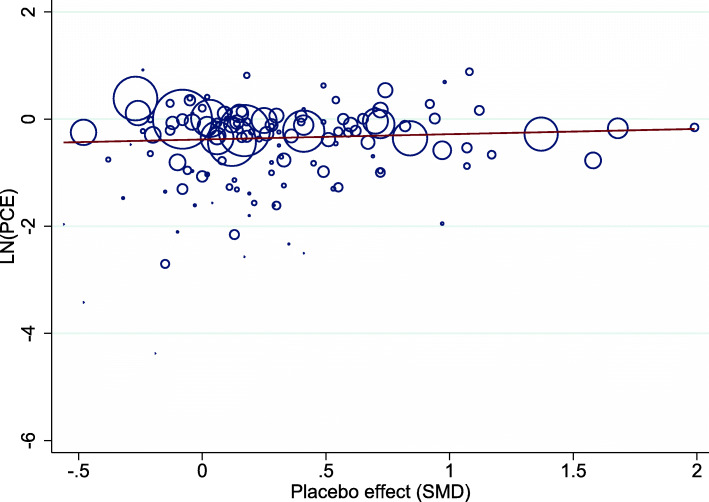
Meta-regression plot. Meta-regression plot, illustrating association between the PCE and the placebo effect (SMD) after exclusion of the 23 trials that needed adjustment prior to analysis. Larger SMD indicates a larger placebo effect (*k* = 163). PCE, proportional contextual effect

For the sensitivity analysis of the association between PCE and placebo effect, an additional five trials with binary outcomes were excluded because in these trials, no improvement was seen in the no-treatment group. In order to be eligible for inclusion in the synthesis, these trials needed adjustment (0.5 added to each cell of the 2 × 2 table) prior to calculating the OR, and afterwards SMD. The association found earlier between PCE and placebo effect diminished and was non-significant after the exclusion of all 23 trials that needed adjustment. The association between PCE and placebo effect (SMD) for the sensitivity analysis is illustrated in Fig. 5.

A new funnel plot and Egger’s test was conducted for the remaining 163 trials in the sensitivity analysis, which also resulted in an asymmetrical funnel plot (Additional file 7; Egger´s test, *p* < 0.001), suggesting that small studies report smaller PCE.

## Discussion

### Summary of the evidence

This study shows, based on 186 trials, that the majority (average PCE: 54%) of the overall treatment effect of diverse interventions across conditions was attributable to contextual effects. Several contextual factors and trial characteristics were found to impact the observed variation in PCE. The factors that increased the PCE, and thus are potentially valuable, were adequate allocation concealment, blinded outcome assessor, lower mean age of participants, higher proportion of females, larger placebo effect, and trials of non-chronic conditions. As anticipated the PCE was higher in trials with low risk of bias and using patient-reported outcomes. The proportion of females was the only pre-specified covariate that significantly reduced the between-study variance; other factors had only minimal impact on the between-study variance.

The sensitivity analysis showed insignificant variation of PCE for the most part; only blinding of outcome assessor remained significant. No factors were found to significantly reduce the between-study variance, and no association was found between PCE and mean age, percentage of females, or placebo effects, respectively.

Our findings support those reported in earlier PCE study by Zou et al. [12], who also found larger PCE in studies with concealed allocation. Neither study could explain, however, whether this is due to greater contextual effects or smaller experimental (intervention group) effects. Also, our findings support those found by Whiteside et al. [19], who reported an increase in PCE with higher proportions of females. There are, however, two crucial differences between this study and previous PCE studies. First, the two previous studies examined a single condition, (osteoarthritis [12] and fibromyalgia [19]), where the primary outcome was pain. Second, both studies excluded trials where one or more groups did not improve or even worsened. Due to these exclusions, it might be more appropriate to compare the findings from the sensitivity analysis in this study to the previous PCE studies. The overall PCEs reported in previous studies are similar to the findings from the present sensitivity analysis (75% in osteoarthritis and 60% in fibromyalgia compared to 72% in the present study). Zou et al. [12] reported the lowest PCE for treatments delivered with oral medications and higher PCE for treatments delivered via physical means and needles/injections. In the present sensitivity analysis, there was also insignificantly higher PCE in physical interventions compared to pharmacological. The categories in this study, however, cover a broader range of interventions than the categories used by Zou et al. [12]; nevertheless, our study generally corroborates earlier findings. We found that there was a direct association between PCE and the placebo effect reported in the Cochrane review [14]. This association, however, was not apparent in the sensitivity analysis, meaning that the PCE was constant even though the placebo effect diminished.

There are several similarities between the findings in the Cochrane review [14] and the findings in this study. The Cochrane review reported a larger placebo effect in trials with patient-reported outcomes, in trials where patients were falsely informed that no placebo was involved in the study, and in trials with concealed allocation. Similar results were seen in the sensitivity analysis in the present study, where trials with patient-reported outcomes suggested a higher PCE compared to outcomes not dependent on patient involvement. Trials with concealed allocation had significantly higher PCE in the main analysis but not in the sensitivity analysis. These similarities between PCE and placebo effect reported in the Cochrane review indicate that the difference between the improvements seen in the intervention and placebo groups is less prominent in studies with a large placebo effect.

### Limitations

A major limitation of this study was the inclusion of studies with high risk of bias, mainly due to lack of sequence generation and allocation concealment in 79% and 85% of trials, respectively. Only 16 trials (9%) out of 186 fulfilled all three predefined criteria for low risk of bias. An updated literature search may have added more studies at low risk of bias, as the majority of the included trials did not describe factors such as sequence generation and allocation concealment adequately. Since there were trends for higher PCE in trials with a low risk of bias this could have affected the outcomes. Furthermore, the meta-analytic methods used did not allow for patient-and clinical-related variables changes over time. Many changes in the patient populations could be experienced in 47 years.

This study correlates with previous studies of bias, reporting that bias due to unblinded assessor or inadequate allocation concealment tends to overestimate treatment effect [101–103]. Furthermore, a small-study bias was detected, whereby small-sample studies report smaller PCE. Based on the presence of small-study bias, and that studies with a lower risk of bias tended to have higher PCE, the PCE reported in this study could have been underestimated.

As described above, there was a considerably high degree of heterogeneity, which may have affected our study’s outcomes. Although subgroup analyses were carried out, the heterogeneity remained high, and no factors were able to explain the heterogeneity, except for the 18 trials that needed adjustment prior to analysis. Furthermore, not all the important contextual factors were included in the model. This could lead to uncontrolled confounding in the meta-analytic study, leading to biased effect estimates. Thus, the conclusions claimed in this article could be incorrect. A more in-depth sensitivity analysis would have been helpful to assess the magnitude of these biases. However, many contextual factors that are known to be important, such as the patient-provider interaction and patient’s expectations, were not considered because these factors are rarely reported in RCTs.

In contrast to earlier PCE studies, we decided to also include trials where either the intervention or placebo group showed no improvement or even worsened (i.e., where the PCE became negative). That decision might have been both a strength and a limitation. Earlier PCE studies excluded such trials because the measure of PCE does not allow negative values when the ratio is log-transformed, and worsening in a group could indicate side/nocebo effect, which is not the focus of interest for the PCE [12]. However, we viewed the exclusion of studies that did not fit to the pre-specified hypothesis as a source of bias; we therefore decided to add a miniscule effect to the groups that had not improved, in order to be able to perform the necessary calculations. This was the case for 17 trials with continuous outcomes and one trial with binary outcomes. Afterwards, it was clear these 18 trials had great impact on the results, and it was questionable whether this kind of adjustment was optimal for retaining the trials in our study.

## Conclusion

This study suggests that at least half of the overall treatment effect observed in clinical trials across conditions is attributable to contextual effects rather than to the specific experimental intervention on trial. Factors such as blinding of outcome assessor, concealed allocation, lower mean age, and higher proportion of females had the most impact on the PCE. This analysis in our study did not include all known important contextual factors (e.g., patient’s expectations), so the true proportion of contextual effects could have been underestimated. The association between PCE and methodological quality (lower risk of bias) indicates that the true PCE may be higher than estimated. These findings highlight the importance of contextual effects in clinical practice and their large impact on patient care. In reporting of RCTs, it is important to not only focus on the net benefit of a treatment but also to consider the PCE when translating findings from clinical trials to clinical practice. The findings could encourage health care professionals to consider potentially modifiable contextual factors, such as their patient-provider interaction, in any attempt to enhance the overall treatment response to interventions. Contextual effects are important contributors to the overall treatment effect and should be embraced in both clinical trials and practice for their potential benefits to patients.

## Supplementary Information

**Additional file 1.** Protocol.

**Additional file 2.** PRISMA Checklist.

**Additional file 3.** Characteristics of included trials. NS, not specified; ADHD, attention deficit hyperactivity disorder; OA, osteoarthritis; RA, rheumatoid arthritis. n, number of patients analyzed in intervention and placebo groups. ^a^Trials with low risk of bias fulfilled all three criteria: (i) clearly concealed allocation, (ii) dropout rate ≤15% and, (iii) sample size of at least 50. ^b^Patients analyzed in crossover trials that were handled as parallel-group trials (each patient counted twice).

**Additional file 4.** Forest plot. PCE, proportional contextual effect.

**Additional file 5.** Meta-regression plot, illustrating association between the PCE and publication year. PCE, proportional contextual effect.

**Additional file 6.** Results of sensitivity analysis (random-effect meta-analysis). k, number of trials; n, number of patients analyzed; τ^2^, estimate of between-study variance; I^2^, variation in PCE attributable to heterogeneity, estimated by random-effect subgroup analysis. ^a^Sample size analyzed by dividing the trials in two groups, 75 (the median) being the cut-point.

**Additional file 7.** Funnel plot of trials included in sensitivity analysis (k=163). The vertical line indicates the average effect size. PCE, proportional contextual effect.

**Additional file 8.** Dataset. Data on all screened trials.

## Data Availability

All data generated or analyzed during this study are included in this published article and its supplementary information files.

## Abbreviations

OR: Odds ratio
PCE: Proportional contextual effect
PRISMA: Preferred Reporting Items for Systematic reviews and Meta-Analyses RCT Randomized clinical trial REML Restricted Maximum Likelihood
SMD: Standardized mean difference 95% CI 95% confidence interval
