# Supplementary material for: Placebo response and effect in randomized clinical trials: meta-research with focus on contextual effects
Source: Trials. 2021 Jul 26;22:493. doi: 10.1186/s13063-021-05454-8 (PMC8314506; doi:10.1186/s13063-021-05454-8)
Supplement: Supplementary file 1 — Additional file 1. Protocol. [file 13063_2021_5454_MOESM1_ESM.docx]

**Placebo Response and Effect in Randomized Clinical Trials:**

**Protocol for a Meta-Analysis with Focus on Contextual Effects**

**Sigurlaug H. Hafliðadóttir, PT, BSc; Carsten B. Juhl, PT, BSc, MSc, PhD; Asbjørn Hróbjartsson, MD, PhD; Sabrina M. Nielsen, MSc; Marius Henriksen, PT, MSc, PhD; Weiya Zhang, BM, MPH, PhD; Ian A Harris, MD, MSc, PhD; Henning Bliddal, MD, DMSc; Robin Christensen, BSc, MSc, PhD**

**Date of Submission: 2019-04-19
PROSPERO: CRD42019130257**

**Collaborator/Author information:**

^SHH^ Department of Public Health, Faculty of Health Sciences, University of Southern Denmark, Odense & Musculoskeletal Statistics Unit, The Parker Institute, Bispebjerg and Frederiksberg Hospital, Copenhagen, Denmark. Email: [sihaf17@student.sdu.dk](mailto:sihaf17@student.sdu.dk)

^CBJ^ SEARCH Research Group, Research Unit of Musculoskeletal Function and Physiotherapy, Institute of Sports Science and Clinical Biomechanics, Faculty of Health Sciences, University of Southern Denmark, Odense, & Department of Physiotherapy and Occupational Therapy, University Hospital of Copenhagen, Herlev and Gentofte, Denmark. Email: [cjuhl@health.sdu.dk](mailto:cjuhl@health.sdu.dk)

^AH^ Centre for Evidence-Based Medicine Odense (CEBMO), Odense University Hospital, Odense & Department of Clinical Research, University of Southern Denmark, Odense & Odense Patient data Explorative Network (OPEN), Odense University Hospital, Odense, Denmark. Email: [asbjorn.hrobjartsson@rsyd.dk](mailto:asbjorn.hrobjartsson@rsyd.dk)

^SMN^ Musculoskeletal Statistics Unit, The Parker Institute, Bispebjerg and Frederiksberg Hospital, Copenhagen & Research Unit of Rheumatology, Department of Clinical Research, University of Southern Denmark, Odense University Hospital, Denmark. Email: [sabrina.mai.nielsen@regionh.dk](mailto:sabrina.mai.nielsen@regionh.dk)

^MH^ The Parker Institute, Copenhagen University Hospital Bispebjerg and Frederiksberg, Copenhagen & Department of Physical and Occupational Therapy, Copenhagen University Hospital Bispebjerg-Frederiksberg, Copenhagen, Denmark. Email: [marius.henriksen@regionh.dk](mailto:marius.henriksen@regionh.dk)

^WZ^ Arthritis Research UK Pain Centre, Nottingham & Division of Rheumatology, Orthopaedics, and Dermatology, School of Medicine, University of Nottingham, Nottingham & NIHR Nottingham Biomedical Research Centre, Nottingham University Hospitals, NHS Trust, Nottingham, United Kingdom. Email: [weiya.zhang@nottingham.ac.uk](mailto:weiya.zhang@nottingham.ac.uk)

^IH^ Whitlam Orthopaedic Research Centre, Ingham Institute for Applied Medical Research, Sydney & Faculty of Medicine, South Western Sydney Clinical School, The University of New South Wales, Sydney & Institute of Musculoskeletal Health, School of Public Health, The University of Sydney, Sydney, New South Wales, Australia. Email: [ianharris@unsw.edu.au](mailto:ianharris@unsw.edu.au)

^HB^ The Parker Institute, Bispebjerg and Frederiksberg Hospital, Copenhagen, Denmark. Email: [henning.bliddal@regionh.dk](mailto:henning.bliddal@regionh.dk)

^RC^ Musculoskeletal Statistics Unit, The Parker Institute, Bispebjerg and Frederiksberg Hospital, Copenhagen & Research Unit of Rheumatology, Department of Clinical Research, University of Southern Denmark, Odense University Hospital, Denmark. Email: [robin.christensen@regionh.dk](mailto:robin.christensen@regionh.dk)

**Corresponding Author:**

Robin Christensen, BSc, MSc, PhD; Biostatistician & Professor

Head of Musculoskeletal Statistics Unit, The Parker Institute, Bispebjerg and Frederiksberg Hospital, Denmark & Department of Rheumatology, Institute of Clinical Research, University of Southern Denmark, Odense University Hospital, Denmark

Mailing address: The Parker Institute, Bispebjerg and Frederiksberg Hospital, Nordre Fasanvej 57, DK-2000 Copenhagen F, Denmark.

e-mail: Robin.Christensen@regionh.dk/Tel: +45 3816 4165/Fax: +45 3816 4159

ORCID iD: 0000-0002-6600-0631/web: <http://www.parkerinst.dk/staff/robin-christensen>

**Disclaimers:**

The views expressed in the article are the authors own and not an official position of the institution or funder.

**Funding:**

The Parker Institute, Bispebjerg and Frederiksberg Hospital, is supported by a core grant from the Oak Foundation (OCAY-13-309).

# SUMMARY

## Background: In clinical practice, it is crucial to distinguish between *placebo effect* and *placebo response*. Placebo effect comprises the *changes specifically attributable* to placebo mechanisms, whereas placebo response (or contextual effects) refers to *all health changes* resulting from administering an inactive treatment. A Cochrane review on placebo effect reported that placebo interventions generally had no important clinical effects. Conversely, in clinical practice the placebo response may influence how patients feel and function.

## Objectives: This protocol applies three objectives: 1) to examine the proportion attributable to contextual effect (PCE) (i.e., the proportion of the overall treatment effect attributable to placebo response [contextual effect]), in randomized clinical trials (RCTs) of diverse treatments across clinical conditions; 2) to examine whether the PCE varies according to different contextual factors; and 3) to explore the association between the PCE and placebo effect.

## Data sources: Data will be extracted from trials included in the main meta-analysis from the latest update of the Cochrane review, “*Placebo interventions for all clinical conditions*” by Hróbjartsson & Gøtzsche.

**Study eligibility criteria (incl. participants and interventions):** Out of the trials from the original meta-analysis, only randomized trials having an experimental intervention group and a placebo group and a no-treatment group will be considered for inclusion. Participants will be patients with any somatic or psychiatric disease or symptoms. The eligible trial manuscripts must have a clearly labeled placebo (or sham) intervention in the trial report. The main outcome for each trial (i.e., PCE) will be extracted, and because the meta-analysis will include studies of diverse conditions, the types of outcome measures will vary. The PCEs will be combined using a Restricted Maximum Likelihood (REML) random-effects model; stratified analyses and meta-regression will be conducted to examine determinants of the PCE.

# INTRODUCTION

## Background

Icelanders have a saying: *Trúin flytur fjöll,* or “Faith moves mountains,” meaning that no matter how dark everything looks, faith that good things will happen can change everything. The importance of faith and hope is far from a new idea, but the effects of patient’ hopes and expectations in health care outcomes have only recently been evaluated (1-6). Patients’ expectations of treatment effect are just one among many factors within a patient‘s ‘therapeutic environment’ that can affect the treatment outcome (7, 8). Researchers typically have focused on patients‘s expectations when explaining the mechanisms of placebo response (9, 10). However, other factors in the therapeutic environment also have been linked to the placebo response. Such factors include patients’ memories and emotions, the place in which the treatment is delivered, and the interaction between patient and provider (1, 7).

Gaining a better understanding of the placebo phenomenon is further complicated because the meaning of placebo—as embodied in the terms *placebo effect and placebo response—*has changed radically over time since it first was described in medical use in the 18^th^ century as ‘a commonplace method or medicine’ (11). Indeed, a theoretical debate has emerged about the correct usage of these terms. According to the Oxford Advanced Learner’s Dictionary, the word *placebo* refers to “[a] *substance that has no physical effects, given to patients who do not need medicine but think that they do, or used when testing new drugs.*” Within medical research, however, no truly definitive standard exists for either placebo or placebo effect (12-14). Moreover, it has even been argued that placebo cannot be defined in a logical way (15). New terms, such as *contextual healing* (16) and *meaning response* (14) have been proposed in an attempt to reconceptualize the placebo phenomenon and move away from the term *placebo effect*. The ‘inert’ placebo itself does not elicit the effect; rather, the effect is elicited through the various factors, such as those mentioned above, or through natural history and regression to the mean. Generally, as also indicated by the Oxford Dictionary, in clinical trials, placebos are control treatments, appearing like the study treatments but without the ‘active’ components (17). The wording also tends to confuse because the terms *placebo effect* and *placebo response* have more or less been used interchangeably. The need for distinguishing between the two terms, however, has frequently been raised (12, 18-20). At the first official Society for Interdisciplinary Placebo Studies (SIPS) conference, the placebo effect was defined as including the changes specifically attributable to placebo mechanisms (for example, the neurobiological and psychological mechanisms of expectations), whereas placebo response, also referred to as contextual effects, refers to all health changes resulting from administering an inactive treatment, including regression to the mean and natural course of the disease (21).

Several studies have been conducted to quantify the placebo effect, and some have reported that placebo interventions can improve subjective and objective outcomes in up to 40 percent of patients within a wide range of clinical conditions (22, 23). These large effects have been questioned, however, because of methodological errors (12, 24) typically due to the risk of performance bias. A classic methodological error is to ascribe placebo effect to the before-after difference in a placebo group instead of using a proper control (i.e., no-treatment) group (12). By using a no-treatment group, it is possible to rule out the effects due to natural course of the disease and regression to the mean (12). In the Cochrane review on placebo effect, Hróbjartsson & Gøtzsche evaluated the empirical evidence for the effect associated with use of placebo treatments (17). Hróbjartsson & Gøtzsche argue that a placebo effect should be estimated as “…*an intervention, based on trials that randomize patients to a placebo intervention group and to a no-treatment control group”* (17). Using Hróbjartsson & Gøtzsche’s definition of placebo effect, the Cochrane review concluded that the placebo effect has no important clinical effect in placebo interventions in general. However, Hróbjartsson & Gøtzsche do indicate that placebo interventions could influence patient-reported outcomes in certain settings, especially in patients experiencing pain and nausea (17).

Using a no-treatment group is not without problems, however, because double-blinding is usually impossible, thereby inviting systematic difference in reporting, and other biases (12). Others point out that, logically, it is impossible to have a no-treatment group (25) because the participants will always ‘get more than nothing’; reading and signing informed consent and receiving some sort of examination and follow-up assessment are bound to have some effect. Essentially, even the no-treatment group will be influenced by the context of the trial. In order to investigate the improvements seen in no-treatment groups in RCTs, a study was conducted where the contribution of spontaneous improvement (i.e., natural course of the disease and regression to the mean), placebo effect, and effect of active interventions were measured (26). The study reported that on average the relative contributions of spontaneous improvement (i.e., the improvement of the no-treatment group) and of placebo to that of the active interventions were 24% and 20%, respectively, concluding that these factors contribute importantly to the observed treatment effect in actively treated patients (26).

## Rationale

Based on the aforementioned definition proposed at the SIPS conference, placebo response refers to all health changes resulting from inactive treatment, including spontaneous improvements. In clinical practice, these health changes are of great importance for the patient, hence, further investigation of the contribution of placebo response might be even more relevant than the conventional practice in clinical trials of separating treatment from placebo and placebo effect (difference between a blinded placebo intervention and a no-intervention group). In RCTs, the difference between the active treatment (experimental intervention) and placebo group (control comparator) indicates the strength of the active treatment (i.e., net benefit). By only looking at the difference between these two groups, the clinical impact of the placebo response (i.e., the contextual effect), gets overlooked (20), as illustrated in **Figure 1**. This omission can result in an ‘efficacy paradox’—a discrepancy between treatment effect that is reported in RCT or evidence-based guidelines, and what is observed in clinical practice (27). A treatment outcome is always delivered with contextual effects, and it is the overall effect of the treatment that matters to the patient, (19) regardless of the magnitude of the contribution from contextual effects.

The notion of contextual effects has been growing, and studies have been conducted in order to quantify them in RCTs by using the *proportional contextual effect* (PCE)—the proportion of the overall treatment effect attributable to contextual effect (19). Thus, pain reduction attributable to contextual effect in the treatment of osteoarthritis and fibromyalgia has been reported to be 75% (19) and 60% (28), respectively, indicating that contextual effects are a highly relevant research topic—also for clinical practice and even for those who organize health care systems. The net benefit of a specific treatment remains an important measure, but a shift in focus to the overall treatment effect and the proportional contextual effect would reduce the aforementioned efficacy paradox and highlight the contribution of contextual effects, both in RCTs and in clinical practice. Such a change in focus would be beneficial both for health professionals and the people they treat.

| 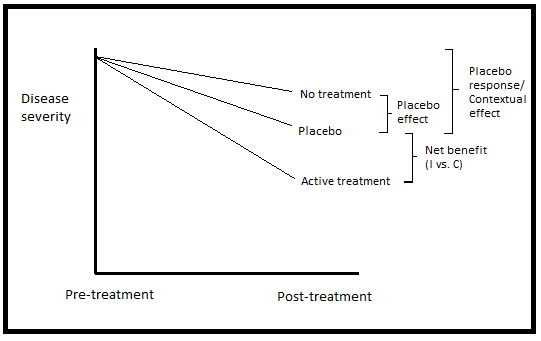 |
| --- |
| **Figure 1.** Visual illustration of contribution of placebo effect (ε) and placebo response (ρ) to the estimated effect of active treatment (net benefit: θ) |

## Aim and Objectives

The aim of this systematic review is to assess the contextual effects in RCTs of diverse treatments across clinical conditions by re-analyzing trials from a previous meta-analysis of placebo effect (17, 29).

Our primary objective is to examine the proportion of the overall treatment effect that may be explained by a contextual effect, in an attempt to overcome the aforementioned ‘efficacy paradox’ (20, 27). Our secondary objective is to examine whether the contextual effect differs for various types of factors related to study design, type of intervention, and condition recruited. Our third objective is to explore the association between the PCE and placebo effect.

**Hypothesis**

We hypothesize that the majority of the overall treatment effect will be attributable to contextual effects (i.e., that the lower part of the 95% confidence interval will be larger than 50%).

# METHODS

## Protocol and registration

Our protocol is registered on PROSPERO (CRD42019130257); our protocol manuscript follows the Preferred Reporting Items for Systematic Reviews and Meta-Analysis Protocols (PRISMA-P) guidelines for reporting systematic reviews and meta-analysis protocols (30).

## Eligibility criteria

Out of the trials from the original meta-analysis (17, 29), only randomized trials having an intervention group, placebo group, and no-treatment group will be considered for inclusion. Both parallel and crossover studies will be considered eligible. End-of-treatment data will be preferred instead of follow-up data to reduce bias due to patients leaving the trial and effects consequentially diminishing.

As in the original meta-analysis (17, 29), participants will be patients with any somatic or psychiatric diseases or symptoms. The interventions included must have a clearly labelled placebo intervention in the trial report. A placebo intervention can be labelled using the word *placebo* or an analogous term, such as *sham, fake*, or *dummy treatment*.

The main outcome for each trial will be extracted, and because the meta-analysis will include studies of diverse conditions, the types of outcomes will vary. The outcomes can include patient-reported outcomes, both private to the patient and potentially observable by another person; and observer-reported outcomes, including outcomes depending on patient cooperation, outcomes not depending on patient cooperation, and laboratory data.

Beside the exclusion criteria described in detail in the Cochrane review (17), trials without an intervention group and trials written in a language other than English will be excluded.

**Information sources**

Only trials included in the main meta-analysis in the latest update of the Cochrane review “Placebo interventions for all clinical conditions” by Hróbjartsson & Gøtzsche (17) will be considered for potential eligibility.

**Study selection**

The full reports of studies included in the Cochrane review from Hróbjartsson & Gøtzsche (17) will be read in full by SHH, who will decide whether the reports meet the inclusion criteria (without looking at the outcome data). The reason for exclusion will be recorded. A second author will be consulted if necessary (RC).

**Data collection process**

Information will be extracted from the trial reports using a pilot-tested standardized data chart. SHH will select the outcome of interest based on the description in the Cochrane review by Hróbjartsson & Gøtzsche (17); any disagreements over this selection will be resolved through discussion (RC). The abstracted data will include the trial methods, participants, and intervention details. End-of-treatment data will be preferred to follow-up data. For crossover trials, we will extract data from the first treatment period only, due to the risk of carry-over effect. If that is not possible, we will use the summary data as if they had been derived from a parallel-group trial.

**Data items**

For the eligible RCTs, we will extract information on author, year of publication, journal of publication, registration number, trial sponsors, and date of protocol published. We will extract the objectives of the trial, inclusion and exclusion criteria, date of recruitment, total number of participants randomized and analyzed, number of participants in each group, and baseline and eligibility characteristics (average age, number of women, diagnosis, whether it is an acute or chronic condition). We will also extract time of outcome measurement, study design, the type of placebo used (pharmacological, physical or psychological), interventions used, assignment to no-treatment group, and primary outcome. Further, we will extract type of outcome, using the following categories:

1. Patient-reported outcome that is private to the patient (e.g., pain)
2. Patient-reported outcome that is potentially observable (e.g., vomiting)
3. Observer-reported outcome dependent on cooperation of the patient (e.g., measurement of forced expiratory volume)
4. Observer-reported outcome that is not dependent on patient cooperation (e.g., blood pressure)
5. Laboratory data (e.g., hemoglobin concentration)

Lastly, we will extract whether the trial is single-center or multicenter; dropout rate; blinding of participants, providers, and observers; allocation concealment; and information to participants.

**Risk of bias in individual studies**

The risk of bias assessment from the latest update of the Cochrane review (17) will be used.

**Summary measures**

***Proportional contextual effect - PCE***

For each trial, the PCE will be calculated by dividing the improvement in the placebo group by the improvement in the treatment group (28). The improvement in the intervention group will indicate the overall treatment effect. For trials with binary outcomes, the improvement will be extracted and defined as the number of participants improved in the group, divided by the total number of participants in that group. For trials with continuous outcomes, the improvement will be defined as the mean change from baseline in the group, in the unit of its standard deviation (SD). For trials with more than one active treatment, where all treatment arms are relevant, treatment groups will be combined to one composite group, where the improvement for that group will be defined as the mean improvement with a variance that accounts for the correlation among the different treatments (31). Where the additional treatment arms are not relevant, they will not be taken into account. The PCE will be log-transformed to normalize the distribution and will be a ratio, ranging from 0 to 1, where 0 indicates no contribution from contextual effects, whereas 1 indicates 100% contribution from contextual effects (20). For trials where either the intervention or placebo group shows no improvement (change score = 0) or worsening (negative score) from baseline, a miniscule effect (i.e., 1 percent) of the improvement of the will be imputed to permit log-transformation. The PCE will be presented with a 95% confidence interval (CI).

***Placebo effect in Cochrane review***

Our third objective, to explore the association between the placebo effect and PCE, will use the Cochrane review by Hróbjartsson & Gøtzsche (17) and the placebo effect reported therein. The Cochrane review includes trials with binary and continuous outcomes, and a separate meta-analysis will be conducted on each type of outcome. Initially, this analysis will estimate the Odds Ratio (OR) for binary outcomes and standardized mean difference (SMD) for continuous outcomes. As a second step, in order to investigate the association between PCE and placebo effect, results from trials in the Cochrane review having binary outcomes will be converted from OR to SMD. This will be done by i) computing binary outcome measures to OR, and ii) converting ln(OR) to the corresponding SMD by dividing by 1.81 (i.e., $\frac{\pi}{\sqrt{3}}$ ) as suggested by Chinn (32).

**Synthesis of results**

Due to the expected heterogeneity, a Restricted Maximum Likelihood (REML) random-effects model will be applied for the meta-analysis. All statistical tests will be performed using STATA/IC 15.1 (Stata Corp LLC, Texas, USA). All results will be reported with 95% confidence intervals (CIs). To estimate the degree of heterogeneity, an I^2^ test will be performed, which describes the percentage of total variation across studies that is caused by heterogeneity rather than by chance (33).

**Pre-specified exploratory analysis**

A number of pre-specified stratified analyses will be performed according to trial characteristics.

The trial characteristics will include:

1. Time of outcome measurement
   1. less than 4 weeks
   2. 4 to 8 weeks
   3. 8 to 12 weeks
   4. more than 12 weeks
2. Type of intervention
   1. Pharmacological
   2. Physical
   3. Psychological
3. Type of outcome
   1. Patient-reported outcomes that are non-observable
   2. Patient-reported outcomes that are observable
   3. Observer-reported outcomes dependent on the cooperation of the patient
   4. Observer-reported outcomes that are not dependent on patient cooperation
   5. Laboratory outcomes
4. Blinding of participants and treatment providers
   1. Clearly a double-blind design
   2. Clearly not double blind
   3. Unclear
5. Blinding of outcome assessor
   1. Clearly stated that outcome assessor was blinded
   2. Not stated that outcome assessor was blinded
6. Allocation concealment
   1. The randomization and allocation of participants was clearly concealed
   2. The randomization and allocation of participants was not clearly concealed
7. Risk of bias
   1. Clearly concealed allocation of participants, and dropout rate of 15% or lower, and sample size of at least 50 participants
   2. Above criteria not fulfilled
8. Information to participants
   1. Participants were not informed that the trial involved a placebo intervention
   2. Participants were informed that the trial involved placebo intervention
   3. Unclear
9. Settings
   1. Single-center trial
   2. Multicenter trial
   3. Unclear

Furthermore, a REML meta-regression analysis will be conducted involving continuous variables at trial-level, to investigate whether particular covariates can explain any of the heterogeneity (i.e., reduce variability) of the PCE among studies.

These variables will be:

1. Patient age in years
2. Patient sex as percentage of females
3. Sample size
4. Year of publication

A meta-regression analysis will also be performed to further investigate the association between PCE and the corresponding placebo effect (i.e., SMD) reported in the Cochrane review by Hróbjartsson & Gøtzsche (17).

**Risk of bias across studies**

Publication bias will be examined using a funnel plot and Egger’s test (34). We will evaluate whether selective reporting of outcomes is present by assessing whether the protocol for the study was published before recruitment of study participants. Where no protocol was available, outcomes reported in the Methods and Results sections will be compared. For studies published after July 1, 2005, we will screen the Clinical Trial Register at the International Clinical Trials Registry Platform of the World Health Organization. We will evaluate whether selective reporting of outcomes is present (outcome-reporting bias). We will compare the fixed-effect estimate against the random-effects model to assess the possible presence of small-sample bias in the published literature (i.e., in which the intervention effect is more beneficial in smaller studies, resulting in a smaller relative placebo response). In the presence of small-sample bias, the random-effects estimate of the intervention is more beneficial than is the fixed-effect estimate.

**Tables and figures**

| **Figure 1.** **Flow diagram.** |
| --- |
| 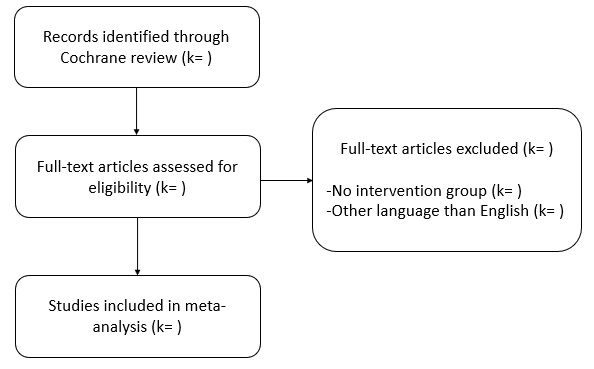 |

**Table 1.** **Characteristics of included studies**

| Author+ year | Pop. (n) | Dropout (%) | Condition | Type of inter-vention | Duration of inter-vention | Outcome | Women (%) | Mean age (SD) | Risk of bias (3 criteria*) | Center no. |
| --- | --- | --- | --- | --- | --- | --- | --- | --- | --- | --- |
|  |  |  |  |  |  |  |  |  |  |  |
|  |  |  |  |  |  |  |  |  |  |  |

*For low risk of bias, all three critera must be fulfilled: i) clearly concealed allocation, ii) dropout rate ≤15% iii), sample size of at least 50.

**Table 2.** **Results of the stratified meta-analysis**

| Variable |  | No. of trials | No. of participants | Relative placebo response (95% CI) | I^2^ % of relative placebo response |
| --- | --- | --- | --- | --- | --- |
| Overall |  |  |  |  |  |
| Time of outcome measurement |  |  |  |  |  |
|  | <4 weeks |  |  |  |  |
|  | 4-8 weeks |  |  |  |  |
|  | 8-12 weeks |  |  |  |  |
|  | >13 weeks |  |  |  |  |
| Type of intervention |  |  |  |  |  |
|  | Pharmacological |  |  |  |  |
|  | Physical |  |  |  |  |
|  | Psychological |  |  |  |  |
| Type of outcome |  |  |  |  |  |
|  | Patient-reported outcomes that are observable |  |  |  |  |
|  | Patient-reported outcomes that are non-observable |  |  |  |  |
|  | Observer-reported outcomes dependent on the cooperation of the patient |  |  |  |  |
|  | Observer-reported outcomes that are not dependent on patient cooperation |  |  |  |  |
|  | Laboratory outcomes |  |  |  |  |
| Blinding of participants and treatment providers |  |  |  |  |  |
|  | Clearly a “double- blind” design |  |  |  |  |
|  | Clearly not a “double- blind” design |  |  |  |  |
|  | Unclear |  |  |  |  |
| Blinding of outcome assessor |  |  |  |  |  |
|  | Clearly stated that outcome assessor was blinded |  |  |  |  |
|  | Not stated that outcome assessor was blinded |  |  |  |  |
| Allocation concealment |  |  |  |  |  |
|  | Clearly concealed |  |  |  |  |
|  | Not clearly concealed |  |  |  |  |
| Risk of bias |  |  |  |  |  |
|  | Clearly concealed allocation, dropout rate <15%, sample size >50 |  |  |  |  |
|  | Criteria not fulfilled |  |  |  |  |
| Information to participants |  |  |  |  |  |
|  | Not informed that trial involved a placebo |  |  |  |  |
|  | Informed that trial involved placebo |  |  |  |  |
|  | Unclear |  |  |  |  |
| Settings |  |  |  |  |  |
|  | Single center |  |  |  |  |
|  | Multicenter |  |  |  |  |
|  | Unclear |  |  |  |  |

**Figure 2A:** Forrest plot illustrating overall analysis of PCE

**Figure 2B:** Forrest plot illustrating overall analysis of placebo effect (SMD).

| **Figure 3:** Graph illustrating the association between PCE and placebo effect (i.e., SMD).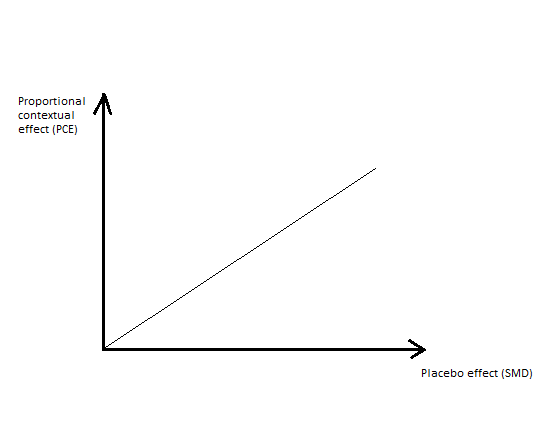 |
| --- |
| *A sketch of Figure 3.* |

# REFERENCES

1. Di Blasi Z, Harkness E, Ernst E, Georgiou A, Kleijnen J**.** Influence of context effects on health outcomes: a systematic review. Lancet. 2001;357(9258):757-62.

2. Kaptchuk TJ, Friedlander E, Kelley JM, Sanchez MN, Kokkotou E, Singer JP, et al. Placebos without deception: a randomized controlled trial in irritable bowel syndrome. PLoS One. 2010;5(12):e15591.

3. Atlas LY, Wager TD**.** How expectations shape pain. Neurosci Lett. 2012;520(2):140-8.

4. Colloca Lab, Miller FGb**.** Role of expectations in health. Curr Opin Psychiatry. 2011;24(2):149-55.

5. Amanzio M, Benedetti F**.** Neuropharmacological dissection of placebo analgesia: expectation-activated opioid systems versus conditioning-activated specific subsystems. J Neurosci. 1999;19(1):484-94.

6. Benedetti F, Pollo A, Lopiano L, Lanotte M, Vighetti S, Rainero I**.** Conscious expectation and unconscious conditioning in analgesic, motor, and hormonal placebo/nocebo responses. J Neurosci. 2003;23(10):4315-23.

7. Rossettini G, Carlino E, Testa M**.** Clinical relevance of contextual factors as triggers of placebo and nocebo effects in musculoskeletal pain. BMC Musculoskelet Disord. 2018;19(1):27.

8. Carlino E, Benedetti F**.** Different contexts, different pains, different experiences. Neuroscience. 2016;338:19-26.

9. Kirsch I**.** Response expectancy as a determinant of experience and behavior. Am Psychol. 1985;40(11):1189-202.

10. Kirsch I**.** Response expectancy and the placebo effect. In: Colloca L, ed. Int Rev Neurobiol: Academic Press; 2018:81-93.

11. de Craen AJ, Kaptchuk TJ, Tijssen JG, Kleijnen J**.** Placebos and placebo effects in medicine: historical overview. J R Soc Med. 1999;92(10):511-5.

12. Hróbjartsson A**.** What are the main methodological problems in the estimation of placebo effects? J Clin Epidemiol. 2002;55(5):430-5.

13. Turner A**.** What are the benefits of a new placebo language? Perspect Biol Med. 2018;61(3):401-11.

14. Moerman DE, Jonas WB**.** Deconstructing the placebo effect and finding the meaning response. Ann Intern Med. 2002;136(6):471-6.

15. Gøtzsche PC**.** Is there logic in the placebo? Lancet. 1994;344(8927):925-6.

16. Miller FG, Kaptchuk TJ**.** The power of context: reconceptualizing the placebo effect. J R Soc Med. 2008;101(5):222-5.

17. Hróbjartsson A, Gøtzsche PC**.** Placebo interventions for all clinical conditions. Cochrane Database Syst Rev. 2010(1):CD003974.

18. Hróbjartsson A**.** The uncontrollable placebo effect. Eur J Clin Pharmacol. 1996;50(5):345-8.

19. Zou K, Wong J, Abdullah N, Chen X, Smith T, Doherty M, et al. Examination of overall treatment effect and the proportion attributable to contextual effect in osteoarthritis: meta-analysis of randomised controlled trials. Ann Rheum Dis. 2016;75(11):1964-70.

20. Zhang W, Doherty M**.** Efficacy paradox and proportional contextual effect (PCE). Clin Immunol. 2018;186:82-6.

21. Evers AWM, Colloca L, Blease C, Annoni M, Atlas LY, Benedetti F, et al. Implications of placebo and nocebo effects for clinical practice: expert consensus. Psychother Psychosom. 2018;87(4):204-10.

22. Beecher HK**.** The powerful placebo. J Am Med Assoc. 1955;159(17):1602-6.

23. Lasagna L**.** The placebo effect. J Allergy Clin Immunol. 1986;78(1 Pt 2):161-5.

24. Kienle GS, Kiene H**.** The powerful placebo effect: fact or fiction? J Clin Epidemiol. 1997;50(12):1311-8.

25. Moerman DE**.** Meaning, medicine and the 'placebo effect'. Cambridge, United Kingdom: Cambridge University Press; 2002.

26. Krogsbøll LT, Hróbjartsson A, Gøtzsche PC**.** Spontaneous improvement in randomised clinical trials: meta-analysis of three-armed trials comparing no treatment, placebo and active intervention. BMC Med Res Methodol. 2009;9:1.

27. Walach H**.** The efficacy paradox in randomized controlled trials of CAM and elsewhere: beware of the placebo trap. J Altern Complement Med. 2001;7(3):213-8.

28. Whiteside N, Sarmanova A, Chen X, Zou K, Abdullah N, Doherty M, et al. Proportion of contextual effects in the treatment of fibromyalgia-a meta-analysis of randomised controlled trials. Clin Rheumatol. 2018;37(5):1375-82.

29. Hróbjartsson A, Gøtzsche PC**.** Is the placebo powerless? N Engl J Med. 2001;344(21):1594-602.

30. Shamseer L, Moher D, Clarke M, Ghersi D, Liberati A, Petticrew M, et al. Preferred reporting items for systematic review and meta-analysis protocols (PRISMA-P) 2015: elaboration and explanation. BMJ 2015;349:g7647.

31. Borenstein M, Hedges L, Higgins J, Rothstein H**.** Introduction to meta-analysis: United States: John Wiley & Sons Inc; 2009.

32. Chinn S**.** A simple method for converting an odds ratio to effect size for use in meta-analysis. Stat Med. 2000;19(22):3127-31.

33. Higgins JP, Thompson SG, Deeks JJ, Altman DG**.** Measuring inconsistency in meta-analyses. BMJ 2003;327(7414):557-60.

34. Egger M, Davey Smith G, Schneider M, Minder C**.** Bias in meta-analysis detected by a simple, graphical test. BMJ 1997;315(7109):629-34.
