# Supplementary figures and images for: Placebo response and effect in randomized clinical trials: meta-research with focus on contextual effects
Source: Trials. 2021 Jul 26;22:493. doi: 10.1186/s13063-021-05454-8 (PMC8314506; doi:10.1186/s13063-021-05454-8)

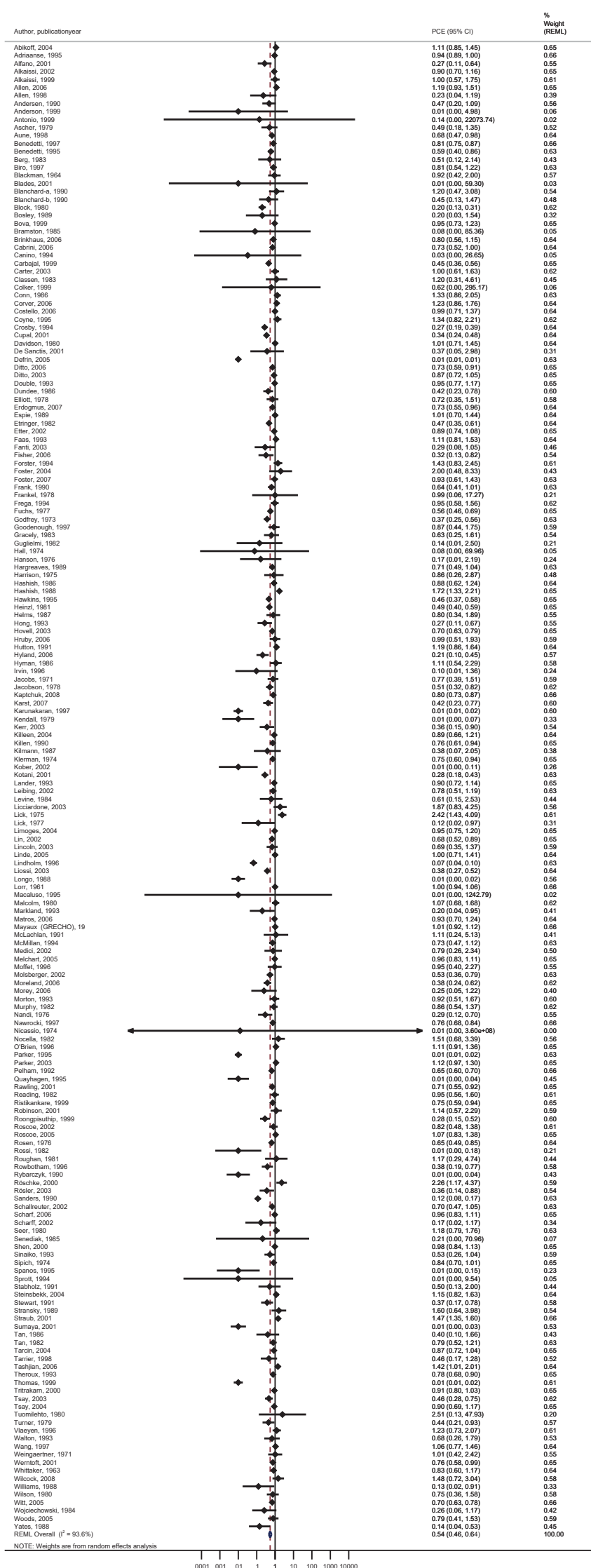

Supplement: Supplementary file 4 — Additional file 4. Forest plot. PCE, proportional contextual effect. [file 13063_2021_5454_MOESM4_ESM.pdf]

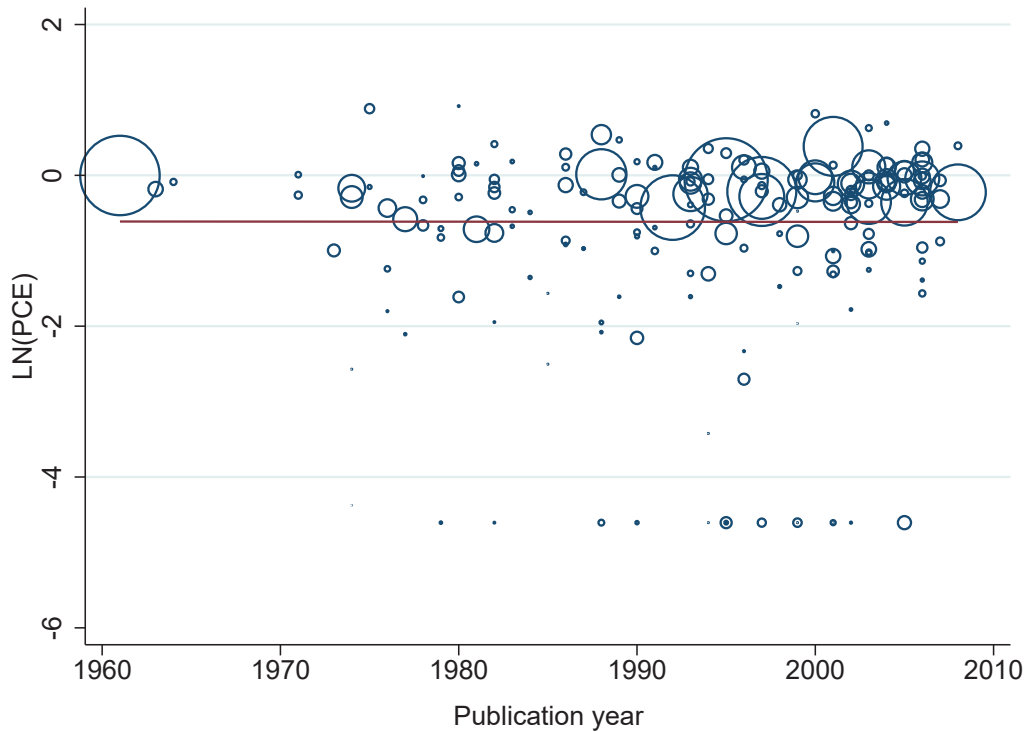

Supplement: Supplementary file 5 — Additional file 5. Meta-regression plot, illustrating association between the PCE and publication year. PCE, proportional contextual effect. [file 13063_2021_5454_MOESM5_ESM.pdf]

Funnel plot with pseudo 95% confidence limits

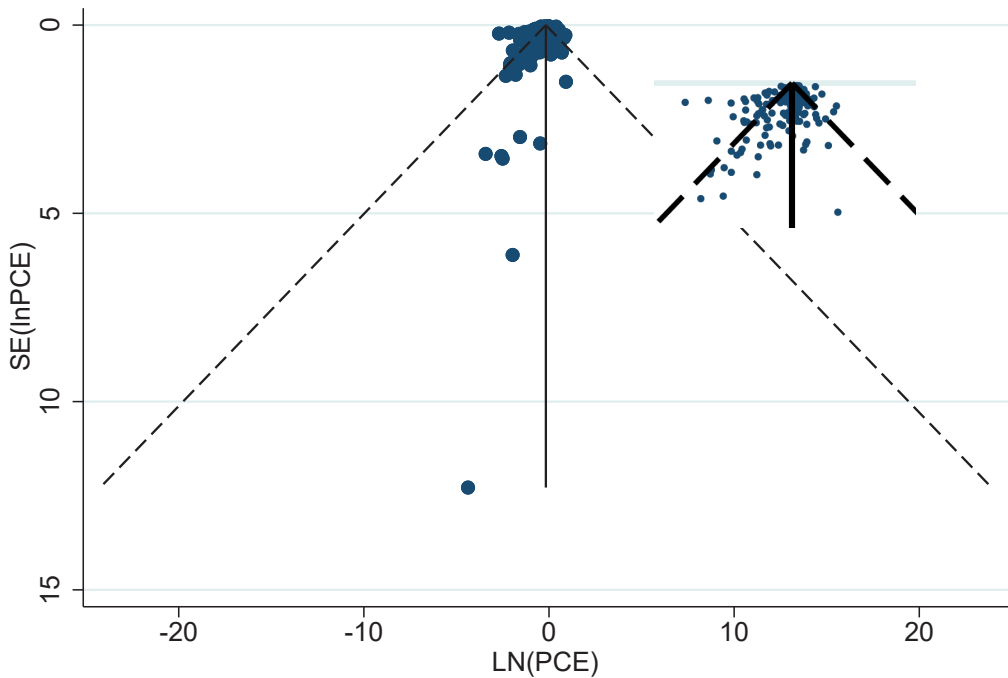

Supplement: Supplementary file 7 — Additional file 7. Funnel plot of trials included in sensitivity analysis (k=163). The vertical line indicates the average effect size. PCE, proportional contextual effect. [file 13063_2021_5454_MOESM7_ESM.pdf]
