# Supplementary material for: Placebo response and effect in randomized clinical trials: meta-research with focus on contextual effects
Source: Trials. 2021 Jul 26;22:493. doi: 10.1186/s13063-021-05454-8 (PMC8314506; doi:10.1186/s13063-021-05454-8)
Supplement: Supplementary file 6 — Additional file 6. Results of sensitivity analysis (random-effect meta-analysis). k, number of trials; n, number of patients analyzed; τ2, estimate of between-study variance; I2, variation in PCE attributable to heterogeneity, estimated by random-effect subgroup analysis. aSample size analyzed by dividing the trials in two groups, 75 (the median) being the cut-point. [file 13063_2021_5454_MOESM6_ESM.docx]

| **Additional file 6 – Results of sensitivity analyses (random-effect meta-analysis)** | | | | | | |  |  |  |
| --- | --- | --- | --- | --- | --- | --- | --- | --- | --- |
| Study Characteristic |  | **Trials (k)** | | **Patients (n)** | | **PCE (95% CI)** | **τ^2^** | **I^2^** | **P** |
| Overall (REML, sensitivity analysis) | | 168 | | | 15,765 | 0.72 (0.67 to 0.79) | 0.208 | 85.7 |  |
| Allocation concealment |  |  | |  | |  | 0.208 | 85.7 | 0.136 |
|  | Clearly concealed | 28 | | 4,322 | | 0.82 (0.68 to 1.00) |  |  |  |
|  | Not clearly concealed | 140 | | 11,443 | | 0.70 (0.64 to 0.77) |  |  |  |
| Blinding of patients and providers |  |  | |  | |  | 0.211 | 85.5 | 0.848 |
|  | Clearly a double-blind design | 54 | | 6,114 | | 0.74 (0.64 to 0.86) |  |  |  |
|  | Clearly not a double-blind design | 86 | | 7,780 | | 0.73 (0.64 to 0.82) |  |  |  |
|  | Unclear | 28 | | 1,871 | | 0.69 (0.56 to 0.84) |  |  |  |
| Blinding of outcome assessor |  |  | |  | |  | 0.200 | 85.6 | 0.019 |
|  | Clearly stated that outcome assessor was blinded | 76 | | 7,422 | | 0.80 (0.71 to 0.91) |  |  |  |
|  | Not stated that outcome assessor was blinded | 92 | | 8,343 | | 0.66 (0.59 to 0.74) |  |  |  |
| Low risk of bias |  |  | |  | |  | 0.207 | 85.8 | 0.170 |
|  | Clearly concealed allocation, dropout rate ≤15%, sample size >49 | 16 | | 3,360 | | 0.85 (0.67 to 1.08) |  |  |  |
|  | Criteria not fulfilled | 152 | | 12,405 | | 0.71 (0.65 to 0.78) |  |  |  |
| Information to participants |  |  | |  | |  | 0.209 | 85.5 | 0.328 |
|  | Not informed that trial involved placebo | 20 | | 2,043 | | 0.81 (0.64 to 1.03) |  |  |  |
|  | Informed that trial involved placebo or not stated | 148 | | 13,722 | | 0.71 (0.65 to 0.78) |  |  |  |
| Time of outcome measurement |  | |  |  | |  | 0.212 | 85.8 | 0.797 |
|  | <4 weeks | 75 | | 6,124 | | 0.75 (0.66 to 0.84) |  |  |  |
|  | 4-8 weeks | 41 | | 2,454 | | 0.69 (0.57 to 0.83) |  |  |  |
|  | >8-12 weeks | 24 | | 2,415 | | 0.75 (0.59 to 0.95) |  |  |  |
|  | >12 weeks | 28 | | 4,772 | | 0.68 (0.55 to 0.83) |  |  |  |
| Type of intervention |  |  | |  | |  | 0.206 | 85.2 | 0.224 |
|  | Pharmacological | 52 | | 6,213 | | 0.69 (0.59 to 0.80) |  |  |  |
|  | Physical | 64 | | 6,516 | | 0.79 (0.69 to 0.90) |  |  |  |
|  | Psychological | 52 | | 3,036 | | 0.67 (0.57 to 0.79) |  |  |  |
| Type of outcome |  |  | |  | |  | 0.200 | 85.9 | 0.064 |
|  | Patient-reported outcomes that are observable | 38 | | 3,468 | | 0.76 (0.64 to 0.91) |  |  |  |
|  | Patient-reported outcomes that are non-observable | 80 | | 7,571 | | 0.76 (0.68 to 0.85) |  |  |  |
|  | Observer-reported outcomes dependent on patient cooperation | 23 | | 1,070 | | 0.75 (0.59 to 0.94) |  |  |  |
|  | Observer-reported outcomes that were not dependent on patient cooperation | 20 | | 1,288 | | 0.52 (0.38 to 0.70) |  |  |  |
|  | Laboratory outcomes | 7 | | 2,368 | | 0.51 (0.34 to 0.76) |  |  |  |
| Settings |  |  | |  | |  | 0.212 | 85.5 | 0.896 |
|  | Single center | 87 | | 4,844 | | 0.72 (0.64 to 0.81) |  |  |  |
|  | Multicenter | 30 | | 7,183 | | 0.75 (0.63 to 0.90) |  |  |  |
|  | Unclear | 51 | | 3,738 | | 0.71 (0.60 to 0.83) |  |  |  |
| Patient’s condition |  |  | |  | |  | 0.208 | 85.3 | 0.154 |
|  | Chronic condition | 105 | | 9,094 | | 0.68 (0.61 to 0.77) |  |  |  |
|  | Non-chronic condition | 63 | | 6,671 | | 0.78 (0.68 to 0.88) |  |  |  |
| Type of outcome |  |  | |  | |  | 0.209 | 85.7 | 0.463 |
|  | Binary outcome | 38 | | 5,628 | | 0.77 (0.64 to 0.92) |  |  |  |
|  | Continuous outcome | 130 | | 10,137 | | 0.71 (0.65 to 0.78) |  |  |  |
| Sample size^a^ |  |  | |  | |  | 0.207 | 85.7 | 0.200 |
|  | ≤74 participants | 83 | | 2,582 | | 0.68 (0.59 to 0.77) |  |  |  |
|  | ≥75 participants | 85 | | 13,183 | | 0.76 (0.68 to 0.85) |  |  |  |
| Publication year |  |  | |  | |  | 0.206 | 85.5 | 0.143 |
|  | Published before 2000 | 106 | | 8.264 | | 0.69 (0.61 to 0.77) |  |  |  |
|  | Published in 2000 or later | 62 | | 7,501 | | 0.78 (0.68 to 0.89) |  |  |  |

________________________________________________________________________________
