# Supplementary material for: Placebo response and effect in randomized clinical trials: meta-research with focus on contextual effects
Source: Trials. 2021 Jul 26;22:493. doi: 10.1186/s13063-021-05454-8 (PMC8314506; doi:10.1186/s13063-021-05454-8)
Supplement: Supplementary file 8 — Additional file 8. Dataset. Data on all screened trials. [file 13063_2021_5454_MOESM8_ESM.docx]

**Dataset**

**Additional file 8**

Data on all screened trials. ID number corresponds to reference number.

|  | | | | | | | | | | | | | | | | Binary outcomes | | | | | | Continuous outcomes | | | | | | | | | | Risk of bias | | | | | | | |
| --- | --- | --- | --- | --- | --- | --- | --- | --- | --- | --- | --- | --- | --- | --- | --- | --- | --- | --- | --- | --- | --- | --- | --- | --- | --- | --- | --- | --- | --- | --- | --- | --- | --- | --- | --- | --- | --- | --- | --- |
| **ID** | **Reason of exclusion** | **Setting** | **Study design** | **Number of arms** | **Type of intervention** | **Duration** | **Participants analyzed (N)** | **Int+pl. Analyzed (n)** | **Age (mean) (weighted)** | **Women (%) weighted** | **Chronic** | **Type of outcome** | **Outcome (cluster)** | **No information to participants** | **Binary/Continuous outcome** | **Intervention, events (n)** | **Intervention, total (N)** | **Placebo, events (n)** | **Placebo, total (N)** | **No-treatm., events (n)** | **No-treatm., total (N)** | **Intervention (N)** | **Baseline, intervention (mean)** | **Baseline, intervention (SD)** | **End, intervention (mean)** | **End, intervention (SD)** | **Placebo (N)** | **Baseline, placebo (mean)** | **Baseline, placebo (SD)** | **End, placebo (Mean)** | **End, placebo (SD)** | **Adequate sequence generation** | **Concealed allocation adequate** | **Clearly double-blindet** | **Observer clearly blinded** | **Low dropout rate** | **Protocol published** | **Reasonable sample size** | **Low risk of bias** |
| 1 | 0 | 3 | 1 | 3 | 2 | 4 | 103 | 69 | 8,45 |  | 1 | 1 | 13 | 0 | 1 |  |  |  |  |  |  | 34 | 75,7 | 20,4 | 88,3 | 20,1 | 35 | 75,7 | 22,4 | 91,4 | 18,9 | 0 | 0 | 0 | 0 | 0 | 0 | 1 | 0 |
| 2 | 0 | 3 | 1 | 3 | 0 | 4 | 981 | 655 | 30,17 | 100 | 0 | 5 | 13 | 0 | 0 | 33 | 327 | 50 | 328 | 38 | 326 |  |  |  |  |  |  |  |  |  |  | 0 | 0 | 1 | 0 | 0 | 0 | 1 | 0 |
| 3 | 0 | 1 | 1 | 5 | 1 | 4 | 94 | 80 | 45,4 | 93 | 1 | 2 | 3 | 0 | 1 |  |  |  |  |  |  | 56 | 7,06 | 2,35 | 5,5 | 2,45 | 24 | 6,7 | 2,3 | 6,2 | 2,8 | 1 | 0 | 2 | 0 | 0 | 0 | 0 | 0 |
| 4 | 0 | 2 | 1 | 3 | 1 | 1 | 60 | 40 | 30,33 | 100 | 0 | 1 | 8 | 0 | 0 | 9 | 20 | 9 | 20 | 11 | 20 |  |  |  |  |  |  |  |  |  |  | 0 | 0 | 0 | 1 | 0 | 0 | 0 | 0 |
| 5 | 0 | 3 | 1 | 3 | 1 | 1 | 410 | 274 |  | 100 | 0 | 1 | 8 | 0 | 0 | 68 | 135 | 77 | 139 | 82 | 136 |  |  |  |  |  |  |  |  |  |  | 0 | 1 | 0 | 1 | 1 | 0 | 1 | 1 |
| 6 | 0 | 2 | 1 | 3 | 1 | 2 | 34 | 23 | 31,5 | 100 | 1 | 3 | 9 | 0 | 1 |  |  |  |  |  |  | 12 |  |  |  |  | 11 |  |  |  |  | 0 | 0 | 1 | 1 | 1 | 0 | 0 | 0 |
| 7 | 0 | 2 | 1 | 3 | 1 | 2 | 131 | 87 | 41,18 | 68,7 | 1 | 3 | 9 | 1 | 1 |  |  |  |  |  |  | 42 | 22,1 | 4,8 | 13,42 | 9,6 | 45 | 22 | 5 | 12 | 9,6 | 0 | 0 | 1 | 1 | 1 | 0 | 1 | 0 |
| 8 | 0 | 2 | 1 | 3 | 0 | 1 | 52 | 36 | 27,73 | 100 | 0 | 2 | 2 | 0 | 1 |  |  |  |  |  |  | 18 |  |  |  |  | 18 |  |  |  |  | 0 | 0 | 1 | 1 | 0 | 0 | 0 | 0 |
| 9 | 0 | 1 | 1 | 3 | 2 | 4 | 85 | 58 | 12,64 | 50 | 1 | 1 | 13 | 0 | 1 |  |  |  |  |  |  | 28 | 2,68 | 0,9 | 2,71 | 0,6 | 30 | 2,7 | 0,84 | 2,67 | 0,84 | 0 | 0 | 0 | 0 | 1 | 0 | 1 | 0 |
| 10 | 1 |  |  |  |  |  |  |  |  |  |  |  |  |  | 1 |  |  |  |  |  |  |  |  |  |  |  |  |  |  |  |  |  |  |  |  |  |  |  |  |
| 11 | 0 | 1 | 1 | 3 | 0 | 2 | 18 | 12 | 47,7 | 55,56 | 1 | 4 | 6 | 0 | 1 |  |  |  |  |  |  | 6 | 80,9 | 14,7 | 78,3 | 14,6 | 6 | 80 | 11,8 | 79,7 | 12,2 | 0 | 0 | 1 | 0 | 1 | 0 | 0 | 0 |
| 12 | 0 | 2 | 1 | 3 | 2 | 2 | 25 | 16 | 39 | 67 | 1 | 1 | 5 | 0 | 1 |  |  |  |  |  |  | 8 | 61,5 | 35,72 | 28,6 | 16,6 | 8 | 63,25 | 37,42 | 50,63 | 44,13 | 0 | 0 | 0 | 0 | 0 | 0 | 0 | 0 |
| 13 | 1 |  |  |  |  |  |  |  |  |  |  |  |  |  | 1 |  |  |  |  |  |  |  |  |  |  |  |  |  |  |  |  |  |  |  |  |  |  |  |  |
| 14 | 0 | 2 | 1 | 3 | 1 | 4 | 67 | 53 | 34,8 | 100 | 1 | 1 | 13 | 0 | 0 | 4 | 27 | 11 | 26 | 9 | 14 |  |  |  |  |  |  |  |  |  |  | 1 | 1 | 0 | 1 | 1 | 0 | 0 | 0 |
| 15 | 0 | 2 | 1 | 8 | 0 | 1 | 33 | 22 | 55 | 79 | 0 | 2 | 2 | 0 | 1 |  |  |  |  |  |  | 9 |  |  |  |  | 13 |  |  |  |  | 0 | 0 | 1 | 1 | 0 | 0 | 0 | 0 |
| 16 | 0 | 2 | 1 | 3 | 1 | 1 | 324 | 209 |  |  | 0 | 2 | 2 | 0 | 1 |  |  |  |  |  |  | 103 |  |  |  |  | 106 |  |  |  |  | 0 | 0 | 2 | 0 | 0 | 0 | 1 | 0 |
| 17 | 0 | 1 | 1 | 3 | 0 | 4 | 40 | 25 | 7,9 |  | 1 | 1 | 13 | 0 | 0 | 9 | 14 | 9 | 11 | 9 | 15 |  |  |  |  |  |  |  |  |  |  | 0 | 0 | 1 | 1 | 1 | 0 | 0 | 0 |
| 18 | 0 | 2 | 1 | 5 | 0 | 1 | 86 | 57 |  |  | 0 | 2 | 2 | 0 | 1 |  |  |  |  |  |  | 28 |  |  |  |  | 29 |  |  |  |  | 0 | 0 | 1 | 1 | 1 | 0 | 1 | 0 |
| 19 | 0 | 1 | 1 | 3 | 0 | 1 | 35 | 23 | 21 | 0 | 1 | 1 | 13 | 0 | 0 | 5 | 11 | 6 | 12 | 6 | 12 |  |  |  |  |  |  |  |  |  |  | 0 | 0 | 1 | 1 | 1 | 0 | 0 | 0 |
| 20 | 0 | 1 | 2 | 3 | 0 | 2 | 40 | 80 | 53,4 | 75 | 1 | 5 | 13 | 0 | 1 |  |  |  |  |  |  | 40 | 16,1 | 6,84 | 16,3 | 6,5 | 40 | 16,1 | 6,84 | 16 | 5,96 | 0 | 0 | 1 | 0 | 0 | 0 | 1 | 0 |
| 21 | 0 | 2 | 1 | 4 | 2 | 4 | 55 | 44 | 39,4 | 62,1 | 1 | 2 | 4 | 0 | 1 |  |  |  |  |  |  | 31 | 27,4 | 41,1 | 15,1 | 24,7 | 13 | 12,1 | 11 | 8,3 | 13,6 | 0 | 0 | 0 | 0 | 1 | 0 | 0 | 0 |
| 22 | 0 | 2 | 1 | 4 | 2 | 4 | 83 | 59 | 38,35 | 78,26 | 1 | 2 | 4 | 0 | 1 |  |  |  |  |  |  | 41 | 18,54 | 24,02 | 9,85 | 18,36 | 18 | 16,3 | 28,9 | 11,9 | 23,9 | 0 | 0 | 0 | 0 | 0 | 0 | 0 | 0 |
| 23 | 0 | 2 | 1 | 3 | 2 | 3 | 40 | 32 | 37,7 | 70 | 1 | 4 | 6 | 0 | 1 |  |  |  |  |  |  | 16 | 24,8 | 4,6 | 15,5 | 4,6 | 16 | 25,3 | 5 | 22,4 | 6,6 | 0 | 0 | 0 | 0 | 0 | 0 | 0 | 0 |
| 24 | 0 | 2 | 1 | 3 | 2 | 2 | 41 | 27 | 57 | 0 | 1 | 4 | 10 | 0 | 1 |  |  |  |  |  |  | 14 | 89 | 10 | 84 | 10 | 13 | 87 | 10 | 86 | 10 | 0 | 0 | 0 | 1 | 0 | 0 | 0 | 0 |
| 25 | 0 | 2 | 1 | 3 | 0 | 1 | 110 | 74 | 55 | 62,7 | 0 | 2 | 2 | 0 | 1 |  |  |  |  |  |  | 40 |  |  |  |  | 34 |  |  |  |  | 0 | 0 | 2 | 0 | 1 | 0 | 1 | 0 |
| 26 | 0 | 2 | 1 | 4 | 2 | 2 | 48 | 36 | 28,06 |  | 1 | 3 | 13 | 0 | 1 |  |  |  |  |  |  | 24 | 73,29 | 11 | 77,3 | 9,72 | 12 | 77,58 | 13,3 | 78 | 14,21 | 0 | 0 | 0 | 1 | 1 | 0 | 0 | 0 |
| 27 | 0 | 3 | 1 | 3 | 1 | 2 | 284 | 210 | 58,8 | 67,8 | 1 | 2 | 3 | 1 | 1 |  |  |  |  |  |  | 140 |  |  |  |  | 70 |  |  |  |  | 1 | 1 | 0 | 1 | 1 | 1 | 1 | 1 |
| 28 | 0 | 1 | 1 | 3 | 1 | 1 | 48 | 32 | 61 | 60,4 | 0 | 2 | 13 | 0 | 1 |  |  |  |  |  |  | 16 |  |  |  |  | 16 |  |  |  |  | 0 | 0 | 0 | 1 | 0 | 0 | 0 | 0 |
| 29 | 0 | 1 | 1 | 3 | 2 | 3 | 21 | 12 | 35 | 33,3 | 1 | 4 | 10 | 0 | 1 |  |  |  |  |  |  | 8 | 96 | 4,9 | 83,8 | 2,5 | 4 | 99,8 | 7,9 | 99 | 2,7 | 0 | 0 | 0 | 1 | 0 | 0 | 0 | 0 |
| 30 | 0 | 1 | 1 | 6 | 0 | 1 | 150 | 125 | 0 | 44,67 | 0 | 3 | 2 | 0 | 1 |  |  |  |  |  |  | 100 |  |  |  |  | 25 |  |  |  |  | 1 | 1 | 2 | 0 | 0 | 0 | 1 | 0 |
| 31 | 0 | 2 | 1 | 3 | 2 | 2 | 85 | 56 | 27 | 100 | 1 | 1 | 13 | 1 | 0 | 13 | 28 | 13 | 28 | 20 | 29 |  |  |  |  |  |  |  |  |  |  | 1 | 1 | 0 | 0 | 0 | 0 | 1 | 0 |
| 32 | 2 |  |  |  |  |  |  |  |  |  |  |  |  |  | 1 |  |  |  |  |  |  |  |  |  |  |  |  |  |  |  |  |  |  |  |  |  |  |  |  |
| 33 | 0 | 2 | 2 | 2 | 0 | 1 | 45 | 30 | 42,9 | 60 | 1 | 2 | 3 | 0 | 1 |  |  |  |  |  |  | 15 | 15,7 | 7,2 | 14,4 | 7,2 | 15 | 13,1 | 8,3 | 11,3 | 8,3 | 0 | 0 | 1 | 1 | 0 | 0 | 0 | 0 |
| 34 | 0 | 1 | 1 | 3 | 0 | 2 | 20 | 16 | 43,05 |  | 1 | 4 | 6 | 0 | 1 |  |  |  |  |  |  | 9 | 90,9 | 17,5 | 89,5 | 16 | 7 | 83,6 | 17,5 | 82,7 | 18 | 0 | 0 | 1 | 0 | 1 | 0 | 0 | 0 |
| 35 | 0 | 1 | 1 | 3 | 1 | 1 | 42 | 28 | 23,05 | 53 | 0 | 2 | 2 | 0 | 1 |  |  |  |  |  |  | 15 |  |  |  |  | 13 |  |  |  |  | 0 | 1 | 2 | 0 | 0 | 0 | 0 | 0 |
| 36 | 0 | 2 | 1 | 3 | 1 | 4 | 1019 | 603 | 4 | 47,45 | 1 | 1 | 13 | 0 | 0 | 275 | 324 | 227 | 279 | 360 | 416 |  |  |  |  |  |  |  |  |  |  | 0 | 0 | 1 | 1 | 1 | 0 | 1 | 0 |
| 37 | 0 | 1 | 1 | 3 | 0 | 1 | 127 | 85 | 13 | 54 | 0 | 2 | 2 | 0 | 1 |  |  |  |  |  |  | 37 |  |  |  |  | 48 |  |  |  |  | 1 | 0 | 1 | 1 | 1 | 0 | 1 | 0 |
| 38 | 0 | 1 | 1 | 3 | 1 | 1 | 61 | 40 | 36 | 57,38 | 0 | 2 | 2 | 0 | 1 |  |  |  |  |  |  | 19 |  |  |  |  | 21 |  |  |  |  | 0 | 0 | 2 | 0 | 1 | 0 | 0 | 0 |
| 39 | 0 | 2 | 1 | 4 | 0 | 2 | 121 | 88 | 64,44 | 17,36 | 0 | 5 | 13 | 0 | 1 |  |  |  |  |  |  | 62 | 13,9 | 1,1 | 13,6 | 0,97 | 26 | 14,9 | 1,3 | 13,6 | 1,25 | 0 | 0 | 1 | 0 | 1 | 0 | 1 | 0 |
| 40 | 0 | 1 | 1 | 3 | 2 | 4 | 30 | 20 | 28,2 | 46,67 | 0 | 2 | 2 | 0 | 1 |  |  |  |  |  |  | 10 | 4,8 | 1,32 | 0,7 | 0,68 | 10 | 4,3 | 1,64 | 2,7 | 0,95 | 0 | 0 | 0 | 0 | 0 | 0 | 0 | 0 |
| 41 | 0 | 2 | 1 | 5 | 2 | 2 | 50 | 40 | 26 | 70 | 1 | 1 | 13 | 0 | 1 |  |  |  |  |  |  | 30 |  |  |  |  | 10 |  |  |  |  | 0 | 0 | 0 | 1 | 0 | 0 | 0 | 0 |
| 42 | 0 | 2 | 1 | 3 | 0 | 2 | 30 | 21 | 50 | 63,3 | 1 | 4 | 13 | 0 | 0 | 8 | 11 | 9 | 10 | 8 | 9 |  |  |  |  |  |  |  |  |  |  | 0 | 1 | 1 | 0 | 0 | 0 | 0 | 0 |
| 43 | 0 | 1 | 1 | 6 | 1 | 2 | 62 | 54 | 67 |  | 1 | 2 | 3 | 0 | 1 |  |  |  |  |  |  | 45 | 7,2 | 0,8 | 3,57 | 0,56 | 9 | 6,77 | 0,76 | 7,29 | 0,81 | 0 | 0 | 0 | 0 | 0 | 0 | 0 | 0 |
| 44 | 3 |  |  |  |  |  |  |  |  |  |  |  |  |  | 1 |  |  |  |  |  |  |  |  |  |  |  |  |  |  |  |  |  |  |  |  |  |  |  |  |
| 45 | 0 | 3 | 1 | 3 | 2 | 1 | 605 | 416 | 22,2 | 58 | 0 | 2 | 13 | 0 | 1 |  |  |  |  |  |  | 216 |  |  |  |  | 200 |  |  |  |  | 0 | 0 | 0 | 0 | 0 | 0 | 1 | 0 |
| 46 | 0 | 3 | 1 | 3 | 2 | 1 | 467 | 312 | 23,96 | 56,55 | 0 | 2 | 13 | 0 | 1 |  |  |  |  |  |  | 172 |  |  |  |  | 140 |  |  |  |  | 0 | 0 | 0 | 0 | 0 | 0 | 1 | 0 |
| 47 | 0 | 3 | 2 | 3 | 0 | 2 | 22 | 44 | 54 | 29,63 | 1 | 3 | 13 | 0 | 0 | 2 | 22 | 3 | 22 | 3 | 22 |  |  |  |  |  |  |  |  |  |  | 0 | 0 | 1 | 0 | 0 | 0 | 0 | 0 |
| 48 | 0 | 3 | 1 | 3 | 1 | 1 | 75 | 50 | 38 | 100 | 0 | 2 | 8 | 0 | 0 | 6 | 25 | 17 | 25 | 17 | 25 |  |  |  |  |  |  |  |  |  |  | 0 | 0 | 0 | 1 | 0 | 0 | 1 | 0 |
| 49 | 0 | 2 | 1 | 4 | 2 | 2 | 63 | 57 | 29,4 | 54 | 1 | 1 | 7 | 0 | 0 | 21 | 39 | 12 | 18 | 6 | 6 |  |  |  |  |  |  |  |  |  |  | 0 | 0 | 0 | 0 | 0 | 0 | 0 | 0 |
| 50 | 0 | 1 | 1 | 3 | 1 | 3 | 120 | 80 | 41,3 | 44,2 | 1 | 2 | 3 | 0 | 1 |  |  |  |  |  |  | 40 |  |  |  |  | 40 |  |  |  |  | 1 | 1 | 0 | 0 | 1 | 0 | 1 | 1 |
| 51 | 0 | 2 | 1 | 6 | 2 | 3 | 84 | 71 | 45,5 | 67,86 | 1 | 1 | 5 | 0 | 1 |  |  |  |  |  |  | 57 | 82,58 | 51,36 | 45,46 | 34,45 | 14 | 85,5 | 37,3 | 63,5 | 34,8 | 1 | 0 | 0 | 0 | 0 | 0 | 0 | 0 |
| 52 | 0 | 1 | 1 | 3 | 2 | 1 | 38 | 26 | 23,68 | 84,5 | 1 | 3 | 11 | 1 | 1 |  |  |  |  |  |  | 13 | 8 | 3 | 16,23 | 4,17 | 13 | 7,08 | 2,33 | 11,39 | 3,95 | 0 | 0 | 0 | 1 | 1 | 0 | 0 | 0 |
| 53 | 0 | 3 | 1 | 3 | 0 | 4 | 923 | 534 | 42,64 | 51,67 | 1 | 1 | 7 | 0 | 1 |  |  |  |  |  |  | 265 | 29,8 | 10,3 | 19 | 11,1 | 269 | 29,4 | 9,4 | 20,6 | 10 | 1 | 0 | 0 | 0 | 1 | 0 | 1 | 0 |
| 54 | 0 | 3 | 1 | 3 | 1 | 2 | 471 | 316 | 36 | 43 | 0 | 2 | 2 | 0 | 0 | 107 | 154 | 107 | 162 | 108 | 155 |  |  |  |  |  |  |  |  |  |  | 0 | 1 | 0 | 0 | 1 | 0 | 1 | 1 |
| 55 | 0 | 1 | 1 | 3 | 1 | 1 | 30 | 20 | 59,33 | 66,67 | 0 | 3 | 4 | 0 | 0 | 3 | 10 | 8 | 10 | 9 | 10 |  |  |  |  |  |  |  |  |  |  | 1 | 0 | 2 | 0 | 1 | 0 | 0 | 0 |
| 56 | 0 | 1 | 1 | 4 | 0 | 3 | 42 | 27 | 31,86 | 78,57 | 1 | 1 | 13 | 0 | 1 |  |  |  |  |  |  | 15 | 4,92 | 1,82 | 3,51 | 1,99 | 12 | 5,13 | 3,73 | 3,83 | 1,9 | 0 | 0 | 1 | 1 | 0 | 0 | 0 | 0 |
| 57 | 0 | 1 | 1 | 3 | 1 | 1 | 45 | 30 | 58,4 | 0 | 0 | 2 | 2 | 0 | 1 |  |  |  |  |  |  | 15 |  |  |  |  | 15 |  |  |  |  | 0 | 0 | 2 | 0 | 0 | 0 | 0 | 0 |
| 58 | 0 | 1 | 1 | 3 | 2 | 2 | 29 | 17 | 29,9 | 86 | 1 | 2 | 13 | 0 | 1 |  |  |  |  |  |  | 11 | 3 | 0,7 | 3,4 | 0,7 | 6 | 2,9 | 0,8 | 3,6 | 0,6 | 0 | 0 | 0 | 0 | 1 | 0 | 0 | 0 |
| 59 | 0 | 3 | 1 | 3 | 1 | 4 | 325 | 220 | 63,23 | 61,36 | 1 | 2 | 3 | 1 | 1 |  |  |  |  |  |  | 108 | 9,3 | 4 | 7,07 | 4,4 | 112 | 8,9 | 3,3 | 6,5 | 4,8 | 1 | 1 | 0 | 1 | 1 | 1 | 1 | 1 |
| 60 | 0 | 2 | 1 | 5 | 0 | 4 | 105 | 79 | 39,5 | 76,56 | 1 | 1 | 9 | 0 | 0 | 15 | 53 | 14 | 26 | 14 | 26 |  |  |  |  |  |  |  |  |  |  | 0 | 0 | 1 | 1 | 1 | 0 | 1 | 0 |
| 61 | 0 | 1 | 1 | 3 | 2 | 4 | 22 | 14 | 45,8 | 45,4 | 1 | 4 | 10 | 0 | 1 |  |  |  |  |  |  | 7 | 95 | 5 | 96 | 7,94 | 7 | 95 | 5 | 93 | 5 | 1 | 0 | 0 | 1 | 0 | 0 | 0 | 0 |
| 62 | 0 | 1 | 1 | 3 | 0 | 1 | 63 | 42 | 27 | 100 | 0 | 2 | 2 | 0 | 1 |  |  |  |  |  |  | 21 |  |  |  |  | 21 |  |  |  |  | 0 | 0 | 2 | 0 | 0 | 0 | 0 | 0 |
| 63 | 0 | 1 | 1 | 3 | 2 | 2 | 28 | 18 | 28,71 | 100 | 1 | 2 | 9 | 0 | 1 |  |  |  |  |  |  | 8 | 21,38 | 7 | 4,75 | 7 | 10 | 23,6 | 7 | 14,3 | 7 | 0 | 0 | 0 | 0 | 0 | 0 | 0 | 0 |
| 64 | 0 | 1 | 2 | 3 | 0 | 1 | 44 | 88 | 9,02 | 26,1 | 1 | 3 | 12 | 1 | 1 |  |  |  |  |  |  | 44 |  |  |  |  | 44 |  |  |  |  | 0 | 0 | 2 | 0 | 0 | 0 | 1 | 0 |
| 65 | 0 | 1 | 1 | 3 | 0 | 1 | 117 | 78 | 10,5 | 37,6 | 0 | 2 | 2 | 1 | 1 |  |  |  |  |  |  | 39 |  |  |  |  | 39 |  |  |  |  | 0 | 0 | 0 | 1 | 0 | 0 | 1 | 0 |
| 66 | 0 | 2 | 1 | 3 | 0 | 1 | 89 | 77 |  |  | 0 | 2 | 2 | 0 | 1 |  |  |  |  |  |  | 60 |  |  |  |  | 17 |  |  |  |  | 1 | 0 | 1 | 1 | 0 | 0 | 0 | 0 |
| 67 | 0 | 3 | 1 | 4 | 0 | 1 | 450 | 300 |  |  | 0 | 4 | 13 | 0 | 1 |  |  |  |  |  |  | 150 |  |  |  |  | 150 |  |  |  |  | 1 | 0 | 1 | 0 | 0 | 0 | 1 | 0 |
| 68 | 0 | 2 | 1 | 3 | 2 | 2 | 36 | 26 | 33,8 | 100 | 1 | 1 | 13 | 1 | 0 | 9,5 | 13 | 12,5 | 13 | 11,5 | 13 |  |  |  |  |  |  |  |  |  |  | 0 | 0 | 1 | 1 | 1 | 0 | 0 | 0 |
| 69 | 0 | 2 | 1 | 4 | 2 | 3 | 94 | 72 | 33,67 | 76,19 | 1 | 4 | 6 | 1 | 1 |  |  |  |  |  |  | 49 |  |  |  |  | 23 |  |  |  |  | 0 | 0 | 0 | 0 | 0 | 0 | 0 | 0 |
| 70 | 0 | 2 | 1 | 5 | 2 | 3 | 53 | 43 | 40 | 87,88 | 1 | 4 | 6 | 1 | 1 |  |  |  |  |  |  | 32 |  |  |  |  | 11 |  |  |  |  | 0 | 0 | 0 | 0 | 0 | 0 | 0 | 0 |
| 71 | 0 | 2 | 1 | 3 | 1 | 1 | 75 | 50 | 56,9 | 45,33 | 0 | 2 | 2 | 0 | 1 |  |  |  |  |  |  | 25 |  |  |  |  | 25 |  |  |  |  | 0 | 0 | 1 | 1 | 0 | 0 | 1 | 0 |
| 72 | 0 | 1 | 1 | 3 | 0 | 4 | 88 | 58 | 28,3 | 50 | 1 | 4 | 13 | 0 | 0 | 25 | 30 | 24 | 28 | 25 | 30 |  |  |  |  |  |  |  |  |  |  | 0 | 0 | 1 | 0 | 0 | 0 | 1 | 0 |
| 73 | 0 | 2 | 1 | 5 | 1 | 1 | 150 | 100 | 33 |  | 0 | 2 | 2 | 0 | 1 |  |  |  |  |  |  | 75 |  |  |  |  | 25 |  |  |  |  | 0 | 0 | 1 | 1 | 0 | 0 | 1 | 0 |
| 74 | 0 | 1 | 1 | 5 | 1 | 1 | 125 | 75 | 43 | 69,6 | 0 | 2 | 2 | 0 | 1 |  |  |  |  |  |  | 50 |  |  |  |  | 25 |  |  |  |  | 1 | 0 | 1 | 1 | 1 | 0 | 1 | 0 |
| 75 | 0 | 1 | 1 | 3 | 2 | 1 | 30 | 20 | 13,7 | 60 | 0 | 2 | 8 | 0 | 1 |  |  |  |  |  |  | 10 | 47 | 7,8 | 28,2 | 7,9 | 10 | 46,7 | 7,6 | 37,4 | 8,5 | 0 | 0 | 0 | 0 | 1 | 0 | 0 | 0 |
| 76 | 0 | 2 | 1 | 4 | 0 | 1 | 402 | 270 | 28,9 | 100 | 0 | 4 | 13 | 0 | 0 | 8 | 140 | 70 | 130 | 92 | 132 |  |  |  |  |  |  |  |  |  |  | 0 | 0 | 2 | 1 | 1 | 0 | 1 | 0 |
| 77 | 0 | 2 | 1 | 4 | 1 | 3 | 33 | 22 | 28 | 100 | 1 | 2 | 3 | 0 | 1 |  |  |  |  |  |  | 11 | 147,1 | 126,6 | 32,1 | 24,24 | 11 | 155,5 | 134,4 | 103 | 91 | 1 | 0 | 0 | 0 | 1 | 0 | 0 | 0 |
| 78 | 0 | 1 | 1 | 6 | 1 | 1 | 53 | 32 | 41,89 | 60 | 1 | 2 | 3 | 0 | 1 |  |  |  |  |  |  | 16 | 3,8 | 1,5 | 5 | 1,6 | 16 | 3,5 | 0,9 | 3,7 | 0,7 | 0 | 0 | 2 | 0 | 0 | 0 | 0 | 0 |
| 79 | 1 |  |  |  |  |  |  |  |  |  |  |  |  |  | 1 |  |  |  |  |  |  |  |  |  |  |  |  |  |  |  |  |  |  |  |  |  |  |  |  |
| 80 | 0 | 3 | 1 | 3 | 2 | 4 | 286 | 190 | 15,6 | 44,4 | 1 | 1 | 13 | 0 | 1 |  |  |  |  |  |  | 92 |  |  |  |  | 98 |  |  |  |  | 0 | 0 | 0 | 0 | 1 | 0 | 1 | 0 |
| 81 | 0 | 1 | 1 | 3 | 1 | 1 | 148 | 97 | 61,57 | 27,03 | 0 | 2 | 2 | 0 | 1 |  |  |  |  |  |  | 48 |  |  |  |  | 49 |  |  |  |  | 0 | 0 | 2 | 0 | 0 | 0 | 1 | 0 |
| 82 | 0 | 1 | 1 | 3 | 0 | 1 | 84 | 54 | 2,1 | 52,1 | 0 | 1 | 13 | 0 | 0 | 10 | 30 | 5 | 24 | 16 | 30 |  |  |  |  |  |  |  |  |  |  | 0 | 0 | 1 | 1 | 1 | 0 | 1 | 0 |
| 83 | 0 | 1 | 1 | 4 | 1 | 1 | 31 | 21 | 41,31 | 58,06 | 1 | 2 | 3 | 0 | 1 |  |  |  |  |  |  | 11 | 7 | 0,8 | 2,7 | 1,8 | 10 | 6,4 | 1,2 | 6 | 0,9 | 1 | 0 | 0 | 1 | 1 | 0 | 0 | 0 |
| 84 | 0 | 1 | 1 | 4 | 2 | 4 | 59 | 44 | 34,9 | 71,67 | 1 | 5 | 7 | 0 | 0 | 17 | 28 | 9 | 16 | 15 | 15 |  |  |  |  |  |  |  |  |  |  | 0 | 0 | 0 | 0 | 1 | 0 | 0 | 0 |
| 85 | 0 | 1 | 1 | 3 | 2 | 3 | 33 | 22 | 50,77 | 100 | 1 | 2 | 13 | 0 | 1 |  |  |  |  |  |  | 11 | 3,1 | 1 | 2,25 | 1 | 11 | 3,4 | 0,7 | 3,3 | 0,7 | 1 | 0 | 0 | 0 | 0 | 0 | 0 | 0 |
| 86 | 0 | 1 | 1 | 5 | 0 | 3 | 104 | 65 | 39 | 0 | 1 | 1 | 7 | 0 | 0 | 24 | 50 | 9 | 15 | 12 | 39 |  |  |  |  |  |  |  |  |  |  | 0 | 0 | 1 | 1 | 0 | 0 | 1 | 0 |
| 87 | 0 | 2 | 1 | 4 | 2 | 2 | 30 | 24 | 32,35 | 50 | 1 | 2 | 13 | 0 | 1 |  |  |  |  |  |  | 17 | 2,99 | 0,36 | 2,17 | 0,55 | 7 | 3,14 | 0,68 | 2,53 | 0,9 | 0 | 0 | 0 | 0 | 1 | 0 | 0 | 0 |
| 88 | 0 | 1 | 1 | 3 | 2 | 1 | 262 | 175 | 39 | 76 | 1 | 2 | 13 | 0 | 1 |  |  |  |  |  |  | 87 |  |  |  |  | 88 |  |  |  |  | 0 | 1 | 0 | 1 | 1 | 1 | 1 | 1 |
| 89 | 0 | 1 | 1 | 4 | 1 | 1 | 48 | 38 | 40,95 | 50 | 0 | 2 | 1 | 1 | 1 |  |  |  |  |  |  | 19 | 50,47 | 8,83 | 43,53 | 9,99 | 19 | 49,32 | 13,49 | 45,21 | 10,82 | 1 | 0 | 0 | 1 | 0 | 0 | 0 | 0 |
| 90 | 0 | 3 | 1 | 3 | 0 | 4 | 215 | 158 | 50 | 59 | 1 | 5 | 13 | 0 | 1 |  |  |  |  |  |  | 100 | 6 | 0,9 | 5,6 | 0,90 | 58 | 6 | 1,2 | 6,2 | 1,2 | 0 | 0 | 1 | 0 | 0 | 0 | 1 | 0 |
| 91 | 0 | 1 | 1 | 4 | 2 | 1 | 44 | 33 | 56,5 | 0 | 0 | 2 | 1 | 0 | 1 |  |  |  |  |  |  | 22 | 6,91 | 2,28 | 5,28 | 1,66 | 11 | 7,27 | 2,49 | 7,54 | 2,29 | 0 | 0 | 0 | 0 | 0 | 0 | 0 | 0 |
| 92 | 0 | 2 | 1 | 3 | 0 | 1 | 100 | 64 | 28,59 | 45 | 1 | 4 | 13 | 0 | 0 | 18 | 31 | 28 | 33 | 32 | 36 |  |  |  |  |  |  |  |  |  |  | 1 | 0 | 1 | 0 | 1 | 0 | 1 | 0 |
| 93 | 0 | 1 | 1 | 3 | 0 | 3 | 98 | 63 | 37,3 | 37 | 1 | 1 | 13 | 0 | 1 |  |  |  |  |  |  | 39 | 6 | 4,5 | 0,7 | 1,8 | 24 | 5 | 4,6 | 1 | 2,5 | 0 | 0 | 2 | 0 | 0 | 0 | 1 | 0 |
| 94 | 0 | 3 | 1 | 4 | 0 | 3 | 1218 | 909 | 42,55 | 51,72 | 1 | 5 | 7 | 0 | 0 | 387 | 600 | 226 | 309 | 219 | 309 |  |  |  |  |  |  |  |  |  |  | 0 | 0 | 1 | 0 | 1 | 0 | 1 | 0 |
| 95 | 0 | 1 | 1 | 5 | 2 | 2 | 20 | 16 | 51 | 0 | 1 | 1 | 13 | 0 | 1 |  |  |  |  |  |  | 12 | 22,67 | 23,67 | 51,33 | 38,17 | 4 | 21 | 33 | 33 | 47 | 0 | 0 | 0 | 0 | 1 | 0 | 0 | 0 |
| 96 | 0 | 2 | 1 | 3 | 0 | 4 | 150 | 100 | 37,5 | 100 | 1 | 1 | 9 | 0 | 0 | 6 | 50 | 17 | 50 | 16 | 50 |  |  |  |  |  |  |  |  |  |  | 0 | 0 | 1 | 1 | 0 | 0 | 1 | 0 |
| 97 | 0 | 1 | 1 | 3 | 1 | 1 | 60 | 39 | 68,32 | 43,33 | 0 | 2 | 2 | 0 | 1 |  |  |  |  |  |  | 19 | 61,8 | 11,8 | 34 | 16,9 | 20 | 66,7 | 10 | 66,7 | 10 | 0 | 0 | 1 | 1 | 1 | 0 | 0 | 0 |
| 98 | 2 |  |  |  |  |  |  |  |  |  |  |  |  |  | 1 |  |  |  |  |  |  |  |  |  |  |  |  |  |  |  |  |  |  |  |  |  |  |  |  |
| 99 | 0 | 3 | 1 | 3 | 1 | 2 | 70 | 46 | 46,34 | 57,14 | 1 | 2 | 4 | 0 | 1 |  |  |  |  |  |  | 23 | 16,4 | 4,9 | 9,13 | 5,53 | 23 | 17,5 | 6 | 15,2 | 4,9 | 1 | 1 | 0 | 1 | 1 | 0 | 0 | 0 |
| 100 | 0 | 1 | 1 | 3 | 1 | 1 | 514 | 346 | 11,3 | 48,62 | 0 | 2 | 2 | 0 | 1 |  |  |  |  |  |  | 174 |  |  |  |  | 172 |  |  |  |  | 0 | 1 | 0 | 1 | 1 | 0 | 1 | 1 |
| 101 | 1 |  |  |  |  |  |  |  |  |  |  |  |  |  | 1 |  |  |  |  |  |  |  |  |  |  |  |  |  |  |  |  |  |  |  |  |  |  |  |  |
| 102 | 0 | 1 | 1 | 3 | 1 | 3 | 114 | 75 | 48,1 | 58,5 | 1 | 2 | 3 | 0 | 1 |  |  |  |  |  |  | 35 |  |  |  |  | 40 |  |  |  |  | 0 | 0 | 0 | 1 | 1 | 0 | 1 | 0 |
| 103 | 0 | 2 | 1 | 8 | 0 | 1 | 60 | 36 |  |  | 0 | 2 | 2 | 0 | 1 |  |  |  |  |  |  | 24 |  |  |  |  | 12 |  |  |  |  | 0 | 0 | 1 | 1 | 0 | 0 | 0 | 0 |
| 104 | 0 | 1 | 1 | 3 | 1 | 4 | 66 | 51 | 49,76 | 65,09 | 1 | 2 | 3 | 1 | 1 |  |  |  |  |  |  | 32 | 3,6 | 2,2 | 3,16 | 2,36 | 19 | 3,7 | 2,7 | 2,46 | 1,68 | 0 | 1 | 0 | 0 | 0 | 0 | 0 | 0 |
| 105 | 0 | 1 | 1 | 4 | 2 | 2 | 27 | 18 | 29,77 | 100 | 1 | 2 | 11 | 0 | 1 |  |  |  |  |  |  | 9 | 100 | 36,83 | 81,67 | 36,83 | 9 | 100 | 32,73 | 60,56 | 32,73 | 0 | 0 | 0 | 1 | 1 | 0 | 0 | 0 |
| 106 | 0 | 1 | 1 | 4 | 2 | 2 | 40 | 30 | 47,48 | 65 | 1 | 1 | 5 | 1 | 1 |  |  |  |  |  |  | 20 | 62,5 | 26,6 | 34 | 18,34 | 10 | 69 | 19 | 66 | 25 | 0 | 0 | 0 | 0 | 0 | 0 | 0 | 0 |
| 107 | 0 | 1 | 1 | 3 | 1 | 1 | 90 | 60 | 57,25 | 43,33 | 0 | 2 | 2 | 0 | 1 |  |  |  |  |  |  | 30 |  |  |  |  | 30 |  |  |  |  | 0 | 0 | 0 | 1 | 1 | 0 | 1 | 0 |
| 108 | 0 | 2 | 1 | 4 | 1 | 1 | 100 | 75 | 40 | 100 | 0 | 3 | 4 | 0 | 1 |  |  |  |  |  |  | 50 |  |  |  |  | 25 |  |  |  |  | 0 | 0 | 0 | 0 | 1 | 0 | 1 | 0 |
| 109 | 0 | 2 | 1 | 3 | 2 | 4 | 118 | 80 | 66.05 | 48,78 | 1 | 2 | 9 | 0 | 1 |  |  |  |  |  |  | 38 | 18,2 | 10 | 15,3 | 10 | 42 | 16,4 | 10 | 14,4 | 10 | 0 | 1 | 0 | 1 | 1 | 0 | 1 | 1 |
| 110 | 0 | 3 | 1 | 3 | 1 | 3 | 297 | 221 | 43 | 88 | 1 | 2 | 3 | 1 | 1 |  |  |  |  |  |  | 145 |  |  |  |  | 76 |  |  |  |  | 1 | 1 | 0 | 1 | 1 | 1 | 1 | 1 |
| 111 | 0 | 3 | 3 | 6 | 0 | 4 | 741 | 515 | 48,71 | 15,27 | 1 | 5 | 13 | 0 | 1 |  |  |  |  |  |  | 288 | 6,85 | 0,54 | 5,97 | 0,54 | 227 | 6,89 | 0,54 | 6,83 | 0,54 | 0 | 0 | 1 | 0 | 1 | 0 | 1 | 0 |
| 112 | 0 | 1 | 1 | 4 | 2 | 1 | 80 | 60 | 8,73 |  | 0 | 2 | 2 | 0 | 1 |  |  |  |  |  |  | 40 | 4,6 | 0,65 | 1,95 | 1,3 | 20 | 4,6 | 0,6 | 4,3 | 0,6 | 0 | 0 | 2 | 0 | 1 | 0 | 0 | 0 |
| 113 | 0 | 3 | 1 | 3 | 2 | 4 | 29 | 20 | 26,2 |  | 1 | 1 | 13 | 0 | 1 |  |  |  |  |  |  | 10 | 3,5 | 0,56 | 2,6 | 1,09 | 10 | 3,4 | 0,65 | 3,6 | 0,6 | 0 | 0 | 0 | 0 | 1 | 0 | 0 | 0 |
| 114 | 0 | 3 | 1 | 5 | 0 | 2 | 180 | 142 | 37 | 0 | 1 | 2 | 1 | 0 | 1 |  |  |  |  |  |  | 100 |  |  |  |  | 42 |  |  |  |  | 0 | 0 | 1 | 1 | 0 | 0 | 1 | 0 |
| 115 | 0 | 2 | 1 | 3 | 0 | 1 | 90 | 60 | 41 |  | 0 | 2 | 1 | 0 | 1 |  |  |  |  |  |  | 30 | 3,3 | 0,7 | 4,5 | 0,7 | 30 | 3,4 | 0,6 | 3,3 | 3,83 | 0 | 0 | 1 | 1 | 0 | 0 | 1 | 0 |
| 116 | 0 | 1 | 1 | 3 | 0 | 2 | 194 | 136 | 44,32 | 42,78 | 1 | 5 | 7 | 1 | 0 | 48 | 73 | 40 | 63 | 44 | 58 |  |  |  |  |  |  |  |  |  |  | 0 | 0 | 1 | 0 | 0 | 0 | 1 | 0 |
| 117 | 0 | 1 | 1 | 3 | 2 | 1 | 21 | 14 | 34,05 | 19,4 | 0 | 2 | 1 | 0 | 1 |  |  |  |  |  |  | 7 | 43,28 | 10,37 | 33,71 | 8,44 | 7 | 39,43 | 9,83 | 37,29 | 11,77 | 0 | 0 | 0 | 0 | 1 | 0 | 0 | 0 |
| 118 | 0 | 1 | 1 | 3 | 1 | 1 | 66 | 45 | 57,94 | 56,27 | 0 | 2 | 13 | 0 | 1 |  |  |  |  |  |  | 22 |  |  |  |  | 23 |  |  |  |  | 0 | 0 | 0 | 1 | 0 | 0 | 0 | 0 |
| 119 | 3 |  |  |  |  |  |  |  |  |  |  |  |  |  | 1 |  |  |  |  |  |  |  |  |  |  |  |  |  |  |  |  |  |  |  |  |  |  |  |  |
| 120 | 0 | 2 | 1 | 3 | 0 | 4 | 44 | 32 | 63,1 | 52,08 | 1 | 3 | 13 | 0 | 1 |  |  |  |  |  |  | 24 |  |  |  |  | 8 |  |  |  |  | 1 | 0 | 0 | 1 | 0 | 0 | 0 | 0 |
| 121 | 0 | 1 | 1 | 4 | 1 | 1 | 230 | 184 | 65 | 59,57 | 0 | 2 | 8 | 0 | 0 | 58 | 158 | 14 | 26 | 24 | 46 |  |  |  |  |  |  |  |  |  |  | 0 | 0 | 0 | 1 | 1 | 0 | 1 | 0 |
| 122 | 0 | 2 | 1 | 3 | 1 | 4 | 64 | 46 | 39 | 48,48 | 1 | 3 | 12 | 0 | 1 |  |  |  |  |  |  | 23 |  |  |  |  | 23 |  |  |  |  | 0 | 0 | 2 | 0 | 1 | 0 | 0 | 0 |
| 123 | 0 | 3 | 1 | 3 | 1 | 3 | 238 | 175 | 43 | 74 | 1 | 2 | 3 | 1 | 1 |  |  |  |  |  |  | 118 | 17,5 | 6,9 | 9,9 | 8,7 | 57 | 17,7 | 6,7 | 10,8 | 8,3 | 1 | 1 | 0 | 1 | 1 | 1 | 1 | 1 |
| 124 | 0 | 2 | 1 | 3 | 1 | 2 | 75 | 48 | 63 | 64,44 | 1 | 2 | 3 | 0 | 1 |  |  |  |  |  |  | 26 | 28,18 | 18,75 | 23,52 | 18,9 | 22 | 28,4 | 18,6 | 24,04 | 18,56 | 1 | 1 | 1 | 1 | 0 | 0 | 0 | 0 |
| 125 | 0 | 1 | 1 | 3 | 1 | 2 | 171 | 118 | 50 | 47,85 | 1 | 2 | 3 | 0 | 0 | 21 | 60 | 38 | 58 | 30 | 53 |  |  |  |  |  |  |  |  |  |  | 1 | 1 | 2 | 0 | 1 | 0 | 1 | 1 |
| 126 | 0 | 1 | 1 | 3 | 2 | 4 | 199 | 100 | 49,2 | 42 | 1 | 1 | 13 | 0 | 1 |  |  |  |  |  |  | 50 | 1,9 | 1,3 | 2,8 | 1,5 | 50 | 1,7 | 1,3 | 2 | 1,3 | 0 | 0 | 0 | 0 | 0 | 0 | 1 | 0 |
| 127 | 0 | 1 | 1 | 3 | 2 | 4 | 165 | 123 | 78,3 | 0,58 | 1 | 1 | 13 | 0 | 1 |  |  |  |  |  |  | 85 | 16 | 10 | 19 | 10 | 38 | 15,8 | 9,4 | 16,5 | 9,3 | 0 | 0 | 0 | 0 | 1 | 0 | 1 | 0 |
| 128 | 0 | 1 | 2 | 4 | 1 | 1 | 13 | 26 | 22 | 100 | 1 | 3 | 12 | 0 | 1 |  |  |  |  |  |  | 13 |  |  |  |  | 13 |  |  |  |  | 0 | 0 | 0 | 0 | 0 | 0 | 0 | 0 |
| 129 | 0 | 2 | 3 | 6 | 2 | 3 | 68 | 57 | 40,9 | 50 | 1 | 4 | 6 | 0 | 1 |  |  |  |  |  |  | 51 |  |  |  |  | 6 |  |  |  |  | 0 | 0 | 0 | 0 | 0 | 0 | 0 | 0 |
| 130 | 2 |  |  |  |  |  |  |  |  |  |  |  |  |  | 0 |  |  |  |  |  |  |  |  |  |  |  |  |  |  |  |  |  |  |  |  |  |  |  |  |
| 131 | 0 | 1 | 1 | 3 | 0 | 2 | 35 | 27 |  |  | 1 | 3 | 9 | 0 | 1 |  |  |  |  |  |  | 17 | 60,8 | 11 | 25,5 | 24 | 10 | 57 | 7 | 53,2 | 11,2 | 0 | 0 | 2 | 0 | 1 | 0 | 0 | 0 |
| 132 | 0 | 2 | 1 | 3 | 1 | 4 | 120 | 78 | 70 | 0 | 1 | 2 | 13 | 0 | 1 |  |  |  |  |  |  | 38 | 19 | 5,39 | 9,5 | 5,39 | 40 | 17,5 | 6 | 9,5 | 6 | 1 | 0 | 1 | 1 | 0 | 0 | 1 | 0 |
| 133 | 0 | 1 | 1 | 4 | 2 | 2 | 30 | 21 | 45,1 | 70 | 1 | 1 | 5 | 0 | 1 |  |  |  |  |  |  | 14 | 120,28 | 58,16 | 59,9 | 41,27 | 7 | 118,86 | 108,65 | 117,29 | 133,7 | 0 | 0 | 0 | 0 | 1 | 0 | 0 | 0 |
| 134 | 0 | 1 | 1 | 3 | 2 | 1 | 30 | 20 | 9 | 50 | 0 | 3 | 1 | 0 | 1 |  |  |  |  |  |  | 10 |  |  |  |  | 10 |  |  |  |  | 0 | 0 | 0 | 1 | 0 | 0 | 0 | 0 |
| 135 | 0 | 2 | 1 | 3 | 1 | 1 | 161 | 107 | 29,57 | 100 | 0 | 2 | 8 | 0 | 1 |  |  |  |  |  |  | 54 | 11,1 | 5,73 | 7,43 | 5,37 | 53 | 10,76 | 5,6 | 6,7 | 5,6 | 0 | 1 | 0 | 1 | 1 | 0 | 1 | 1 |
| 136 | 0 | 2 | 1 | 3 | 2 | 3 | 141 | 96 | 60 | 40 | 1 | 2 | 3 | 0 | 1 |  |  |  |  |  |  | 47 | 3,5 | 2,04 | 2,4 | 2,04 | 49 | 3,5 | 1,9 | 4 | 1,9 | 0 | 0 | 0 | 0 | 1 | 0 | 1 | 0 |
| 137 | 0 | 1 | 1 | 3 | 2 | 3 | 41 | 27 | 54,6 | 72,22 | 1 | 2 | 9 | 0 | 1 |  |  |  |  |  |  | 14 | 27,7 | 7,5 | 14,3 | 7,5 | 13 | 31,2 | 7,5 | 16,2 | 7,5 | 0 | 0 | 0 | 0 | 0 | 0 | 0 | 0 |
| 138 | 0 | 1 | 2 | 3 | 0 | 2 | 38 | 76 | 9,95 | 0 | 1 | 1 | 13 | 1 | 1 |  |  |  |  |  |  | 38 |  |  |  |  | 38 |  |  |  |  | 0 | 0 | 1 | 1 | 0 | 0 | 1 | 0 |
| 139 | 0 | 2 | 1 | 3 | 2 | 3 | 78 | 53 | 73,6 | 34,6 | 1 | 3 | 13 | 0 | 1 |  |  |  |  |  |  | 25 | 109,8 | 12 | 113,1 | 11,7 | 28 | 110 | 12,2 | 108,3 | 14,8 | 0 | 0 | 0 | 1 | 0 | 0 | 1 | 0 |
| 140 | 1 |  |  |  |  |  |  |  |  |  |  |  |  |  | 0 |  |  |  |  |  |  |  |  |  |  |  |  |  |  |  |  |  |  |  |  |  |  |  |  |
| 141 | 0 | 1 | 1 | 3 | 0 | 1 | 368 | 272 | 25,25 | 100 | 0 | 2 | 2 | 0 | 1 |  |  |  |  |  |  | 183 |  |  |  |  | 89 |  |  |  |  | 0 | 1 | 1 | 1 | 0 | 0 | 1 | 0 |
| 142 | 0 | 1 | 1 | 3 | 2 | 1 | 59 | 39 | 30,93 | 100 | 0 | 2 | 2 | 0 | 1 |  |  |  |  |  |  | 21 |  |  |  |  | 18 |  |  |  |  | 0 | 0 | 0 | 0 | 1 | 0 | 0 | 0 |
| 143 | 0 | 1 | 1 | 3 | 0 | 1 | 180 | 119 | 47,02 | 59,44 | 0 | 2 | 13 | 0 | 1 |  |  |  |  |  |  | 58 |  |  |  |  | 61 |  |  |  |  | 0 | 0 | 1 | 1 | 0 | 0 | 1 | 0 |
| 144 | 0 | 1 | 1 | 3 | 1 | 1 | 33 | 23 | 52,55 |  | 0 | 3 | 4 | 0 | 1 |  |  |  |  |  |  | 10 |  |  |  |  | 13 |  |  |  |  | 1 | 1 | 2 | 1 | 0 | 0 | 0 | 0 |
| 145 | 0 | 1 | 1 | 3 | 0 | 3 | 55 | 36 | 41,81 | 76,36 | 1 | 4 | 6 | 0 | 1 |  |  |  |  |  |  | 18 |  |  |  |  | 18 |  |  |  |  | 0 | 0 | 1 | 0 | 0 | 0 | 0 | 0 |
| 146 | 0 | 3 | 2 | 3 | 1 | 1 | 27 | 54 | 49,7 | 92,6 | 0 | 1 | 4 | 0 | 1 |  |  |  |  |  |  | 27 |  |  |  |  | 27 |  |  |  |  | 0 | 0 | 2 | 0 | 0 | 0 | 1 | 0 |
| 147 | 0 | 3 | 1 | 3 | 1 | 1 | 96 | 63 | 49,5 | 100 | 0 | 2 | 8 | 1 | 1 |  |  |  |  |  |  | 32 |  |  |  |  | 31 |  |  |  |  | 0 | 0 | 2 | 0 | 1 | 0 | 1 | 0 |
| 148 | 0 | 1 | 1 | 5 | 2 | 2 | 34 | 29 | 33,5 | 91 | 1 | 2 | 1 | 0 | 1 |  |  |  |  |  |  | 20 |  |  |  |  | 9 |  |  |  |  | 0 | 0 | 1 | 1 | 0 | 0 | 0 | 0 |
| 149 | 0 | 1 | 2 | 3 | 0 | 1 | 6 | 12 | 54 | 50 | 1 | 4 | 10 | 0 | 1 |  |  |  |  |  |  | 6 | 104 | 19,6 | 101 | 22 | 6 | 104 | 19,6 | 107 | 17 | 0 | 0 | 2 | 0 | 1 | 0 | 0 | 0 |
| 150 | 0 | 2 | 1 | 3 | 2 | 3 | 40 | 26 | 32 | 100 | 1 | 2 | 13 | 0 | 0 | 11 | 14 | 9 | 12 | 9 | 14 |  |  |  |  |  |  |  |  |  |  | 0 | 0 | 0 | 0 | 0 | 0 | 0 | 0 |
| 151 | 0 | 2 | 2 | 3 | 0 | 1 | 35 | 70 | 75 | 42,9 | 1 | 2 | 3 | 0 | 1 |  |  |  |  |  |  | 35 |  |  |  |  | 35 |  |  |  |  | 0 | 0 | 1 | 1 | 1 | 0 | 1 | 0 |
| 152 | 0 | 1 | 1 | 4 | 2 | 1 | 105 | 81 | 65,7 | 0 | 0 | 2 | 1 | 1 | 1 |  |  |  |  |  |  | 56 | 6,9 | 2,49 | 5,64 | 2,49 | 25 | 6,9 | 2,2 | 7,12 | 2,2 | 0 | 0 | 0 | 0 | 0 | 0 | 0 | 0 |
| 153 | 0 | 1 | 1 | 3 | 1 | 2 | 70 | 46 | 48,31 | 68,57 | 1 | 2 | 9 | 0 | 1 |  |  |  |  |  |  | 22 | 31,8 | 16,1 | 27,7 | 16,1 | 24 | 32,34 | 16,2 | 23 | 16,2 | 0 | 0 | 0 | 0 | 0 | 0 | 0 | 0 |
| 154 | 0 | 1 | 1 | 3 | 1 | 1 | 41 | 27 | 55 |  | 0 | 2 | 13 | 0 | 1 |  |  |  |  |  |  | 14 |  |  |  |  | 13 |  |  |  |  | 0 | 0 | 2 | 0 | 0 | 0 | 0 | 0 |
| 155 | 0 | 1 | 1 | 3 | 1 | 1 | 18 | 12 | 41 | 50 | 0 | 2 | 2 | 0 | 1 |  |  |  |  |  |  | 6 | 2,7 | 0,2 | 1 | 0,25 | 6 | 2,38 | 0,47 | 2,03 | 0,42 | 1 | 0 | 0 | 0 | 1 | 0 | 0 | 0 |
| 156 | 0 | 1 | 1 | 3 | 0 | 1 | 59 | 49 | 38,6 | 76,3 | 1 | 4 | 13 | 0 | 0 | 0 | 39 | 3 | 10 | 4 | 10 |  |  |  |  |  |  |  |  |  |  | 0 | 0 | 2 | 0 | 0 | 0 | 0 | 0 |
| 157 | 0 | 3 | 1 | 3 | 1 | 4 | 1007 | 691 | 62,81 | 68,82 | 1 | 2 | 13 | 0 | 0 | 153 | 326 | 179 | 365 | 224 | 316 |  |  |  |  |  |  |  |  |  |  | 1 | 1 | 0 | 1 | 1 | 1 | 1 | 1 |
| 158 | 0 | 1 | 1 | 3 | 2 | 2 | 36 | 24 | 12,8 | 66,67 | 1 | 2 | 3 | 0 | 0 | 6 | 13 | 10 | 11 | 12 | 12 |  |  |  |  |  |  |  |  |  |  | 1 | 0 | 0 | 1 | 1 | 0 | 0 | 0 |
| 159 | 0 | 1 | 1 | 3 | 2 | 2 | 41 | 28 | 43,2 | 43,9 | 1 | 4 | 10 | 0 | 1 |  |  |  |  |  |  | 14 | 103,6 | 10 | 97,2 | 8,4 | 14 | 100,1 | 10,4 | 92,5 | 9,8 | 0 | 0 | 2 | 1 | 1 | 0 | 0 | 0 |
| 160 | 0 | 1 | 1 | 4 | 2 | 2 | 45 | 35 | 10,3 | 33,33 | 1 | 4 | 6 | 0 | 1 |  |  |  |  |  |  | 24 | 50,98 | 8,53 | 49,41 | 8,8 | 11 | 47,84 | 8,54 | 47,52 | 8,38 | 0 | 0 | 0 | 0 | 0 | 0 | 0 | 0 |
| 161 | 0 | 1 | 1 | 3 | 1 | 1 | 104 | 70 | 46 | 100 | 0 | 3 | 4 | 1 | 1 |  |  |  |  |  |  | 37 |  |  |  |  | 33 |  |  |  |  | 1 | 1 | 0 | 0 | 1 | 0 | 1 | 1 |
| 162 | 0 | 2 | 1 | 4 | 0 | 4 | 145 | 122 | 13,35 | 50 | 1 | 4 | 10 | 0 | 1 |  |  |  |  |  |  | 62 |  |  |  |  | 60 |  |  |  |  | 0 | 0 | 1 | 0 | 0 | 0 | 1 | 0 |
| 163 | 0 | 2 | 1 | 5 | 2 | 1 | 40 | 30 |  |  | 1 | 1 | 7 | 0 | 1 |  |  |  |  |  |  | 20 | 28,37 | 14 | 4,05 | 14 | 10 | 28,5 | 14 | 8 | 14 | 0 | 0 | 0 | 0 | 0 | 0 | 0 | 0 |
| 164 | 0 | 1 | 1 | 4 | 2 | 1 | 45 | 33 | 40,5 | 53,7 | 1 | 1 | 7 | 0 | 1 |  |  |  |  |  |  | 20 | 20,52 | 10,9 | 18,8 | 10,1 | 13 | 20,52 | 10,9 | 21,39 | 9,7 | 0 | 0 | 0 | 0 | 0 | 0 | 0 | 0 |
| 165 | 0 | 2 | 1 | 3 | 1 | 1 | 30 | 20 | 55 |  | 1 | 2 | 3 | 0 | 1 |  |  |  |  |  |  | 10 | 7,1 | 3 | 6,9 | 3 | 10 | 7,8 | 3 | 7,9 | 3 | 1 | 0 | 0 | 0 | 0 | 0 | 0 | 0 |
| 166 | 0 | 2 | 1 | 3 | 1 | 1 | 30 | 20 | 10,5 |  | 1 | 4 | 13 | 0 | 1 |  |  |  |  |  |  | 10 | 1,6 | 0,5 | 0,8 | 0,6 | 10 | 1,7 | 0,5 | 1,4 | 0,5 | 0 | 0 | 1 | 1 | 0 | 0 | 0 | 0 |
| 167 | 0 | 1 | 1 | 4 | 0 | 3 | 273 | 199 | 3,39 | 45,2 | 0 | 1 | 13 | 0 | 1 |  |  |  |  |  |  | 97 |  |  |  |  | 102 |  |  |  |  | 1 | 1 | 1 | 1 | 0 | 0 | 1 | 0 |
| 168 | 0 | 1 | 1 | 4 | 2 | 2 | 100 | 75 |  |  | 0 | 1 | 13 | 0 | 1 |  |  |  |  |  |  | 50 | 12,4 | 4,5 | 14,95 | 3,62 | 25 | 12,6 | 2,7 | 14,3 | 5,5 | 0 | 0 | 0 | 0 | 0 | 0 | 1 | 0 |
| 169 | 0 | 1 | 1 | 3 | 0 | 3 | 15 | 11 |  |  | 1 | 1 | 13 | 0 | 0 | 3 | 6 | 1 | 5 | 1 | 4 |  |  |  |  |  |  |  |  |  |  | 0 | 0 | 1 | 1 | 0 | 0 | 0 | 0 |
| 170 | 0 | 1 | 1 | 3 | 1 | 1 | 15 | 10 | 18,3 | 20 | 0 | 1 | 13 | 0 | 1 |  |  |  |  |  |  | 5 |  |  |  |  | 5 |  |  |  |  | 0 | 0 | 0 | 1 | 0 | 0 | 0 | 0 |
| 171 | 0 | 1 | 2 | 3 | 1 | 1 | 10 | 20 | 83,8 | 60 | 1 | 3 | 9 | 0 | 1 |  |  |  |  |  |  | 10 | 14,8 | 3,1 | 11,3 | 2,3 | 10 | 14,7 | 2,9 | 15,4 | 2,72 | 0 | 0 | 2 | 0 | 1 | 0 | 0 | 0 |
| 172 | 0 | 1 | 1 | 3 | 2 | 1 | 36 | 24 | 46 | 0 | 0 | 2 | 2 | 0 | 1 |  |  |  |  |  |  | 12 |  |  |  |  | 12 |  |  |  |  | 0 | 0 | 2 | 0 | 0 | 0 | 0 | 0 |
| 173 | 0 | 1 | 1 | 3 | 2 | 2 | 27 | 18 | 33,4 | 63 | 1 | 1 | 13 | 0 | 0 | 4 | 8 | 8 | 10 | 8 | 9 |  |  |  |  |  |  |  |  |  |  | 0 | 0 | 2 | 0 | 1 | 0 | 0 | 0 |
| 174 | 0 | 3 | 1 | 4 | 1 | 1 | 313 | 236 | 48 | 45,1 | 0 | 2 | 8 | 0 | 0 | 21 | 78 | 58 | 158 | 27 | 77 |  |  |  |  |  |  |  |  |  |  | 0 | 0 | 0 | 1 | 0 | 0 | 1 | 0 |
| 175 | 0 | 3 | 1 | 3 | 2 | 3 | 87 | 59 | 38,6 | 20,7 | 1 | 3 | 13 | 0 | 0 | 22 | 33 | 22 | 26 | 25 | 28 |  |  |  |  |  |  |  |  |  |  | 0 | 1 | 0 | 1 | 1 | 0 | 1 | 1 |
| 176 | 0 | 1 | 1 | 3 | 0 | 1 | 67 | 43 | 47,93 | 28,36 | 0 | 2 | 2 | 0 | 1 |  |  |  |  |  |  | 24 |  |  |  |  | 19 |  |  |  |  | 0 | 0 | 1 | 1 | 0 | 0 | 0 | 0 |
| 177 | 0 | 1 | 1 | 3 | 1 | 1 | 59 | 44 | 2,5 |  | 0 | 3 | 1 | 0 | 1 |  |  |  |  |  |  | 27 |  |  |  |  | 17 |  |  |  |  | 0 | 0 | 1 | 1 | 0 | 0 | 0 | 0 |
| 178 | 1 |  |  |  |  |  |  |  |  |  |  |  |  |  | 0 |  |  |  |  |  |  |  |  |  |  |  |  |  |  |  |  |  |  |  |  |  |  |  |  |
| 179 | 0 | 3 | 1 | 3 | 2 | 3 | 59 | 33 | 25,3 | 47 | 1 | 2 | 3 | 0 | 1 |  |  |  |  |  |  | 19 | 14,3 | 10,45 | 8,3 | 8,89 | 14 | 9,4 | 9,26 | 13,4 | 10,28 | 1 | 0 | 0 | 0 | 0 | 0 | 0 | 0 |
| 180 | 3 |  |  |  |  |  |  |  |  |  |  |  |  |  | 1 |  |  |  |  |  |  |  |  |  |  |  |  |  |  |  |  |  |  |  |  |  |  |  |  |
| 181 | 3 |  |  |  |  |  |  |  |  |  |  |  |  |  | 1 |  |  |  |  |  |  |  |  |  |  |  |  |  |  |  |  |  |  |  |  |  |  |  |  |
| 182 | 2 |  |  |  |  |  |  |  |  |  |  |  |  |  | 1 |  |  |  |  |  |  |  |  |  |  |  |  |  |  |  |  |  |  |  |  |  |  |  |  |
| 183 | 0 | 2 | 1 | 5 | 1 | 1 | 160 | 119 | 39,46 | 38,1 | 0 | 2 | 2 | 0 | 1 |  |  |  |  |  |  | 78 |  |  |  |  | 41 |  |  |  |  | 0 | 0 | 1 | 1 | 0 | 0 | 1 | 0 |
| 184 | 0 | 3 | 1 | 3 | 1 | 2 | 98 | 66 | 55,52 | 54,8 | 1 | 1 | 5 | 0 | 1 |  |  |  |  |  |  | 34 | 9,94 | 3,95 | 7,29 | 4,29 | 32 | 10,53 | 3,56 | 9,23 | 4,36 | 0 | 0 | 2 | 0 | 1 | 0 | 1 | 0 |
| 185 | 0 | 3 | 1 | 3 | 1 | 2 | 106 | 70 | 58,16 | 66 | 1 | 2 | 13 | 0 | 1 |  |  |  |  |  |  | 35 | 5,94 | 1,42 | 4,66 | 1,77 | 35 | 5,64 | 1,32 | 4,7 | 1,51 | 0 | 0 | 2 | 0 | 0 | 0 | 1 | 0 |
| 186 | 0 | 1 | 1 | 3 | 0 | 4 | 32 | 21 |  | 100 | 1 | 5 | 13 | 0 | 1 |  |  |  |  |  |  | 10 | 8,28 | 1,23 | 8 | 2,31 | 11 | 9,4 | 0,86 | 9,04 | 1,36 | 0 | 0 | 1 | 0 | 1 | 0 | 0 | 0 |
| 187 | 0 | 1 | 1 | 5 | 2 | 2 | 50 | 40 | 39 | 50 | 1 | 1 | 5 | 0 | 1 |  |  |  |  |  |  | 30 | 63,1 | 32,3 | 26,1 | 15,1 | 10 | 57,2 | 35,4 | 44,2 | 41,3 | 0 | 0 | 0 | 0 | 0 | 0 | 0 | 0 |
| 188 | 1 |  |  |  |  |  |  |  |  |  |  |  |  |  | 0 |  |  |  |  |  |  |  |  |  |  |  |  |  |  |  |  |  |  |  |  |  |  |  |  |
| 189 | 0 | 1 | 1 | 3 | 2 | 3 | 125 | 85 | 44 | 88 | 1 | 2 | 3 | 1 | 1 |  |  |  |  |  |  | 46 | 0,1 | 2,4 | 1 | 1,8 | 39 | -0,1 | 1,8 | 0,4 | 1,8 | 0 | 0 | 0 | 0 | 1 | 0 | 1 | 0 |
| 190 | 0 | 3 | 1 | 3 | 0 | 1 | 80 | 50 | 47,5 | 40 | 0 | 2 | 2 | 0 | 0 | 18 | 26 | 19 | 24 | 21 | 30 |  |  |  |  |  |  |  |  |  |  | 0 | 0 | 0 | 1 | 0 | 0 | 1 | 0 |
| 191 | 0 | 1 | 1 | 4 | 1 | 1 | 101 | 75 | 44 | 100 | 0 | 3 | 4 | 0 | 1 |  |  |  |  |  |  | 50 |  |  |  |  | 25 |  |  |  |  | 1 | 0 | 0 | 0 | 0 | 0 | 1 | 0 |
| 192 | 2 |  |  |  |  |  |  |  |  |  |  |  |  |  | 0 |  |  |  |  |  |  |  |  |  |  |  |  |  |  |  |  |  |  |  |  |  |  |  |  |
| 193 | 0 | 1 | 1 | 3 | 1 | 1 | 45 | 30 | 37,2 | 0 | 1 | 3 | 13 | 0 | 1 |  |  |  |  |  |  | 15 |  |  |  |  | 15 |  |  |  |  | 0 | 0 | 0 | 1 | 0 | 0 | 0 | 0 |
| 194 | 0 | 3 | 1 | 3 | 1 | 1 | 60 | 40 | 30 | 100 | 0 | 1 | 8 | 0 | 1 |  |  |  |  |  |  | 20 | 8,4 | 1,2 | 4,2 | 2,6 | 20 | 8,4 | 1,4 | 5,9 | 2,4 | 1 | 1 | 0 | 0 | 0 | 0 | 0 | 0 |
| 195 | 0 | 1 | 1 | 3 | 0 | 3 | 39 | 26 | 50 | 0 | 1 | 3 | 13 | 0 | 0 | 1 | 13 | 3 | 13 | 7 | 13 |  |  |  |  |  |  |  |  |  |  | 0 | 0 | 1 | 0 | 0 | 0 | 0 | 0 |
| 196 | 0 | 2 | 2 | 3 | 0 | 1 | 15 | 30 | 66 | 53,3 | 1 | 3 | 13 | 0 | 1 |  |  |  |  |  |  | 15 |  |  |  |  | 15 |  |  |  |  | 0 | 0 | 1 | 0 | 1 | 0 | 0 | 0 |
| 197 | 0 | 1 | 1 | 3 | 2 | 1 | 60 | 40 | 39,5 | 48,3 | 1 | 1 | 7 | 0 | 0 | 12 | 20 | 19 | 20 | 19 | 20 |  |  |  |  |  |  |  |  |  |  | 0 | 0 | 0 | 0 | 1 | 0 | 0 | 0 |
| 198 | 0 | 2 | 1 | 3 | 0 | 4 | 90 | 80 | 36,1 | 11 | 1 | 1 | 13 | 0 | 0 | 28 | 40 | 31 | 40 | 10 | 10 |  |  |  |  |  |  |  |  |  |  | 0 | 0 | 1 | 1 | 0 | 0 | 1 | 0 |
| 199 | 0 | 3 | 1 | 3 | 1 | 2 | 285 | 218 | 64 | 66 | 1 | 2 | 13 | 1 | 1 |  |  |  |  |  |  | 145 | 50,8 | 18,8 | 26,9 | 16,9 | 73 | 52,5 | 18,6 | 35,8 | 16,2 | 1 | 1 | 0 | 1 | 1 | 1 | 1 | 1 |
| 200 | 0 | 1 | 1 | 5 | 1 | 2 | 31 | 19 | 32,6 | 100 | 1 | 2 | 3 | 0 | 1 |  |  |  |  |  |  | 10 | 135 | 107 | 43,3 | 62,4 | 9 | 322 | 235 | 264 | 122 | 0 | 0 | 1 | 0 | 0 | 0 | 0 | 0 |
| 201 | 0 | 3 | 1 | 3 | 1 | 1 | 57 | 38 | 81 | 81 | 1 | 3 | 13 | 0 | 1 |  |  |  |  |  |  | 19 | 1,55 | 1,03 | 1,03 | 0,67 | 19 | 1,64 | 1,24 | 1,24 | 1,26 | 1 | 0 | 1 | 1 | 0 | 0 | 0 | 0 |
| 202 | 0 | 1 | 1 | 3 | 1 | 1 | 21 | 14 | 49,4 | 76,2 | 1 | 4 | 10 | 0 | 1 |  |  |  |  |  |  | 7 |  |  |  |  | 7 |  |  |  |  | 0 | 0 | 0 | 0 | 0 | 0 | 0 | 0 |

Codes:
Reason for exclusion: 0, Included; 1, No intervention group; 2, Other language than English; 3, Relevant data not accessible
Setting: 1, Single center; 2, Unclear; 3, Multi-center
Study design: 1, Parallel; 2, Cross-over; 3, Factorial design
Type of intervention: 0, Pharmacological; 1, Physical; 2, Psychological
Duration: 1, <4 weeks; 2, 4-8 weeks; 3, >8-12 weeks; 4, >12 weeks
Chronic: 0, Non-chronic; 1, Chronic
Type of outcome: 1, Observable patient-reported outcome; 2, Non-observable patient-reported outcome; 3, Observer-reported outcomes dependent on patient cooperation; 4, Observer-reported outcomes that were not dependent on patient cooperation; 5, Laboratory outcomes
Outcome (cluster): 1, Anxiety; 2, Acute pain (because of procedure/surgery); 3, Chronic pain; 4, Medication use; 5, Insomnia; 6, Obesity; 7, Smoking; 8, Nausea; 9, Depression; 10, Diastolic blood pressure; 11, Phobia; 12, Asthma; 13, Other
No information to patients: 0, Patients informed or not stated; 1, Patients did not get information about placebo
Adequate sequence generation: 0, Unclear; 1, Yes; 2, No
Concealed allocation: 0, Not adequate or unclear; 1, Adequate
Clearly double-blinded: 0, Clearly not double-blinded; 1, Clearly double-blinded; 2, Unclear
Observer clearly blinded: 0, Not blinded or unclear; 1, Observer blinded
Low drop-out rate: 0: >15% or not stated; 1, Less than 15%
Protocol published: 0, No; 1, Yes
Reasonable sample size: 0, 49 or less; 1, Over 49

**References**

(1) Abikoff H, Hechtman L, Klein RG, Gallagher R, Fleiss K, Etcovitch J, et al. Social functioning in children with ADHD treated with long-term methylphenidate and multimodal psychosocial treatment. J Am Acad Child Adolesc Psychiatry. 2004;43(7):820-9.

(2) Adriaanse AH, Kollee LA, Muytjens HL, Nijhuis JG, de Haan AF, Eskes TK. Randomized study of vaginal chlorhexidine disinfection during labor to prevent vertical transmission of group B streptococci. Eur J Obstet Gynecol Reprod Biol. 1995;61(2):135-41.

(3) Alfano AP, Taylor AG, Foresman PA, Dunkl PR, McConnell GG, Conaway MR, et al. Static magnetic fields for treatment of fibromyalgia: a randomized controlled trial. J Altern Complement Med. 2001;7(1):53-64.

(4) Alkaissi A, Stalnert M, Kalman S. Effect and placebo effect of acupressure (P6) on nausea and vomiting after outpatient gynaecological surgery. Acta Anaesthesiol Scand. 1999;43(3):270-4.

(5) Alkaissi A, Evertsson K, Johnsson VA, Ofenbartl L, Kalman S. P6 acupressure may relieve nausea and vomiting after gynecological surgery: an effectiveness study in 410 women. Can J Anaesth. 2002;49(10):1034-9.

(6) Allen JJB, Schnyer RN, Hitt SK. The efficacy of acupuncture in the treatment of major depression in women. Psychol Sci. 1998;9(5):397-401.

(7) Allen JJB, Schnyer RN, Chambers AS, Hitt SK, Moreno FA, Manber R. Acupuncture for depression: a randomized controlled trial. J Clin Psychiatry. 2006;67(11):1665-73.

(8) Andersen A, Damm P, Tabor A, Pedersen IM, Harring M. Prevention of breast pain and milk secretion with bromocriptine after second-trimester abortion. Acta Obstet Gynecol Scand. 1990;69(3):235-8.

(9) Anderson BJ, Brackett J, Ho J, Laffel LM. An office-based intervention to maintain parent-adolescent teamwork in diabetes management. Impact on parent involvement, family conflict, and subsequent glycemic control. Diabetes Care. 1999;22(5):713-21.

(10) Antivalle M, Lattuada S, Salvaggio A, Paravicini M, Rindi M, Libretti A. Placebo effect and adaptation to noninvasive monitoring of BP. J Hum Hypertens. 1990;4(6):633-7.

(11) Antonio J, M. Colker C, C. Torina G, Shi Q, Brink W, Kalman D. Effects of a standardized guggulsterone phosphate supplement on body composition in overweight adults: a pilot study. Curr Ther Res. 1999;60:220-7.

(12) Ascher LM, Turner R. Paradoxical intention and insomnia: an experimental investigation. Behav Res Ther. 1979;17(4):408-11.

(13) Asmar R BS, Chaignon M, Guedon J, et al. Repeated measurement of non-invasive ambulatory blood pressure: distinction between reproducibility and the proper effect of placebo. Blood Press Monit. 1996;1:283-8.

(14) Aune A, Alraek T, LiHua H, Baerheim A. Acupuncture in the prophylaxis of recurrent lower urinary tract infection in adult women. Scand J Prim Health Care. 1998;16(1):37-9.

(15) Benedetti F, Amanzio M, Maggi G. Potentiation of placebo analgesia by proglumide. Lancet. 1995;346(8984):1231.

(16) Benedetti F, Amanzio M, Casadio C, Cavallo A, Cianci R, Giobbe R, et al. Control of postoperative pain by transcutaneous electrical nerve stimulation after thoracic operations. Ann Thorac Surg. 1997;63(3):773-6.

(17) Berg I, Forsythe I, Holt P, Watts J. A controlled trial of 'Senokot' in faecal soiling treated by behavioural methods. J Child Psychol Psychiatry. 1983;24(4):543-9.

(18) Biro P, Meier T, Cummins AS. Comparison of topical anaesthesia methods for venous cannulation in adults. Eur J Pain. 1997;1(1):37-42.

(19) Blackman S BA, Cove LM. The effect of imipramine on enuresis. Am J Psychiatry 1964;120:1194-5.

(20) Blades KJ, Patel S, Aidoo KE. Oral antioxidant therapy for marginal dry eye. Eur J Clin Nutr. 2001;55(7):589-97.

(21) Blanchard EB, Appelbaum KA, Radnitz CL, Michultka D, Morrill B, Kirsch C, et al. Placebo-controlled evaluation of abbreviated progressive muscle relaxation and of relaxation combined with cognitive therapy in the treatment of tension headache. J Consult Clin Psychol. 1990;58(2):210-5.

(22) Blanchard EB, Appelbaum KA, Radnitz CL, Morrill B, Michultka D, Kirsch C, et al. A controlled evaluation of thermal biofeedback and thermal biofeedback combined with cognitive therapy in the treatment of vascular headache. J Consult Clin Psychol. 1990;58(2):216-24.

(23) Block J. Effects of rational emotive therapy on overweight adults. Psychother Theo Res Pract. 1980;17(3):277-80.

(24) Bosley F, Allen TW. Stress management training for hypertensives: cognitive and physiological effects. J Behav Med. 1989;12(1):77-89.

(25) Bova JG, Bhattacharjee N, Jurdi R, Bennett WF. Comparison of no medication, placebo, and hyoscyamine for reducing pain during a barium enema. AJR Am J Roentgenol. 1999;172(5):1285-7.

(26) Bramston P, Spence SH. Behavioural versus cognitive social-skills training with intellectually-handicapped adults. Behav Res Ther. 1985;23(3):239-46.

(27) Brinkhaus B, Witt CM, Jena S, Linde K, Streng A, Wagenpfeil S, et al. Acupuncture in patients with chronic low back pain: a randomized controlled trial. Arch Intern Med. 2006;166(4):450-7.

(28) Cabrini L, Gioia L, Gemma M, Melloni G, Carretta A, Ciriaco P, et al. Acupuncture for diagnostic fiberoptic bronchoscopy: a prospective, randomized, placebo-controlled study. Am J Chin Med. 2006;34(3):409-15.

(29) Canino E, Cardona R, Monsalve P, Perez Acuna F, Lopez B, Fragachan F. A behavioral treatment program as a therapy in the control of primary hypertension. Acta Cient Venez. 1994;45(1):23-30.

(30) Carbajal R, Chauvet X, Couderc S, Olivier-Martin M. Randomised trial of analgesic effects of sucrose, glucose, and pacifiers in term neonates. BMJ. 1999;319(7222):1393-7.

(31) Carter JC, Olmsted MP, Kaplan AS, McCabe RE, Mills JS, Aime A. Self-help for bulimia nervosa: a randomized controlled trial. Am J Psychiatry. 2003;160(5):973-8.

(32) Chenard JR MS, Charest J, Jinxue L, et al. Evaluation of a behavioral intervention for chronic low-back pain: 'The interactional back school' [Évaluation d'un traitementcomportmental de la lombalgie chronique: 'I´ école interactionelle du dos']. Science et Comportement. 1991;21(4):225-39.

(33) Classen W, Feingold E, Netter P. Influence of sensory suggestibility on treatment outcome in headache patients. Neuropsychobiology. 1983;10(1):44-7.

(34) Colker CM, Kaiman DS, Torina GC, Perlis T, Street C. Effects of citrus aurantium extract, caffeine, and St. John's Wort on body fat loss, lipid levels, and mood states in overweight healthy adults. Curr Ther Res. 1999;60(3):145-53.

(35) Conn IG, Marshall AH, Yadav SN, Daly JC, Jaffer M. Transcutaneous electrical nerve stimulation following appendicectomy: the placebo effect. Ann R Coll Surg Engl. 1986;68(4):191-2.

(36) Corver K, Kerkhof M, Brussee JE, Brunekreef B, van Strien RT, Vos AP, et al. House dust mite allergen reduction and allergy at 4 yr: follow up of the PIAMA-study. Pediatr Allergy Immunol. 2006;17(5):329-36.

(37) Costello M, Ramundo M, Christopher NC, Powell KR. Ethyl vinyl chloride vapocoolant spray fails to decrease pain associated with intravenous cannulation in children. Clin Pediatr (Phila). 2006;45(7):628-32.

(38) Coyne PJ, MacMurren M, Izzo T, Kramer T. Transcutaneous electrical nerve stimulator for procedural pain associated with intravenous needlesticks. J Intraven Nurs. 1995;18(5):263-7.

(39) Crosby L, Palarski VA, Cottington E, Cmolik B. Iron supplementation for acute blood loss anemia after coronary artery bypass surgery: a randomized, placebo-controlled study. Heart Lung. 1994;23(6):493-9.

(40) Cupal DD, Brewer BW. Effects of relaxation and guided imagery on knee strength, reinjury anxiety, and pain following anterior cruciate ligament reconstruction. Rehabil Psychol. 2001;46(1):28-43.

(41) Davidson AM, Denney DR, Elliott CH. Suppression and substitution in the treatment of nailbiting. Behav Res Ther. 1980;18(1):1-9.

(42) De Sanctis MT, Incandela L, Belcaro G, Cesarone MR. Topical treatment of venous microangiopathy in patients with venous ulceration with Essaven gel--a placebo-controlled, randomized study. Angiology. 2001;52 Suppl 3:S29-34.

(43) Defrin R, Ariel E, Peretz C. Segmental noxious versus innocuous electrical stimulation for chronic pain relief and the effect of fading sensation during treatment. Pain. 2005;115(1-2):152-60.

(44) Dibble SL, Luce J, Cooper BA, Israel J, Cohen M, Nussey B, et al. Acupressure for chemotherapy-induced nausea and vomiting: a randomized clinical trial. Oncol Nurs Forum. 2007;34(4):813-20.

(45) Ditto B, France CR, Lavoie P, Roussos M, Adler PS. Reducing reactions to blood donation with applied muscle tension: a randomized controlled trial. Transfusion. 2003;43(9):1269-75.

(46) Ditto B, France CR. The effects of applied tension on symptoms in French-speaking blood donors: a randomized trial. Health Psychol. 2006;25(3):433-7.

(47) Double DB, Warren GC, Evans M, Rowlands RP. Efficacy of maintenance use of anticholinergic agents. Acta Psychiatr Scand. 1993;88(5):381-4.

(48) Dundee JW, Chestnutt WN, Ghaly RG, Lynas AG. Traditional Chinese acupuncture: a potentially useful antiemetic? Br Med J (Clin Res Ed). 1986;293(6547):583-4.

(49) Elliott CH, Denney DR. A multiple-component treatment approach to smoking reduction. J Consult Clin Psychol. 1978;46(6):1330-9.

(50) Erdogmus CB, Resch KL, Sabitzer R, Muller H, Nuhr M, Schoggl A, et al. Physiotherapy-based rehabilitation following disc herniation operation: results of a randomized clinical trial. Spine (Phila Pa 1976). 2007;32(19):2041-9.

(51) Espie CA, Brooks DN, Lindsay WR. An evaluation of tailored psychological treatment of insomnia. J Behav Ther Exp Psychiatry. 1989;20(2):143-53.

(52) Etringer BD, Cash TF, Rimm DC. Behavioral, affective, and cognitive effects of participant modeling and an equally credible placebo. Behav Ther. 1982;13(4):476-85.

(53) Etter JF, Laszlo E, Zellweger JP, Perrot C, Perneger TV. Nicotine replacement to reduce cigarette consumption in smokers who are unwilling to quit: a randomized trial. J Clin Psychopharmacol. 2002;22(5):487-95.

(54) Faas A, Chavannes AW, van Eijk JT, Gubbels JW. A randomized, placebo-controlled trial of exercise therapy in patients with acute low back pain. Spine (Phila Pa 1976). 1993;18(11):1388-95.

(55) Fanti L, Gemma M, Passaretti S, Guslandi M, Testoni PA, Casati A, et al. Electroacupuncture analgesia for colonoscopy. a prospective, randomized, placebo-controlled study. Am J Gastroenterol. 2003;98(2):312-6.

(56) Fisher P, McCarney R, Hasford C, Vickers A. Evaluation of specific and non-specific effects in homeopathy: feasibility study for a randomised trial. Homeopathy. 2006;95(4):215-22.

(57) Forster EL, Kramer JF, Lucy SD, Scudds RA, Novick RJ. Effect of TENS on pain, medications, and pulmonary function following coronary artery bypass graft surgery. Chest. 1994;106(5):1343-8.

(58) Foster KA, Liskin J, Cen S, Abbott A, Armisen V, Globe D, et al. The Trager approach in the treatment of chronic headache: a pilot study. Altern Ther Health Med. 2004;10(5):40-6.

(59) Foster NE, Thomas E, Barlas P, Hill JC, Young J, Mason E, et al. Acupuncture as an adjunct to exercise based physiotherapy for osteoarthritis of the knee: randomised controlled trial. BMJ. 2007;335(7617):436.

(60) Frank E, Kupfer DJ, Perel JM, Cornes C, Jarrett DB, Mallinger AG, et al. Three-year outcomes for maintenance therapies in recurrent depression. Arch Gen Psychiatry. 1990;47(12):1093-9.

(61) Frankel BL, Patel DJ, Horwitz D, Friedewald WT, Gaarder KR. Treatment of hypertension with biofeedback and relaxation techniques. Psychosom Med. 1978;40(4):276-93.

(62) Frega A, Stentella P, Di Renzi F, Gallo G, Palazzetti PL, Del Vescovo M, et al. Pain evaluation during carbon dioxide laser vaporization for cervical intraepithelial neoplasia: a randomized trial. Clin Exp Obstet Gynecol. 1994;21(3):188-91.

(63) Fuchs CZ, Rehm LP. A self-control behavior therapy program for depression. J Consult Clin Psychol. 1977;45(2):206-15.

(64) Godfrey S, Silverman M. Demonstration by placebo response in asthma by means of exercise testing. J Psychosom Res. 1973;17(4):293-7.

(65) Goodenough B, Kampel L, Champion GD, Laubreaux L, Nicholas MK, Ziegler JB, et al. An investigation of the placebo effect and age-related factors in the report of needle pain from venipuncture in children. Pain. 1997;72(3):383-91.

(66) Gracely RH, Dubner R, Wolskee PJ, Deeter WR. Placebo and naloxone can alter post-surgical pain by separate mechanisms. Nature. 1983;306(5940):264-5.

(67) Mayaux MJ, Guihard-Moscato ML, Schwartz D, Benveniste J, Coquin Y, Crapanne JB, et al. Controlled clinical trial of homoeopathy in postoperative ileus. Lancet. 1988;1(8584):528-9.

(68) Guglielmi RS, Roberts AH, Patterson R. Skin temperature biofeedback for Raynaud's disease: a double-blind study. Biofeedback Self Regul. 1982;7(1):99-120.

(69) Hall RG, Hanson RW, Borden BL. Permance of two self-managed treatments of overweight in university and community populations. J Consult Clin Psychol. 1974;42(6):781-6.

(70) Hanson RW, Borden BL, Hall SM, Hall RG. Use of programmed instruction in teaching self-management skills to overweight adults. Behav Ther. 1976;7(3):366-73.

(71) Hargreaves A, Lander J. Use of transcutaneous electrical nerve stimulation for postoperative pain. Nurs Res. 1989;38(3):159-61.

(72) Harrison RF, Blades M, De Louvois J, Hurley R. Doxycycline treatment and human infertility. Lancet. 1975;305(7907):605-7.

(73) Hashish I, Harvey W, Harris M. Anti-inflammatory effects of ultrasound therapy: evidence for a major placebo effect. Br J Rheumatol. 1986;25(1):77-81.

(74) Hashish I, Hai HK, Harvey W, Feinmann C, Harris M. Reduction of postoperative pain and swelling by ultrasound treatment: a placebo effect. Pain. 1988;33(3):303-11.

(75) Hawkins PJ, Liossi C, Ewart BW, Hatira P, Kosmidis VH, Varvutsi M. Hypnotherapy for control of anticipatory nausea and vomiting in children with cancer: preliminary findings. Psychooncology. 1995;4(2):101-6.

(76) Heinzl S, Andor J. Preoperative administration of prostaglandin to avoid dilatation-induced damage in first-trimester pregnancy terminations. Gynecol Obstet Invest. 1981;12(1):29-36.

(77) Helms JM. Acupuncture for the management of primary dysmenorrhea. Obstet Gynecol. 1987;69(1):51-6.

(78) Hong C-Z, Chen Y-C, Pon CH, Yu J. Immediate effects of various physical medicine modalities on pain threshold of an active myofascial trigger point. J Musculoskelet Pain. 1993;1(2):37-53.

(79) Hossmann V, FitzGerald GA, Dollery CT. Influence of hospitalization and placebo therapy on blood pressure and sympathetic function in essential hypertension. Hypertension. 1981;3(1):113-8.

(80) Hovell MF, Sipan CL, Blumberg EJ, Hofstetter CR, Slymen D, Friedman L, et al. Increasing Latino adolescents' adherence to treatment for latent tuberculosis infection: a controlled trial. Am J Public Health. 2003;93(11):1871-7.

(81) Hruby G, Ames C, Chen C, Yan Y, Sagar J, Baron P, et al. Assessment of efficacy of transcutaneous electrical nerve stimulation for pain management during office-based flexible cystoscopy. Urology. 2006;67(5):914-7.

(82) Hutton N, Wilson MH, Mellits ED, Baumgardner R, Wissow LS, Bonuccelli C, et al. Effectiveness of an antihistamine-decongestant combination for young children with the common cold: a randomized, controlled clinical trial. J Pediatr. 1991;118(1):125-30.

(83) Hyland MR, Webber-Gaffney A, Cohen L, Lichtman PT. Randomized controlled trial of calcaneal taping, sham taping, and plantar fascia stretching for the short-term management of plantar heel pain. J Orthop Sports Phys Ther. 2006;36(6):364-71.

(84) Hyman GJ, Stanley RO, Burrows GD, Horne DJ. Treatment effectiveness of hypnosis and behaviour therapy in smoking cessation: a methodological refinement. Addict Behav. 1986;11(4):355-65.

(85) Irvin JH, Domar AD, Clark C, Zuttemzeister PC, Friedman R. The effects of relaxation response training on menopausal symptoms. J Psychosom Obstet Gynaecol. 1996;17(4):202-7.

(86) Jacobs MA, Spilken AZ, Norman MM, Wohlberg GW, Knapp PH. Interaction of personality and treatment conditions associated with success in a smoking control program. Psychosom Med. 1971;33(6):545-56.

(87) Jacobson NS. Specific and nonspecific factors in the effectiveness of a behavioral approach to the treatment of marital discord. J Consult Clin Psychol. 1978;46(3):442-52.

(88) Kaptchuk TJ, Kelley JM, Conboy LA, Davis RB, Kerr CE, Jacobson EE, et al. Components of placebo effect: randomised controlled trial in patients with irritable bowel syndrome. BMJ. 2008;336(7651):999-1003.

(89) Karst M, Winterhalter M, Munte S, Francki B, Hondronikos A, Eckardt A, et al. Auricular acupuncture for dental anxiety: a randomized controlled trial. Anesth Analg. 2007;104(2):295-300.

(90) Karunakara S, Hammersley MS, Morris RJ, Turner RC, Holman RR. The fasting hyperglycaemia study: III. Randomized controlled trial of sulfonylurea therapy in subjects with increased but not diabetic fasting plasma glucose. Metabolism. 1997;46(12 Suppl 1):56-60.

(91) Kendall PC, Williams L, Pechacek TF, Graham LE, Shisslak C, Herzoff N. Cognitive-behavioral and patient education interventions in cardiac catheterization procedures: the Palo Alto Medical Psychology Project. J Consult Clin Psychol. 1979;47(1):49-58.

(92) Kerr AR, Drexel CA, Spielman AI. The efficacy and safety of 50 mg penicillin G potassium troches for recurrent aphthous ulcers. Oral Surg Oral Med Oral Pathol Oral Radiol Endod. 2003;96(6):685-94.

(93) Killeen TK, Brady KT, Gold PB, Simpson KN, Faldowski RA, Tyson C, et al. Effectiveness of naltrexone in a community treatment program. Alcohol Clin Exp Res. 2004;28(11):1710-7.

(94) Killen JD, Fortmann SP, Newman B, Varady A. Evaluation of a treatment approach combining nicotine gum with self-guided behavioral treatments for smoking relapse prevention. J Consult Clin Psychol. 1990;58(1):85-92.

(95) Kilmann PR, Milan RJ, Jr., Boland JP, Nankin HR, Davidson E, West MO, et al. Group treatment of secondary erectile dysfunction. J Sex Marital Ther. 1987;13(3):168-82.

(96) Klerman GL, Dimascio A, Weissman M, Prusoff B, Paykel ES. Treatment of depression by drugs and psychotherapy. Am J Psychiatry. 1974;131(2):186-91.

(97) Kober A, Scheck T, Greher M, Lieba F, Fleischhackl R, Fleischhackl S, et al. Prehospital analgesia with acupressure in victims of minor trauma: a prospective, randomized, double-blinded trial. Anesth Analg. 2002;95(3):723-7.

(98) Kokol R, Berger C, Haas J, Kopera D. Venous leg ulcers: no improvement of wound healing with 685-nm low level laser therapy. Randomised, placebo-controlled, double-blind study. Hautarzt. 2005;56(6):570-5.

(99) Kotani N, Kushikata T, Suzuki A, Hashimoto H, Muraoka M, Matsuki A. Insertion of intradermal needles into painful points provides analgesia for intractable abdominal scar pain. Reg Anesth Pain Med. 2001;26(6):532-8.

(100) Lander J, Fowler-Kerry S. TENS for children's procedural pain. Pain. 1993;52(2):209-16.

(101) Lee PCL, Jawad MSM, Hull JD, West WHL, Shaw K, Eccles R. The antitussive effect of placebo treatment on cough associated with acute upper respiratory infection. Psychosom Med. 2005;67(2):314-7.

(102) Leibing E, Leonhardt U, Koster G, Goerlitz A, Rosenfeldt JA, Hilgers R, et al. Acupuncture treatment of chronic low-back pain - a randomized, blinded, placebo-controlled trial with 9-month follow-up. Pain. 2002;96(1-2):189-96.

(103) Levine JD, Gordon NC. Influence of the method of drug administration on analgesic response. Nature. 1984;312(5996):755-6.

(104) Licciardone JC, Stoll ST, Fulda KG, Russo DP, Siu J, Winn W, et al. Osteopathic manipulative treatment for chronic low back pain: a randomized controlled trial. Spine (Phila Pa 1976). 2003;28(13):1355-62.

(105) Lick J. Expectancy, false galvanic skin response feedback, and systematic desensitization in the modification of phobic behavior. J Consult Clin Psychol. 1975;43(4):557-67.

(106) Lick JR, Heffler D. Relaxation training and attention placebo in the treatment of severe insomnia. J Consult Clin Psychol. 1977;45(2):153-61.

(107) Limoges MF, Rickabaugh B. Evaluation of TENS during screening flexible sigmoidoscopy. Gastroenterol Nurs. 2004;27(2):61-8.

(108) Lin JG, Lo MW, Wen YR, Hsieh CL, Tsai SK, Sun WZ. The effect of high and low frequency electroacupuncture in pain after lower abdominal surgery. Pain. 2002;99(3):509-14.

(109) Lincoln NB, Flannaghan T. Cognitive behavioral psychotherapy for depression following stroke: a randomized controlled trial. Stroke. 2003;34(1):111-5.

(110) Linde K, Streng A, Jurgens S, Hoppe A, Brinkhaus B, Witt C, et al. Acupuncture for patients with migraine: a randomized controlled trial. JAMA 2005;293(17):2118-25.

(111) Lindholm LH, Ekbom T, Dash C, Isacsson A, Scherstén B. Changes in cardiovascular risk factors by combined pharmacological and nonpharmacological strategies: the main results of the CELL Study. J Intern Med. 1996;240(1):13-22.

(112) Liossi C, Hatira P. Clinical hypnosis in the alleviation of procedure-related pain in pediatric oncology patients. Int J Clin Exp Hypn. 2003;51(1):4-28.

(113) Longo DJ, Clum GA, Yaeger NJ. Psychosocial treatment for recurrent genital herpes. J Consult Clin Psychol. 1988;56(1):61-6.

(114) Lorr M, McNair DM, Weinstein GJ, Michaux WW, Raskin A. Meprobamate and chlorpromazine in psychotherapy: some effects on anxiety and hostility of outpatients. Arch Gen Psychiatry. 1961;4(4):381-9.

(115) Macaluso AD, Connelly AM, Hayes WB, Holub MC, Ramsay MA, Suit CT, et al. Oral transmucosal fentanyl citrate for premedication in adults. Anesth Analg. 1996;82(1):158-61.

(116) Malcolm RE, Sillett RW, Turner JA, Ball KP. The use of nicotine chewing gum as an aid to stopping smoking. Psychopharmacology (Berl). 1980;70(3):295-6.

(117) Markland D, Hardy L. Anxiety, relaxation and anaesthesia for day-case surgery. Br J Clin Psychol. 1993;32 (Pt 4):493-504.

(118) Matros E, Rocha F, Zinner M, Wang J, Ashley S, Breen E, et al. Does gum chewing ameliorate postoperative ileus? Results of a prospective, randomized, placebo-controlled trial. J Am Coll Surg. 2006;202(5):773-8.

(119) May O, Hansen NC. Comparison of terbutaline, isotonic saline, ambient air and non-treatment in patients with reversible chronic airway obstruction. Eur Respir J. 1988;1(6):527-30.

(120) McLachlan DRC, Dalton AJ, Kruck TP, Bell MY, Smith WL, Kalow W, et al. Intramuscular desferrioxamine in patients with Alzheimer's disease. Lancet. 1991;337(8753):1304-8.

(121) McMillan CM. Transcutaneous electrical stimulation of Neiguan anti-emetic acupuncture point in controlling sickness following opioid analgesia in major orthopaedic surgery. Physiotherapy. 1994;80(1):5-9.

(122) Medici TC, Grebski E, Wu J, Hinz G, Wuthrich B. Acupuncture and bronchial asthma: a long-term randomized study of the effects of real versus sham acupuncture compared to controls in patients with bronchial asthma. J Altern Complement Med. 2002;8(6):737-50.

(123) Melchart D, Streng A, Hoppe A, Brinkhaus B, Witt C, Wagenpfeil S, et al. Acupuncture in patients with tension-type headache: randomised controlled trial. BMJ. 2005;331(7513):376-82.

(124) Moffett JA, Richardson PH, Frost H, Osborn A. A placebo controlled double blind trial to evaluate the effectiveness of pulsed short wave therapy for osteoarthritic hip and knee pain. Pain. 1996;67(1):121-7.

(125) Molsberger AF, Mau J, Pawelec DB, Winkler J. Does acupuncture improve the orthopedic management of chronic low back pain--a randomized, blinded, controlled trial with 3 months follow up. Pain. 2002;99(3):579-87.

(126) Moreland EC, Volkening LK, Lawlor MT, Chalmers KA, Anderson BJ, Laffel LM. Use of a blood glucose monitoring manual to enhance monitoring adherence in adults with diabetes: a randomized controlled trial. Arch Intern Med. 2006;166(6):689-95.

(127) Morey MC, Ekelund C, Pearson M, Crowley G, Peterson M, Sloane R, et al. Project LIFE: a partnership to increase physical activity in elders with multiple chronic illnesses. J Aging Phys Act. 2006;14(3):324-43.

(128) Morton AR, Fazio SM, Miller D. Efficacy of laser-acupuncture in the prevention of exercise-induced asthma. Ann Allergy. 1993;70(4):295-8.

(129) Murphy JK, Williamson DA, Buxton AE, Moody SC, Absher N, Warner M. The long-term effects of spouse involvement upon weight loss and maintenance. Behav Ther. 1982;13(5):681-93.

(130) Najnigier B PW, Zieniewicz K, Nyckowski P, et al. Zofran (ondansectron) in preventiong postoperative nausea and vomiting after laparoscopic cholecystectomy [Zofran w zapobieganiu nudnosciom i wymiotom po cholecystektomii laparoskopipwej]. Acta Endoscopica Polona. 1997;7(3):125-8.

(131) Nandi DN, Ajmany S, Ganguli H, Banerjee G, Boral GC, Ghosh A, et al. A clinical evaluation of depressives found in a rural survey in India. Br J Psychiatry. 1976;128:523-7.

(132) Nawrocki JD, Bell TJ, Lawrence WT, Ward JP. A randomized controlled trial of transurethral microwave thermotherapy. Br J Urol. 1997;79(3):389-93.

(133) Nicassio P, Bootzin R. A comparison of progressive relaxation and autogenic training as treatments for insomnia. J Abnorm Psychol. 1974;83(3):253-60.

(134) Nocella J, Kaplan RM. Training children to cope with dental treatment. J Pediatr Psychol. 1982;7(2):175-8.

(135) O'Brien B, Relyea MJ, Taerum T. Efficacy of P6 acupressure in the treatment of nausea and vomiting during pregnancy. Am J Obstet Gynecol. 1996;174(2):708-15.

(136) Parker JC, Smarr KL, Buckelew SP, Stucky-Ropp RC, Hewett JE, Johnson JC, et al. Effects of stress management on clinical outcomes in rheumatoid arthritis. Arthritis Rheum. 1995;38(12):1807-18.

(137) Parker JC, Smarr KL, Slaughter JR, Johnston SK, Priesmeyer ML, Hanson KD, et al. Management of depression in rheumatoid arthritis: a combined pharmacologic and cognitive-behavioral approach. Arthritis Rheum. 2003;49(6):766-77.

(138) Pelham WE, Murphy DA, Vannatta K, Milich R, Licht BG, Gnagy EM, et al. Methylphenidate and attributions in boys with attention-deficit hyperactivity disorder. J Consult Clin Psychol. 1992;60(2):282-92.

(139) Quayhagen MP, Quayhagen M, Corbeil RR, Roth PA, Rodgers JA. A dyadic remediation program for care recipients with dementia. Nurs Res. 1995;44(3):153-9.

(140) Rabkin JG, McGrath PJ, Quitkin FM, Tricamo E, Stewart JW, Klein DF. Effects of pill-giving on maintenance of placebo response in patients with chronic mild depression. Am J Psychiatry. 1990;147(12):1622-6.

(141) Rawling MJ, Wiebe ER. A randomized controlled trial of fentanyl for abortion pain. Am J Obstet Gynecol. 2001;185(1):103-7.

(142) Reading AE. The effects of psychological preparation on pain and recovery after minor gynaecological surgery: a preliminary report. J Clin Psychol. 1982;38(3):504-12.

(143) Ristikankare M, Hartikainen J, Heikkinen M, Janatuinen E, Julkunen R. Is routinely given conscious sedation of benefit during colonoscopy? Gastrointest Endosc. 1999;49(5):566-72.

(144) Robinson R, Darlow S, Wright SJ, Watters C, Carr I, Gadsby G, et al. Is transcutaneous electrical nerve stimulation an effective analgesia during colonoscopy? Postgrad Med J. 2001;77(909):445-6.

(145) Roongpisuthipong C, Panpakdee O, Boontawee A, Kulapongse S, Tanphaichitr V. Possible thermogenesis with dexfenfluramine. J Med Assoc Thai. 1999;82(2):150-9.

(146) Roscoe JA, Morrow GR, Bushunow P, Tian L, Matteson S. Acustimulation wristbands for the relief of chemotherapy-induced nausea. Altern Ther Health Med. 2002;8(4):56-7, 9-63.

(147) Roscoe JA, Matteson SE, Morrow GR, Hickok JT, Bushunow P, Griggs J, et al. Acustimulation wrist bands are not effective for the control of chemotherapy-induced nausea in women with breast cancer. J Pain Symptom Manage. 2005;29(4):376-84.

(148) Rosen GM, Glasgow RE, Barrera M, Jr. A controlled study to assess the clinical efficacy of totally self-administered systematic desensitization. J Consult Clin Psychol. 1976;44(2):208-17.

(149) Rossi A, Ziacchi V, Lomanto B. The hypotensive effect of a single daily dose of labetalol: a preliminary study. Int J Clin Pharmacol Ther Toxicol. 1982;20(9):438-45.

(150) Roughan PA, Kunst L. Do pelvic floor exercises really improve orgasmic potential? J Sex Marital Ther. 1981;7(3):223-9.

(151) Rowbotham MC, Davies PS, Verkempinck C, Galer BS. Lidocaine patch: double-blind controlled study of a new treatment method for post-herpetic neuralgia. Pain. 1996;65(1):39-44.

(152) Rybarczyk BD, Auerbach SM. Reminiscence interviews as stress management interventions for older patients undergoing surgery. Gerontologist. 1990;30(4):522-8.

(153) Roschke J, Wolf C, Muller MJ, Wagner P, Mann K, Grozinger M, et al. The benefit from whole body acupuncture in major depression. J Affect Disord. 2000;57(1-3):73-81.

(154) Rosler A, Otto B, Schreiber-Dietrich D, Steinmetz H, Kessler KR. Single-needle acupuncture alleviates gag reflex during transesophageal echocardiography: a blinded, randomized, controlled pilot trial. J Altern Complement Med. 2003;9(6):847-9.

(155) Sanders GE, Reinert O, Tepe R, Maloney P. Chiropractic adjustive manipulation on subjects with acute low back pain: visual analog pain scores and plasma beta-endorphin levels. J Manipulative Physiol Ther. 1990;13(7):391-5.

(156) Schallreuter KU, Moore J, Behrens-Williams S, Panske A, Harari M. Rapid initiation of repigmentation in vitiligo with Dead Sea climatotherapy in combination with pseudocatalase (PC-KUS). Int J Dermatol. 2002;41(8):482-7.

(157) Scharf HP, Mansmann U, Streitberger K, Witte S, Kramer J, Maier C, et al. Acupuncture and knee osteoarthritis: a three-armed randomized trial. Ann Intern Med. 2006;145(1):12-20.

(158) Scharff L, Marcus DA, Masek BJ. A controlled study of minimal-contact thermal biofeedback treatment in children with migraine. J Pediatr Psychol. 2002;27(2):109-19.

(159) Seer P, Raeburn JM. Meditation training and essential hypertension: a methodological study. J Behav Med. 1980;3(1):59-71.

(160) Senediak C, Spence SH. Rapid versus gradual scheduling of therapeutic contact in a family based behavioural weight control programme for children. Behav Cogn Psychother. 1985;13(4):265-87.

(161) Shen J, Wenger N, Glaspy J, Hays RD, Albert PS, Choi C, et al. Electroacupuncture for control of myeloablative chemotherapy-induced emesis: A randomized controlled trial. JAMA. 2000;284(21):2755-61.

(162) Sinaiko AR, Gomez-Marin O, Prineas RJ. Effect of low sodium diet or potassium supplementation on adolescent blood pressure. Hypertension. 1993;21(6, Part 2):989-94.

(163) Sipich JF, Russell RK, Tobias LL. A comparison of covert sensitization and “nonspecific” treatment in the modification of smoking behavior. J Behav Ther Exp Psychiatry. 1974;5(2):201-3.

(164) Spanos NP MT, Burgess CA. Comparison of multi-component hypnotic and non-hypnotic treatments for smoking. Contemp Hypn. 1995;12(1):12-9.

(165) Sprott H, Müller W. Efficacy of acupuncture in patients with fibromyalgia. Reumatologia. 1994;32(4):414-21.

(166) Stabholz A, Shapira J, Shur D, Friedman M, Guberman R, Sela MN. Local application of sustained-release delivery system of chlorhexidine in Down's syndrome population. Clin Prev Dent. 1991;13(5):9-14.

(167) Steinsbekk A, Bentzen N, Fonnebo V, Lewith GT. Randomized controlled trials on treatment by homeopaths and self-treatment with homeopathic medicines: design and protocol. J Altern Complement Med. 2004;10(6):1027-32.

(168) Stewart JE, Jacobs-Schoen M, Padilla MR, Maeder LA, Wolfe GR, Hartz GW. The effect of a cognitive behavioral intervention on oral hygiene. J Clin Periodontol. 1991;18(4):219-22.

(169) Stransky M, Rubin A, Lava NS, Lazaro RP. Treatment of carpal tunnel syndrome with vitamin B6: a double-blind study. South Med J. 1989;82(7):841-2.

(170) Straub WF, Spino MP, Alattar MM, Pfleger B, Downes JW, Belizaire MA, et al. The effect of chiropractic care on jet lag of Finnish junior elite athletes. J Manipulative Physiol Ther. 2001;24(3):191-8.

(171) Sumaya IC, Rienzi BM, Deegan JF, 2nd, Moss DE. Bright light treatment decreases depression in institutionalized older adults: a placebo-controlled crossover study. J Gerontol A Biol Sci Med Sci. 2001;56(6):M356-60.

(172) Tan SY, Poser EG. Acute pain in a clinical setting: effects of cognitive-behavioural skills training. Behav Res Ther. 1982;20(6):535-45.

(173) Tan SY, Bruni J. Cognitive-behavior therapy with adult patients with epilepsy: a controlled outcome study. Epilepsia. 1986;27(3):225-33.

(174) Tarçin O, Gürbüz AK, Poçan S, Keskin O, Demirtürk L. Acustimulation of the Neiguan point during gastroscopy: its effects on nausea and retching. Turk J Gastroenterol. 2004;15(4):258-62.

(175) Tarrier N, Yusupoff L, Kinney C, McCarthy E, Gledhill A, Haddock G, et al. Randomised controlled trial of intensive cognitive behaviour therapy for patients with chronic schizophrenia. BMJ. 1998;317(7154):303-7.

(176) Tashjian RZ, Banerjee R, Bradley MP, Alford W, Fadale PD. Zolpidem reduces postoperative pain, fatigue, and narcotic consumption following knee arthroscopy: a prospective randomized placebo-controlled double-blinded study. J Knee Surg. 2006;19(2):105-11.

(177) Theroux MC, West DW, Corddry DH, Hyde PM, Bachrach SJ, Cronan KM, et al. Efficacy of intranasal midazolam in facilitating suturing of lacerations in preschool children in the emergency department. Pediatrics. 1993;91(3):624-7.

(178) Thomas KB. General practice consultations: is there any point in being positive? Br Med J (Clin Res Ed). 1987;294(6581):1200-2.

(179) Thomas VJ, Dixon AL, Milligan P, Thomas N. Cognitive-behaviour therapy for the management of sickle cell disease pain: an evaluation of a community-based intervention. Br J Health Psychol. 1999;4(3):209-29.

(180) Thomas KS, Muir KR, Doherty M, Jones AC, O'Reilly SC, Bassey EJ. Home based exercise programme for knee pain and knee osteoarthritis: randomised controlled trial. BMJ. 2002a;325(7367):752.

(181) Thomas KS, Muir KR, Doherty M, Jones AC, O'Reilly SC, Bassey EJ. Home based exercise programme for knee pain and knee osteoarthritis: randomised controlled trial. BMJ. 2002b;325(7367):752-5.

(182) Tremeau ML F-RP, Taurnier F, Demouzon J. Protocol of cervical maturation by acupuncture [Protocole de maturation cervicale par acupuncture]. J Gynecol Obstet Biol Reprod. 1992;21:375-80.

(183) Tritrakarn T, Lertakyamanee J, Koompong P, Soontrapa S, Somprakit P, Tantiwong A, et al. Both EMLA and placebo cream reduced pain during extracorporeal piezoelectric shock wave lithotripsy with the Piezolith 2300. Anesthesiology. 2000;92(4):1049-54.

(184) Tsay SL, Chen ML. Acupressure and quality of sleep in patients with end-stage renal disease-a randomized controlled trial. Int J Nurs Stud. 2003;40(1):1-7.

(185) Tsay SL. Acupressure and fatigue in patients with end-stage renal disease-a randomized controlled trial. Int J Nurs Stud. 2004;41(1):99-106.

(186) Tuomilehto J, Voutilainen E, Huttunen J, Vinni S, Homan K. Effect of guar gum on body weight and serum lipids in hypercholesterolemic females. Acta Med Scand. 1980;208(1-2):45-8.

(187) Turner RM, Ascher LM. Controlled comparison of progressive relaxation, stimulus control, and paradoxical intention therapies for insomnia. J Consult Clin Psychol. 1979;47(3):500-8.

(188) Tyler DB. The influence of a placebo, body position and medication on motion sickness. Am J Physiol. 1946;146:458-66.

(189) Vlaeyen JW, Teeken-Gruben NJ, Goossens ME, Rutten-van Molken MP, Pelt RA, van Eek H, et al. Cognitive-educational treatment of fibromyalgia: a randomized clinical trial. I. Clinical effects. J Rheumatol. 1996;23(7):1237-45.

(190) Walton RE, Chiappinelli J. Prophylactic penicillin: effect on posttreatment symptoms following root canal treatment of asymptomatic periapical pathosis. J Endod. 1993;19(9):466-70.

(191) Wang B, Tang J, White PF, Naruse R, Sloninsky A, Kariger R, et al. Effect of the intensity of transcutaneous acupoint electrical stimulation on the postoperative analgesic requirement. Anesth Analg. 1997;85(2):406-13.

(192) Watzl H OR, Rist F, Cohen R. Placebo injections and alcohol surveillance in inpatient treatment of alcoholic women: An experimental study of two treatment characteristics [Placebo-injektionen und Alkoholkontrollen in der stationären Behandlung alkoholkranker Frauen - eine experimentalle Untersuchung zweier Behandlungsmerkmale]. Z Klin Psychol. 1986;15(4):333-45.

(193) Weingaertner AH. Self-administered aversive stimulation with hallucinating hospitalized schizophrenics. J Consult Clin Psychol. 1971;36(3):422-9.

(194) Werntoft E, Dykes AK. Effect of acupressure on nausea and vomiting during pregnancy. A randomized, placebo-controlled, pilot study. J Reprod Med. 2001;46(9):835-9.

(195) Whittaker CB, Hoy RM. Withdrawal of perphenazine in chronic schizophrenia. Br J Psychiatry. 1963;109:422-7.

(196) Wilcock A, Walton A, Manderson C, Feathers L, El Khoury B, Lewis M, et al. Randomised, placebo controlled trial of nebulised furosemide for breathlessness in patients with cancer. Thorax. 2008;63(10):872-5.

(197) Williams JM, Hall DW. Use of single session hypnosis for smoking cessation. Addict Behav. 1988;13(2):205-8.

(198) Wilson A, Davidson WJ, Blanchard R. Disulfiram implantation: a trial using placebo implants and two types of controls. J Stud Alcohol. 1980;41(5):429-36.

(199) Witt C, Brinkhaus B, Jena S, Linde K, Streng A, Wagenpfeil S, et al. Acupuncture in patients with osteoarthritis of the knee: a randomised trial. Lancet. 2005;366(9480):136-43.

(200) Wojciechowski F. Behavioral treatment of tension headache: a contribution of controlled outcome research methodology. Gedrag. 1984;12(5):16-30.

(201) Woods DL, Craven RF, Whitney J. The effect of therapeutic touch on behavioral symptoms of persons with dementia. Altern Ther Health Med. 2005;11(1):66-74.

(202) Yates RG, Lamping DL, Abram NL, Wright C. Effects of chiropractic treatment on blood pressure and anxiety: a randomized, controlled trial. J Manipulative Physiol Ther. 1988;11(6):484-8.
